# The transcriptional repressor PheR regulates uptake and catabolism of phenanthrene in *Sphingobium* sp. SHPJ-2

**DOI:** 10.1016/j.jbc.2026.113140

**Published:** 2026-05-12

**Authors:** Wanjing Wu, Wanxin Chen, Yuan Yang, Xu Qiu, Ping Xu, Hongzhi Tang, Weiwei Wang

**Affiliations:** State Key Laboratory of Microbial Metabolism, and School of Life Sciences & Biotechnology, Shanghai Jiao Tong University, Shanghai, P.R. China

**Keywords:** biosensors, IclR-family transcriptional repressor, phenanthrene, polycyclic aromatic hydrocarbons, sphingomonads

## Abstract

Phenanthrene (PHE) is a typical polycyclic aromatic hydrocarbon (PAH) and a persistent pollutant. Aerobic catabolic metabolism of PHE involves the coordinated regulation between substrate uptake and the energy-intensive initial oxidation steps. Although *Sphingobium* sp. SHPJ-2 can efficiently degrade PHE, the mechanism linking PHE sensing to the activation of its catabolic pathway remains unclear. Here, we demonstrate that PheR, an IclR-family transcriptional repressor, links PHE availability to transcriptional activation of the phenanthrene-degradation operon in strain SHPJ-2. EMSA and DNase I footprinting analyses indicate that PheR specifically binds to P_phnA1_, the promoter upstream of *phnA1* that drives transcription of the phenanthrene-degradation operon. Through site-directed mutagenesis combined with biolayer interferometry, we identify three residues (R63, R73, and R78) as critical for DNA binding and define 5′-GCAACG-3′ as the minimal recognition motif for PheR. Importantly, PHE acts as an effector molecule that diminishes PheR binding to the P_phnA1_ promoter DNA, supporting a model in which PHE availability triggers derepression of the catabolic operon. Consistently, deletion of *pheR* accelerates PHE degradation and markedly upregulates transcription of phenanthrene-degradation genes. Comparative transcriptomic analysis further indicates that PheR exerts broader downstream effects beyond the core catabolic cluster, including modulation of outer membrane–associated functions such as TonB/ExbBD-dependent energy transduction and envelope homeostasis. Collectively, this work links PHE availability to transcriptional control of phenanthrene catabolism in *Sphingobium* and identifies PheR as a potentially portable regulatory element for developing PAH-responsive whole-cell biosensors.

Polycyclic aromatic hydrocarbons (PAHs) are among the most widespread and recalcitrant organic pollutants arising from petroleum processing, combustion, and industrial activities (1). Phenanthrene (PHE) is classified as a priority contaminant because of its environmental persistence, bioaccumulation potential, and mutagenic and carcinogenic properties (2). Although many microorganisms can utilize PAHs as carbon sources under aerobic conditions, the extreme hydrophobicity of PAHs imposes major barriers to bioavailability and transport, while the initial oxygenation steps are energetically costly and can impose redox and envelope stress, necessitating tight transcriptional control to ensure efficient and economical catabolic initiation (3). The first step of PAH degradation is typically catalyzed by multicomponent aromatic ring-hydroxylating dioxygenases (RHDs) or monooxygenases, after which central intermediates are funneled into the tricarboxylic acid cycle (4, 5). These pathways are usually organized into inducible gene clusters whose expression is governed by dedicated transcriptional regulators to coordinate substrate uptake with oxidative capacity and downstream metabolic flux, thereby minimizing unnecessary energetic expenditure (6, 7).

Members of the IclR family of transcriptional regulators are key players in controlling the catabolism of various aromatic compounds, typically functioning as repressors that respond to pathway substrates or intermediates (8, 9). Numerous IclR regulators involved in the degradation of benzoate, p-hydroxybenzoate, and other small aromatic acids have been well characterized (10, 11, 12, 13). In contrast, the regulatory mechanisms governing PAH catabolism—particularly for structurally complex and highly hydrophobic compounds such as phenanthrene—remain poorly defined (14). A key unresolved question is how PHE availability is sensed and subsequently translated into coordinated transcriptional activation of both uptake systems and the energy-intensive initial oxidation modules.

We previously isolated *Sphingobium* sp. SHPJ-2, which efficiently degrades multiple PAHs and completely mineralizes 400 mg/L PHE within 81 h (9). However, the regulatory network controlling expression of its PHE-catabolic gene cluster remains uncharacterized. Transcriptomic analyses under PHE-inducing conditions indicated that an adjacent IclR-family transcriptional regulator, PheR, may govern this catabolic module. Here, we systematically dissect the transcriptional regulation of PHE catabolism in strain SHPJ-2, with emphasis on the function and molecular mechanism of PheR. By combining genetics approaches with biochemical DNA-binding assays and transcriptomic analysis, we define the operator motif and critical DNA-binding residues of PheR and demonstrate that phenanthrene-dependent derepression serves as a key mechanism coupling pollutant sensing to catabolic operon activator. Together, our results establish a mechanistic framework for PAH-responsive regulation in *Sphingobium* and highlight PheR as a potentially portable regulatory element for developing whole-cell biosensors and engineering PAH catabolism.

## Results

### Organization and transcriptional analysis of the phenanthrene degradation gene cluster of *Sphingobium* sp. SHPJ-2

**Figure 1:**
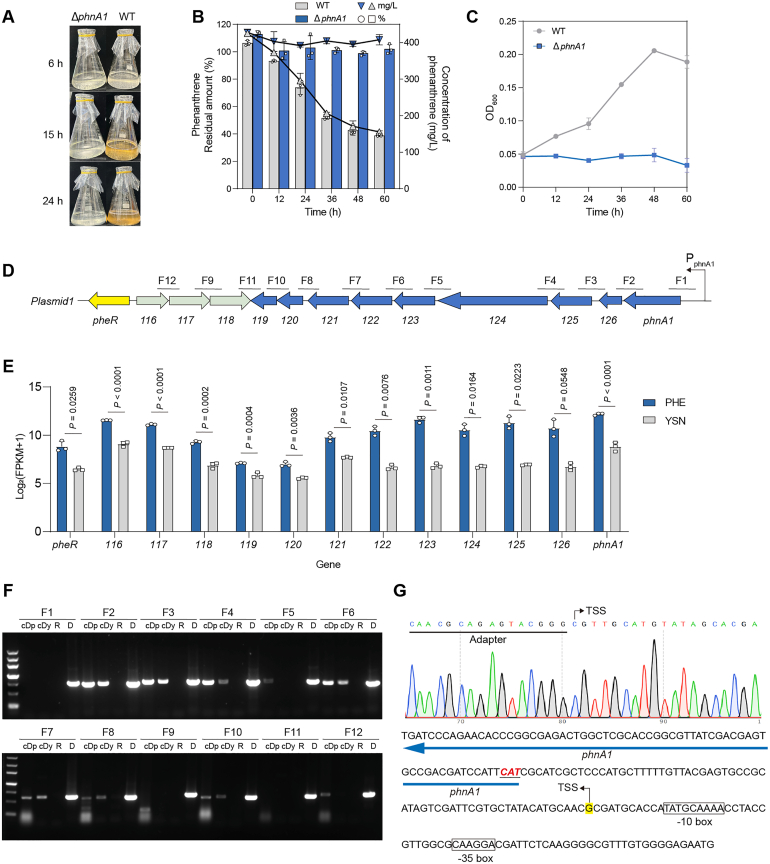
**Profiles of the phenanthrene-degradation gene cluster in *Sphingobium* sp. SHPJ-2.***A*, representative cultures of wild-type SHPJ-2 and the Δ*phnA1* mutant grown in MSM with phenanthrene as the sole carbon source. Wild-type SHPJ-2 showed a visible *yellow* coloration during growth on phenanthrene, consistent with active PHE metabolism and degradation, whereas this phenotype was not observed in the Δ*phnA1* mutant. *B*, phenanthrene degradation by wild-type SHPJ-2 *versus* the *ΔphnA1* mutant. *Blue* (*ΔphnA1*) and *gray* (WT) dots represent phenanthrene concentration over time (*left y-axis*), whereas bars of corresponding colors indicate residual phenanthrene levels (*right y-axis*). *C*, growth curves of wild-type SHPJ-2 and the *ΔphnA1* mutant in MSM with phenanthrene as the sole carbon source. *Blue* represents *ΔphnA1*, and *gray* represents the wild-type strain. *D*, schematic representation of the key phenanthrene degradation gene cluster in *Sphingobium* sp. SHPJ-2. The transcriptional units are illustrated as arrows of different colors. The amplification fragments (F1–F12) for the identification of transcriptional units are shown above the phenanthrene-degradation gene cluster. *E*, transcriptomic analysis of gene expression within the phenanthrene degradation cluster under two growth conditions: YSN (MSM supplemented with sodium acetate as the sole carbon source; *gray*) and PHE (MSM supplemented with phenanthrene as the sole carbon source; *blue*). Statistical significance was assessed using two-tailed unpaired *t* test with Welch’s correction. Data are presented as the mean ± SD of three biological replicates. *p* values are indicated above each genotype. *F*, co-transcription analysis of the phenanthrene degradation gene cluster using RT-PCR with genomic DNA (gDNA), cDNA, and total RNA as templates. All 12 intergenic fragments—including regions upstream and downstream of the target genes—were successfully amplified from gDNA with no nonspecific bands, confirming primer specificity and amplification efficiency. No amplification occurred using total RNA, indicating the absence of genomic DNA contamination. cD_p_ represents cDNA from SHPJ-2 grown in MSM with phenanthrene as the sole carbon source; cD_y_ represents cDNA from SHPJ-2 grown in MSM with sodium acetate; *R* denotes total RNA; D denotes genomic DNA of the gene cluster. *G*, mapping of the transcription start site (TSS) of the phenanthrene degradation operon by 5′ RACE. The TSS (highlighted in *yellow*) is a cytosine nucleotide located 63 bp upstream of the *phnA1* start codon. The start codon (ATG) is shown in *red bold italic*, and the *blue arrow* indicates the transcriptional direction of *phnA1*. The predicted −10 and −35 promoter elements of P_phnA1_, as identified by the phiSITE, BacPP, and BDGP online tools, are indicated.

Based on prior functional predictions of candidate ring-hydroxylating dioxygenases (RHDs) and cytochrome P450 monooxygenases (P450s), targeted gene deletions were carried out to verify their roles in PHE degradation (9). In the wild-type strain SHPJ-2, growth on PHE was accompanied by a visible yellow coloration of the culture, indicating active PHE metabolism (Fig. 1*A*). In contrast, deletion of *phnA1* (locus tag: plasmid1_127), which encodes a dioxygenase on plasmid-1, severely impaired both growth and PHE degradation when PHE served as the sole carbon source. In addition, no yellow coloration was observed in the Δ*phnA1* mutant (Fig. 1, *B* and *C*), further supporting the essential role of *phnA1* in PHE metabolism. By comparison, deletion of other candidate RHD and P450 genes did not produce comparable defects (Fig. S1), indicating that *phnA1* is the primary enzyme required for efficient PHE degradation in SHPJ-2. Consistent with the essential role of PhnA1 in phenanthrene catabolism, structural similarity analysis identified four SHPJ-2 proteins with the highest structural similarity to PhnA1 (locus tags plasmid1_82, plasmid1_50, plasmid1_86, and plasmid1_79). These proteins are predominantly annotated as Rieske 2Fe-2S-containing aromatic oxygenase/dioxygenase proteins, suggesting that they may represent a structurally related group of oxidative enzymes potentially involved in aromatic compound metabolism (Fig. S2). These results establish PhnA1 as the core enzyme required for efficient phenanthrene catabolism in SHPJ-2.

To define the genetic basis of phenanthrene (PHE) degradation in *Sphingobium* sp. SHPJ-2, we performed hybrid whole-genome sequencing using Illumina NovaSeq and Oxford Nanopore platforms, followed by assembly with Unicycler and polishing with Pilon. The assembled genome comprises two chromosomes and four plasmids (Fig. S3). Functional annotation revealed that multiple aromatic-compound catabolic loci, including candidate PAH degradation clusters, are concentrated on the largest plasmid (plasmid-1, 258,039 bp), indicating that this replicon serves as a major genetic reservoir for PHE metabolism in SHPJ-2. Genome analysis revealed that the phenanthrene catabolic locus spans genes *plasmid1_116–plasmid1_127* on plasmid-1 and includes the aromatic ring-hydroxylating dioxygenase α-subunit gene *phnA1* (*plasmid1_127*) (Fig. 1*D*). Transcriptomic analysis showed that this locus is weakly expressed in the absence of PHE but is strongly and coordinately induced upon PHE exposure, indicating stringent substrate-responsive regulation. Notably, *pheR*, located immediately downstream of the cluster and encoding an IclR-family transcriptional regulator, was upregulated approximately 14-fold under PHE induction (Fig. 1*E*), implicating it in control of this catabolic pathway.

To determine whether this cluster is transcribed as a single operon, RT-PCR was performed using genomic (gDNA), complementary DNA (cDNA), and total RNA as templates. Twelve amplicons spanning intergenic regions within the cluster and flanking sequences were designed. All fragments were amplified from gDNA, confirming primer specificity, whereas no products were detected from total RNA, excluding gDNA contamination. Using cDNA as a template, all fragments were successfully amplified except fragments 1 and 11, suggesting that most genes within the cluster are co-transcribed and induced by PHE (Fig. 1*F*). The transcription start site (TSS) of the cluster was subsequently mapped by 5′ rapid amplification of cDNA ends using RNA from PHE-grown cells. The TSS was identified at a cytosine residue located 63 bp upstream of the translational start codon (Fig. 1*G*). Predicted −10 and −35 promoter elements of the *phnA1* promoter (P_phnA1_) are shown in Figure 1*G*, based on analyses using phiSITE (15), BacPP (16), and BDGP (17).

### PheR specifically binds to P_phnA1_

**Figure 2:**
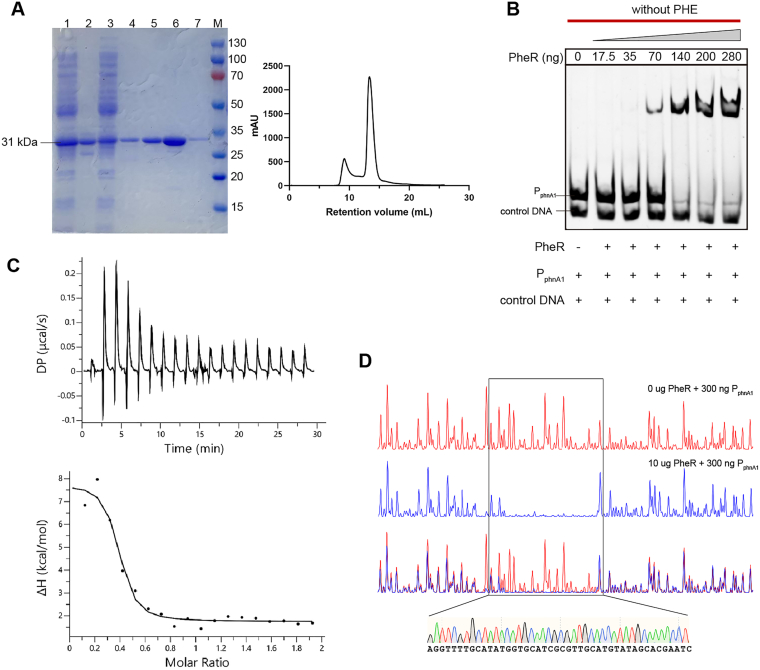
**PheR specifically binds to the *phnA1* promoter region.***A*, SDS–PAGE analysis and gel filtration of recombinant PheR. Recombinant PheR was overexpressed in *Escherichia coli* BL21(DE3) using the pET-28a expression vector and subsequently purified. Coomassie Brilliant *Blue* R-250 staining of the SDS–PAGE gel shows a single prominent band at approximately 30.4 kDa, consistent with the predicted molecular mass of PheR. Lane M contains the protein molecular weight markers. Lanes 1 to 5 represent the soluble fraction (supernatant), insoluble fraction (pellet), flow-through, 50 mM imidazole wash, and 200 mM imidazole elution, respectively. *B*, electrophoretic mobility shift assay (EMSA) showing the interaction between PheR and the promoter of *phnA1* (P_phnA1_). *C*, isothermal titration calorimetry (ITC) of PheR binding to the P_phnA1_. Purified PheR (20 μM, cell) was titrated with P_phnA1_ DNA (200 μM, syringe). The *upper panel* shows the raw differential power signal (DP) for sequential injections, and the *lower panel* shows the integrated heats plotted against the molar ratio and fitted to a binding model. The fit yielded an apparent dissociation constant K_*d*_ = 2.33 × 10^−7^ ± 1.22 × 10^−9^ M, an apparent stoichiometry parameter N = 0.354 ± 0.016, and an enthalpy change ΔH = 6.03 ± 0.44 kcal mol^−1^. *D*, DNase I footprinting analysis of PheR binding to the P_phnA1_. A FAM-labeled noncoding-strand probe was incubated with (*blue trace*) or without (*red trace*) 10 μg PheR. The protected region, outlined by solid lines, delineates a 44-bp GC-rich operator sequence. The nucleotide sequence of the protected region is shown below.

Recombinant PheR was heterologously expressed in *Escherichia coli* BL21(DE3) from pET-28a and purified to homogeneity. SDS–PAGE analysis revealed a single band of approximately 31 kDa, which matches the predicted molecular mass of 30.4 kDa (Fig. 2*A*). Electrophoretic mobility shift assays (EMSA) showed that the addition of 70 ng PheR produced a distinct PheR–DNA complex with the P_phnA1_. Higher concentrations of PheR led to a progressive, concentration-dependent increase in the shifted complex, while no shift was observed with a nonspecific DNA control (Fig. 2*B*), indicating specific recognition of P_phnA1_ by PheR. Direct interaction between PheR and DNA was confirmed by isothermal titration calorimetry (ITC): titration of 200 μM P_phnA1_ DNA into 20 μM PheR produced a characteristic binding thermogram (Fig. 2*C*). DNase I footprinting further localized the binding site to a 44-bp protected region (GATTCGTGCTATACATGCAACGCGATGCACCATATGCAAAACCT), spanning positions +23 to −21 relative to the *phnA1* transcription start site (Fig. 2*D*).

### Key residues required for PheR–P_phnA1_ binding

**Figure 3:**
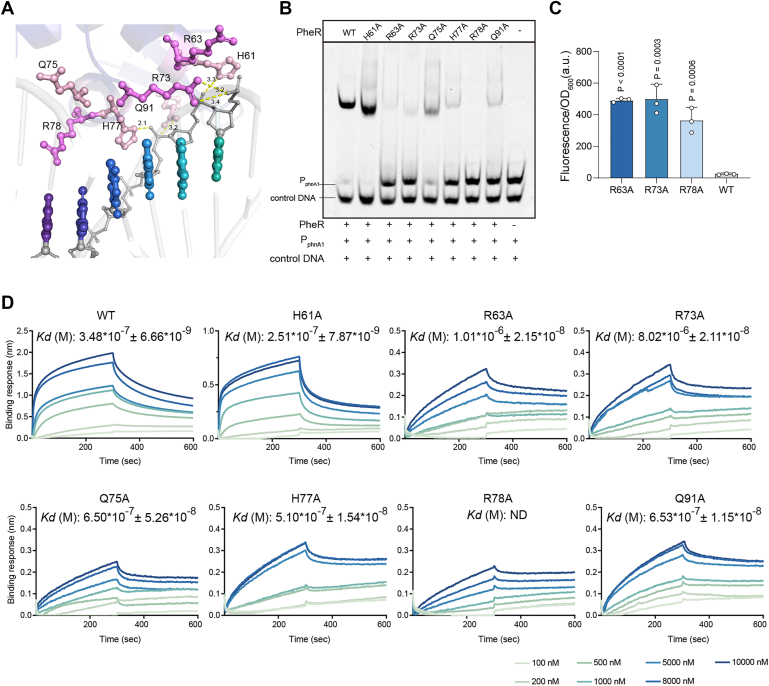
**Key amino acid residue mutation of PheR.***A*, structural docking model of the PheR–DNA (GCTTCG) complex highlighting key interface contacts. *B*, EMSA was performed with the P_phnA1_ incubated with 400 ng of purified wild-type PheR (WT) or specified single-site variants (H61A, R63A, R73A, Q75A, R78A, H77A, Q91A). Shifted DNA–protein complexes and free probes are shown, with a nonspecific DNA probe included as a negative control. *C*, binding affinity of the mutant PheR protein to P_phnA1_ in strain SHPJ-2, quantified by fluorescence intensity. Statistical significance was assessed using two-tailed unpaired *t* test with Welch’s correction. Data are presented as the mean ± SD of three biological replicates. *p* values are indicated above each genotype. *D*, biolayer interferometry (BLI) analysis of PheR (WT) or specified single-site variants (H61A, R63A, R73A, Q75A, R78A, H77A, and Q91A) binding to a biotin-labeled P_phnA1_ fragment. Sensorgrams show concentration-dependent binding over a range of 100–10,000 nM PheR.

To identify amino acid residues potentially involved in PheR-DNA interaction, we analyzed the structural docking model of PheR with P_phnA1_. As shown in Figure 3*A*, several residues in PheR were predicted to form hydrogen bonds with DNA bases (dashed lines, distances in Å). Based on this model, seven residues, H61, R63, R73, Q75, H77, R78, and Q91, were selected for further examination by site-directed mutagenesis (Fig. S4).

EMSA results showed that the H61A mutant bound to P_phnA1_ at levels similar to the wild type. In contrast, DNA binding affinities of Q75A, H77A, and Q91A were markedly weakened, whereas R63A, R73A, and R78A exhibited little or no detectable binding, indicating that these three arginine residues are crucial for stabilizing the DNA-protein complex (Fig. 3*B*). We further assessed PheR function *in vivo* using an SHPJ-2Δ*pheR*-based fluorescent reporter system. In this assay, plasmids carrying an RFP reporter driven by P_phnA1_ together with either wild-type PheR or the corresponding mutant alleles (R63A, R73A, and R78A) were introduced into SHPJ-2Δ*pheR*. Consistent with the *in vitro* assays, each mutation markedly impaired PheR-mediated repression of P_phnA1_ (Fig. 3*C*).

Increasing the protein concentrations resulted in only weak binding for R63A and R73A mutants, whereas R78A exhibited no binding even at elevated concentrations (Fig. S5). Since the arginine side chain is positively charged and capable of forming multiple hydrogen bonds, the R→A mutation would simultaneously eliminate potential electrostatic interactions and specific hydrogen bond contacts, thus weakening the typical anchoring interaction the with DNA phosphate backbone. To provide independent quantitative support, we further measured the binding of PheR and its derivatives to the same DNA probe using biolayer interferometry (BLI). BLI enabled quantification of the binding kinetics between the mutant proteins and P_phnA1_. For wild-type PheR, interaction kinetics were measured across concentrations of 100– 10,000 nM, yielding a dissociation constant (K_*d*_) of 3.48 × 10^−7^ ± 6.66 × 10^−9^ M, an association rate (k_*on*_) of 7698 ± 134.2 M^−1^ s^−1^, and a dissociation rate (k_*off*_) of 2.68 × 10^−3^ ± 2.09 × 10^−5^ s^−1^. The Hill coefficient (nH = 1.95) indicates cooperative binding of PheR to P_phnA1_. Comparison of K_*d*_ values revealed that the affinities of R63A, R73A, and R78A decreased substantially relative to the wild type (Fig. 3*D*). Collectively, these results indicate that the three amino acid residues R63, R73 and R78 are critical for PheR function.

### The 5′-GCAACG-3′ motif is essential for PheR binding to the P_phnA1_

**Figure 4:**
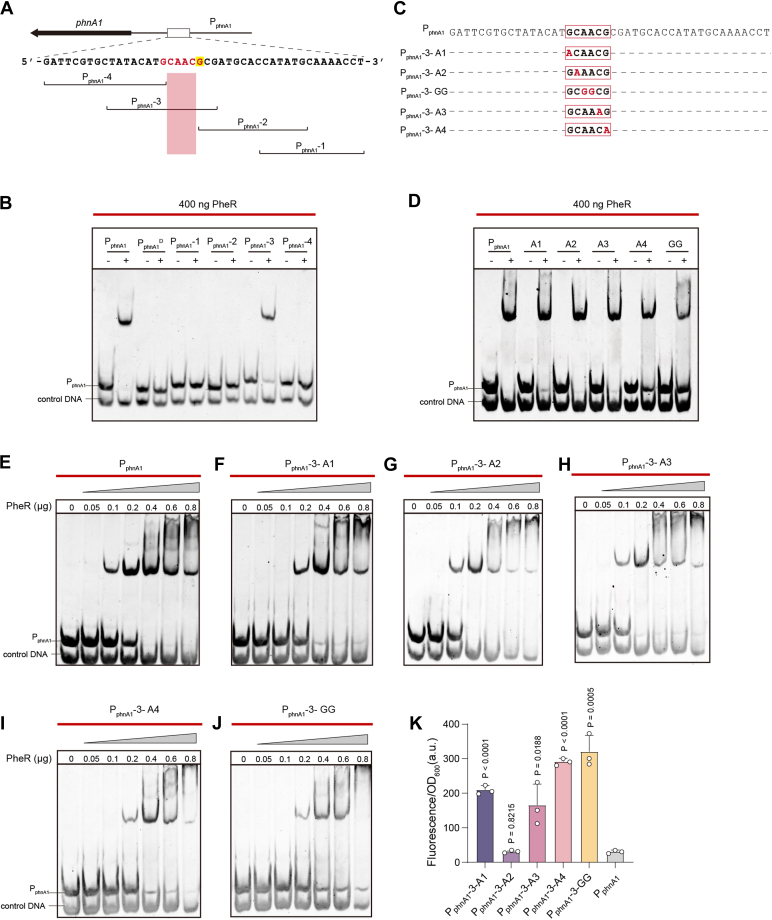
**Mutational analysis of PheR binding sites.***A*, schematic diagram of the fragment P_phnA1_ region. The nucleotide sequence and DNA elements of fragment are shown at the *top*. The transcription start site (TSS) is highlighted in a *yellow box*. The key sequence 5′-GCAACG-3′ is shown in the *red box*. *B*, EMSA of subfragments P_phnA1_-1 to P_phnA1_-4 in *panel A* with PheR. *C*, schematic diagram of the mutant nucleotides of P_phnA1_-3. *D*, EMSA of wild-type and mutant sequences of P_phnA1_-3-A1 to P_phnA1_-3-A4 with PheR. *E–J*, EMSA of the fragments P_phnA1_ (*E*), P_phnA1_-3-A1 (*F*), P_phnA1_-3-A2 (*G*), P_phnA1_-3-A3 (*H*), P_phnA1_-3-A4 (*I*) and P_phnA1_-3-GG (*J*) in the presence of different PheR concentrations. *K*, binding affinity of the mutant DNA to P_phnA1_ in SHPJ-2, quantified by fluorescence intensity. Statistical significance was assessed using two-tailed unpaired *t* test with Welch’s correction. Data are presented as the mean ± SD of three biological replicates. *p* values are indicated above each genotype. For the EMSA assays shown in *panelsB* and *D–J*, A total of 40 ng of each DNA fragment, including P_phnA__1_-derived fragments and control DNA where indicated, was used in each reaction.

Subsequently, to further examine PheR binding sites, this protected 44-bp fragment (GATTCGTGCTATACATGCAACGCGATGCACCATATGCAAAACCT) was subdivided into the four subsegments, designated P_phnA1_-1 to P_phnA1_-4. P_phnA1_^D^ represents the construct in which this 44-bp fragment was deleted (Fig. 4*A*). EMSA results showed that only P_phnA1_-3 exhibited a mobility shift in the presence of PheR, whereas the other subfragments did not (Fig. 4*B*). This result suggests that the core PheR-binding determinant is located within the central region covered by P_phnA1_-3.

To determine which sequence is essential for PheR binding, various mutant P_phnA1_ DNA fragments were tested. Individual nucleotides within the 5′-GCAACG-3′ sequence were mutated to generate 5′-ACAACG-3′ (P_phnA1_-3-A1), 5′-GAAACG-3′ (P_phnA1_-3-A2), 5′-GCGGCG-3′ (P_phnA1_-3-GG), 5′-GCAAAG-3′ (P_phnA1_-3-A3), and 5′-GCAACA-3′ (P_phnA1_-3-A4) (Fig. 4*C*). Although all mutant fragments formed DNA–PheR complexes, analysis of complex intensity and unbound DNA levels indicated that P_phnA1_-3-GG had the strongest effect on complex formation, significantly impairing PheR–DNA binding, followed by P_phnA1_-3-A1 and P_phnA1_-3-A4 (Fig. 4*D*). Gradient combined analysis showed that P_phnA1_-3-A2 and P_phnA1_-3-A3 exhibited binding behavior similar to that of wild-type P_phnA1_, forming a protein–DNA complex with only 0.1 μg PheR and 40 ng of the DNA fragment (Fig. 4, *E* and *G* and *H*). In contrast, under identical DNA concentrations, P_phnA1_-3-GG, P_phnA1_-3-A1, and P_phnA1_-3-A4 required at least 0.2 μg PheR to form detectable complexes (Fig. 4, *F* and *I* and *J*). Consistent with the EMSA results, we further evaluated the effects of promoter mutations *in vivo* using reporter plasmids. These plasmids carried different single-nucleotide-mutated P_phnA1_ variants upstream of RFP together with wild-type PheR. They were introduced into SHPJ-2Δ*pheR*, and RFP fluorescence output was used to assess the effect of each promoter mutation on PheR-mediated repression. Compared with P_phnA1_, the P_phnA1_-3-A1, P_phnA1_-3-A3, P_phnA1_-3-A4, and P_phnA1_-3-GG mutations alleviated PheR-dependent repression to different extents, with P_phnA1_-3-A4 and P_phnA1_-3-GG showing the strongest effects, whereas the P_phnA1_-3-A2 mutation behaved similarly to the wild-type promoter (Fig. 4*K*). Therefore, although single-nucleotide substitutions affected PheR-mediated repression to varying degrees, the six-nucleotide core sequence was essential for efficient PheR binding.

### Phenanthrene affects PheR binding

**Figure 5:**
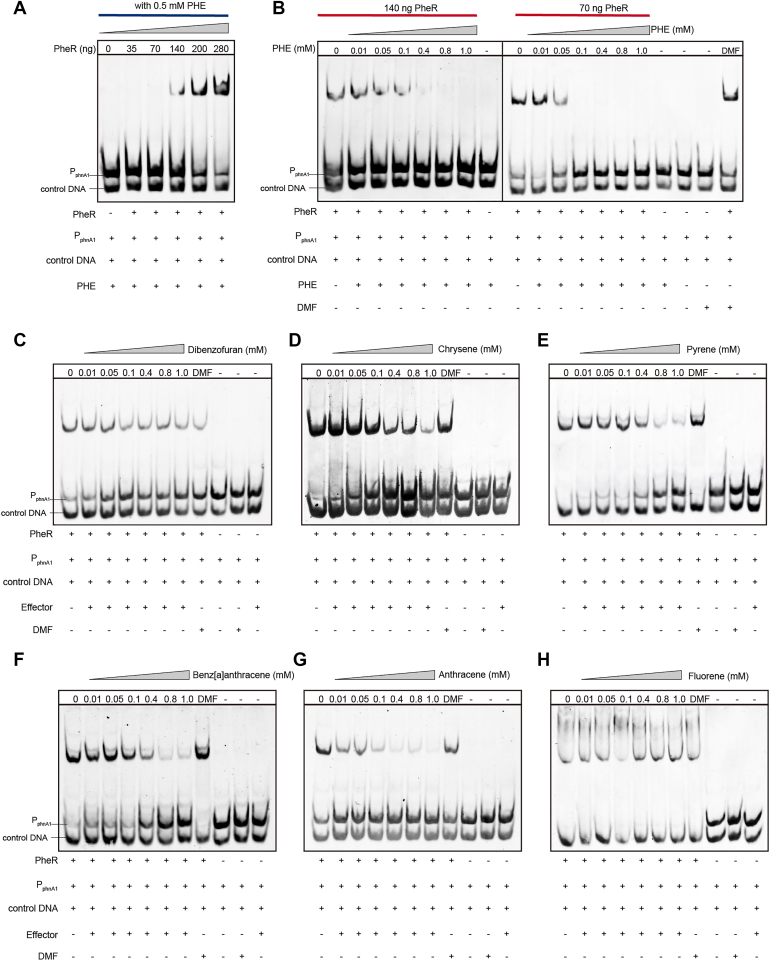
**Determination of the PheR effector.***A*, electrophoretic mobility shift assays (EMSAs) showing the binding of PheR to the P_phnA1_ in the presence of 0.5 mM phenanthrene as the effector. *B*, EMSAs performed with 70 ng and 140 ng purified PheR. DNA fragments containing the P_phnA1_ were incubated with PheR in the presence of gradually increasing concentrations of phenanthrene (0 mM–1.0 mM; MW = 178.23 g/mol). *C–H*, EMSAs assessing PheR binding to the P_phnA1_ in the presence of different potential effectors, each tested across a concentration range of 0 mM to 1.0 mM: (*C*) Dibenzofuran, (*D*) Chrysene, (*E*) Pyrene, (*F*) Benz[a]anthracene, (*G*) Anthracene and (*H*) Fluorene. DMF, N,N-dimethylformamide, was used as the solvent control for PHE.

The effects of phenanthrene on PheR binding to the P_phnA1_ was measured by EMSA. As shown in Figures 2*B* and 5*A*, 0.5 mM PHE strongly inhibited the binding of 70 ng PheR to the P_phnA1_ DNA fragment. With increasing PHE concentrations from 0.01 mM to 1.0 mM, the abundance of the PheR–DNA complex gradually decreased, indicating that PHE significantly weakens the interaction between PheR and P_phnA1_. A similar trend was observed at varying PheR concentrations (Fig. 5*B*). Additionally, EMSA was performed to evaluate several structural analogs, including dibenzofuran, chrysene, pyrene, benz[a]anthracene, anthracene, and fluorene were tested by EMSA. The results showed that dibenzofuran, chrysene, and fluorene had no detectable effect on the PheR–P_phnA1_ interaction (Fig. 5, *C*, *D*, and *H*). In contrast, although pyrene, benz[a]anthracene, and anthracene reduced the PheR–DNA complex formation, a shifted band remained detectable even at 1.0 mM (Fig. 5, *E*–*G*). Under conditions with 140 ng PheR, only PHE at concentrations below 0.1 mM retained a visible PheR–DNA complex. Among the tested analogs, PHE demonstrated the most pronounced derepression effect, indicating its preferential effector activity. Thus, these results indicate that PHE acts as an effector molecule of PheR, promoting its dissociation from the P_phnA1_ promoter.

### Phenanthrene acts as an effector that inhibits PheR binding to P_phnA1_*in vivo*

**Figure 6:**
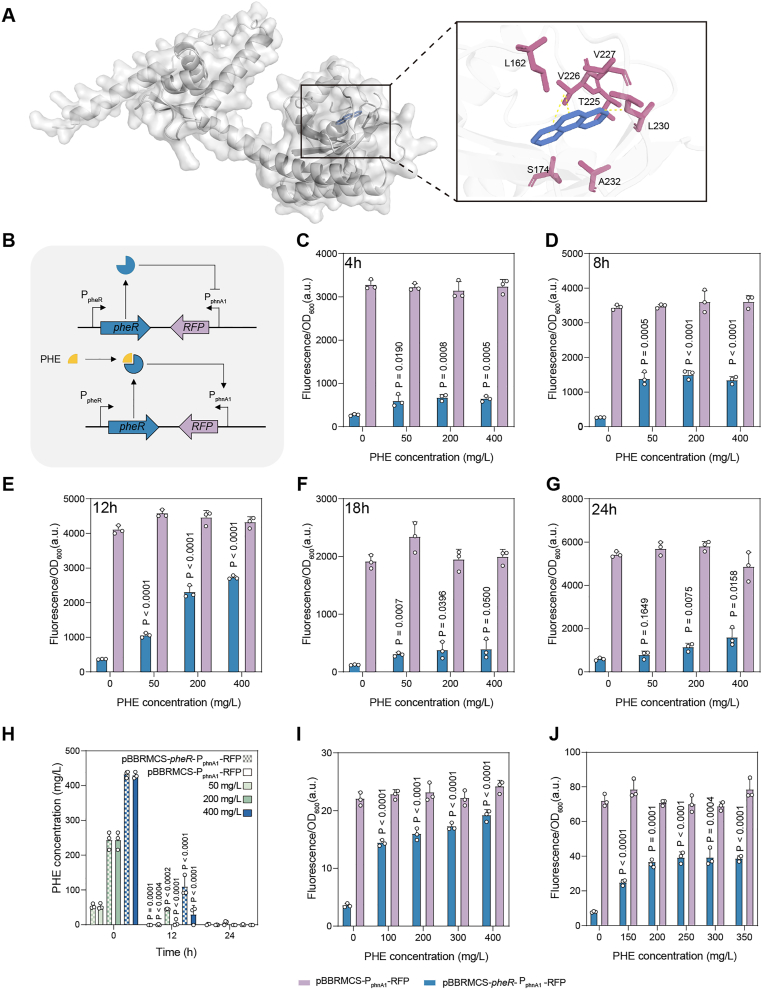
**Phenanthrene functions as an effector that inhibits PheR binding to P_phnA1_.***A*, molecular docking model of phenanthrene with PheR. *B*, schematic model illustrating phenanthrene-dependent derepression of P_phnA1_ in the absence of phenanthrene, PheR specifically binds to P_phnA1_ and represses downstream RFP expression. In the presence of phenanthrene, the effector weakens PheR-P_phnA1_ binding and partially relieves repression, leading to a phenanthrene-dependent increase in RFP expression. *C–G*, fluorescence responses of engineered strains SHPJ-2Δ*pheR*-pBBRMCS-P_phnA1_-RFP and SHPJ-2Δ*pheR*-pBBRMCS-*pheR*-P_phnA1_-RFP cultured in LB medium containing phenanthrene at final concentrations of 0, 50, 200, or 400 mg/L. RFP fluorescence was measured at 4 h (*C*), 8 h (*D*), 12 h (*E*), 18 h (*F*), and 24 h (*G*). *Blue*: SHPJ-2Δ*pheR*-pBBRMCS-*phe*R-P_phnA1_-RFP (PheR-dependent repression module). *Pink*: SHPJ-2Δ*pheR*-pBBRMCS-P_phnA1_-RFP (promoter-only control). Statistical analysis was performed only for SHPJ-2Δ*pheR*-pBBRMCS-*pheR*-P_phnA1_-RFP, with the 50, 200, and 400 mg/L groups each compared with the corresponding 0 mg/L group using an unpaired two-tailed Student’s *t* test. Exact *p* values are indicated in the figure. Data are presented as the mean ± SD (*n* = 3 biologically independent samples). *H*, phenanthrene degradation profiles of SHPJ-2Δ*pheR*-pBBRMCS-P_phnA1_-RFP and SHPJ-2Δ*pheR*-pBBRMCS-*pheR*-P_phnA1_-RFP grown in LB medium containing phenanthrene at initial working concentrations of 50, 200, and 400 mg/L. The indicated 50, 200, and 400 mg/L values represent the initial phenanthrene concentrations added to the culture medium. Statistical analysis was performed for both strains, with the 12 h and 24 h groups each compared with the corresponding 0 h group at each phenanthrene concentration using an unpaired two-tailed Student’s *t* test. Exact *p* values are indicated in the figure. *I–J*, RFP responses of pBBRMCS-P_phnA1_-RFP and pBBRMCS-*pheR*-P_phnA1_-RFP plasmids introduced into *E. coli* DH5α (*I*) and *Pseudomonasputida* sp. BGR4 (*J*), respectively. Fluorescence intensities were recorded after 12 h incubation in the presence of phenanthrene at final concentrations of 0, 100, 200, 300, and 400 mg/L. *I*, statistical analysis was performed only for DH5α-pBBRMCS-*pheR*-P_phnA1_-RFP, with the 100, 200, 300, and 400 mg/L groups each compared with the corresponding 0 mg/L group using an unpaired two-tailed Student’s *t* test. Exact *p* values are indicated in the figure. *J*, statistical analysis was performed only for BGR4-pBBRMCS-*pheR*-P_phnA__1_-RFP, with the 100, 200, 300, and 400 mg/L groups each compared with the corresponding 0 mg/L group using an unpaired two-tailed Student’s *t* test. Exact *p* values are indicated in the figure. Data are presented as the mean ± SD (*n* = 3 biologically independent samples).

Structural modeling also provided insight into the potential mechanism of PHE-dependent regulation. PHE fits into a hydrophobic pocket of PheR, surrounded by L162, T225, V226, V227, and L230, with nearby polar residues (S174 and A232) positioned to contribute additional contacts (Fig. 6*A*). This binding mode supports the role of PHE as a direct effector that weakens PheR-DNA binding affinity, as observed *in vitro*. In addition, residues predicted by PheR-PHE docking to interact with PHE were subjected to site-directed mutagenesis, and the resulting mutant proteins were analyzed by EMSA in the absence or presence of PHE (Figs. S6 and S7). These results support the predicted PHE-binding pocket and indicate that V226 and L230 are the key residues required for the PHE response of PheR. To this end, we constructed a transcriptional reporter in which a 300-bp upstream region of *phnA1* (P_phnA1_) was fused to an RFP gene (P_phnA1_–RFP). The native *pheR* regulatory module (P_pheR_–*pheR*) was cloned upstream of P_phnA1_–RFP to generate *pheR*–P_phnA1_–RFP. A J23119–RFP construct and a promoterless RFP plasmid served as positive and negative controls, respectively. These plasmids were introduced into the SHPJ-2Δ*pheR* background, yielding SHPJ-2Δ*pheR*-pBBRMCS-P_phnA1_–RFP and SHPJ-2Δ*pheR*-pBBRMCS-*pheR*–P_phnA1_–RFP strains (Fig. 6*B*).

Fluorescence measurements showed strong repression of RFP expression in the presence of *pheR*, confirming its repressor activity. Addition of phenanthrene rapidly relieved repression in a dose-dependent manner, with derepression detectable within 4 h and maximal at 12 h (Fig. 6, *C* and *E*). Derepression declined after 18 h (Fig. 6, *F* and *G*). Consistent with this temporal pattern, phenanthrene quantification showed complete degradation by 12 h in the engineered strain (Fig. 6*H*), indicating that reduced derepression at later times reflects effector depletion. In line with this interpretation, EMSA assays further showed that intermediate metabolites generated during phenanthrene degradation, including phthalic acid, salicylic acid and catechol, did not affect PheR binding to P_phnA1_ (Figs. S8–S10). These results demonstrate that phenanthrene functions as an effector *in vivo*, weakening PheR–P_phnA1_ binding and activating transcription of the phenanthrene catabolic operon. To assess portability, *pheR*–P_phnA1_–RFP and P_phnA1_–RFP were introduced into *Pseudomonas putida* BGR4 and *E. coli* DH5α. In both heterologous hosts, phenanthrene induced promoter derepression (Fig. 6, *I* and *J*), demonstrating that this regulatory circuit functions outside the native SHPJ-2 background. Together, these findings establish phenanthrene as the principal effector controlling PheR activity and underscore the potential of PheR as a portable PAH-responsive biosensing module.

### PheR functions as a negative regulator and global envelope metabolic regulator of phenanthrene degradation

**Figure 7:**
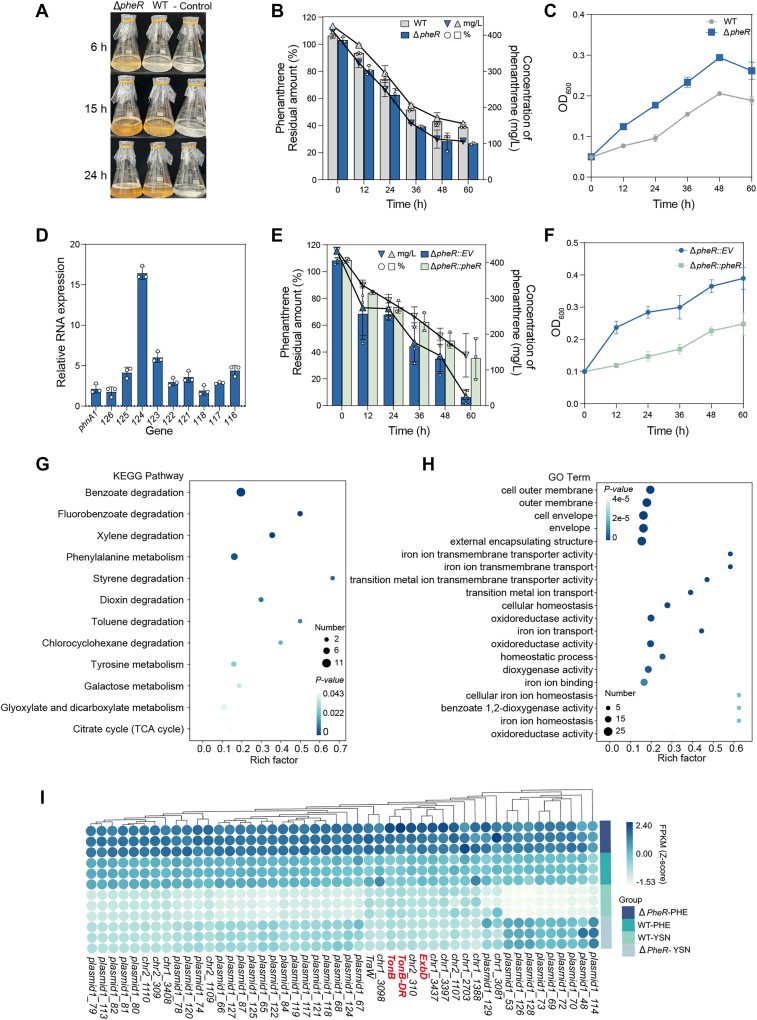
**PheR functions as a negative regulator and global envelope metabolic regulator of phenanthrene degradation.***A*, growth of wild-type SHPJ-2 and the SHPJ-2*ΔpheR* mutant in MSM with phenanthrene as the sole carbon source at 6, 15, and 24 h. *B*, phenanthrene degradation profiles of wild-type SHPJ-2 and the SHPJ-2*ΔpheR* mutant. *Blue* (SHPJ-2*ΔpheR*) and *gray* (WT) dots depict phenanthrene concentration over time (*left y-axis*), whereas bars of corresponding colors indicate residual phenanthrene levels (*right y-axis*). Data are presented as the mean ± SD (n = 3 biologically independent samples). *C*, growth curves of wild-type SHPJ-2 (*gray*) and the SHPJ-2*ΔpheR* mutant (*blue*) in MSM with phenanthrene as the sole carbon source. Data are presented as the mean ± SD (n = 3 biologically independent samples). *D*, RT-qPCR analysis of the expression of key phenanthrene degradation genes in wild-type SHPJ-2 and the SHPJ-2*ΔpheR* mutant. Data are shown as fold change relative to WT under PHE (WT set to 1.0 for each gene). Data are presented as the mean ± SD (n = 3 biologically independent samples). *E*, phenanthrene degradation assay of SHPJ-2*ΔpheR* strains complemented with either the empty vector (EV) (*blue*; negative control) or the *pheR*-expressing plasmid (*light green*). Dots represent phenanthrene concentration (*left y-axis*), and bars represent residual phenanthrene levels (*right y-axis*). Data are presented as the mean ± SD (n = 3 biologically independent samples). *F*, growth curves of the *ΔpheR* strain carrying either the empty vector (EV) or the *pheR* complementation plasmid in MSM with phenanthrene as the sole carbon source. Data are presented as the mean ± SD (n = 3 biologically independent samples). *G*, KEGG pathway enrichment analysis of differentially expressed genes (DEGs) between wild-type SHPJ-2 and the SHPJ-2Δ*pheR* mutant under phenanthrene-grown conditions. The x-axis shows the rich factor (number of DEGs annotated to a pathway total genes annotated to that pathway), and the y-axis lists enriched KEGG pathways. Dot size indicates the number of DEGs, while color intensity reflects enrichment significance. *H*, GO term enrichment analysis of DEGs between wild-type SHPJ-2 and the SHPJ-2Δ*pheR* mutant under phenanthrene-grown conditions. The x-axis shows the rich factor, and the y-axis displays enriched GO terms. Dot size corresponds to the number of DEGs per term, and color indicates statistical significance. *I*, heatmap showing expression patterns of phenanthrene-degradation–related DEGs in wild-type SHPJ-2 and the SHPJ-2Δ*pheR* mutant grown in MSM supplemented with either phenanthrene or sodium acetate as the sole carbon source. Expression values are shown as Z-score-normalized FPKM values, and genes were hierarchically clustered.

To define the regulatory role of *pheR*, a deletion mutant (SHPJ-*2*Δ*pheR*) was constructed and compared with the wild-type strain. While the wild type exhibited a pronounced lag phase of approximately 15 h before initiating PHE degradation, SHPJ-*2*Δ*pheR* showed little to no lag and degraded PHE substantially faster (Fig. 7*A*). Consistently, the mutant displayed a significantly higher growth rate than the wild type when PHE was provided as the sole carbon source (Fig. 7, *B* and *C*). RT-qPCR analysis further revealed that transcript levels of the PHE degradation genes were elevated 2- to 16-fold in SHPJ-2Δ*pheR* relative to the wild type (Fig. 7*D*), supporting a repressive function for PheR. Genetic complementation of SHPJ-*2*Δ*pheR* with *pheR* expressed from the broad-host-range plasmid pBBRMCS restored wild-type phenotypes in both PHE degradation and growth assays (Fig. 7, *E* and *F*). Collectively, these results demonstrate that PheR acts as a key negative regulator of phenanthrene degradation in *Sphingobium* sp. SHPJ-2.

RNA-Seq was performed on wild-type and Δ*pheR* strains harvested during exponential growth in MSM containing 400 mg/L phenanthrene. In total, 271 differentially expressed genes (DEGs) were identified, including 103 upregulated and 168 downregulated genes (|log_2_FC| > 1.5) (Fig. S11). KEGG enrichment analysis showed that PheR-regulated genes were predominantly associated with benzoate degradation, fluorobenzoate degradation, xylene degradation, and phenylalanine metabolism (Fig. 7*G*). Gene Ontology enrichment analysis further revealed overrepresentation of terms related to the cell outer membrane, cell envelope, and external encapsulating structures (Fig. 7*H*), suggesting that PheR-dependent transcriptional changes might be associated with envelope-related functions. Heatmap analysis of representative genes showed that the transcriptional profiles of phenanthrene-responsive genes were markedly altered in the Δ*pheR* mutant, including the entire *phnA1*-initiated phenanthrene degradation operon (Fig. 7*I*), in agreement with RT-qPCR results. Consistent with the underlying transcriptomic data, genes encoding TonB/ExbBD and multiple TonB-dependent receptors (TBDRs) were upregulated by approximately sevenfold in the Δ*pheR* mutant. Because the TonB-ExbBD complex couples the proton motive force to active transport across the outer membrane, this pattern suggests that PheR-dependent transcriptional changes are associated with functions related to outer-membrane transport and energy transduction. Together, these data support a model in which PheR coordinates phenanthrene catabolism with broader transcriptional responses linked to substrate uptake and envelope-associated functions during growth on phenanthrene.

### Phylogenetic distribution of PheR homologs in aromatic-compound–degrading bacteria

**Figure 8:**
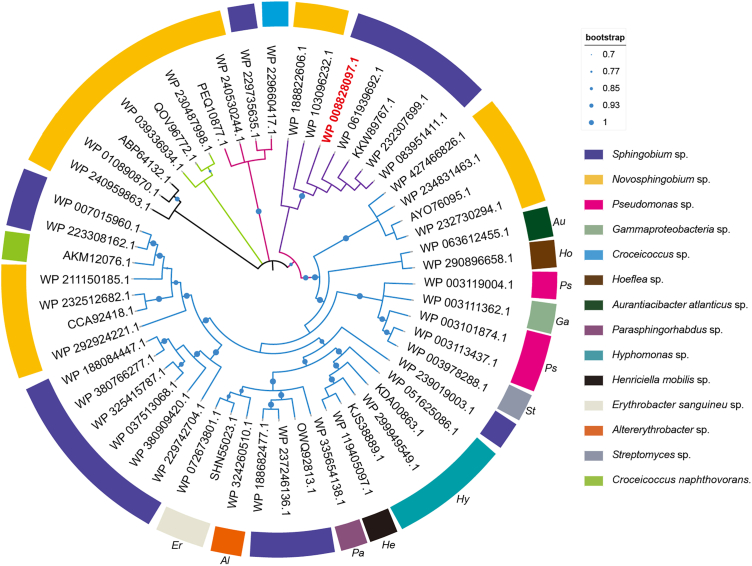
**Phylogenetic analysis of PheR homologs among aromatic-compound-degrading bacteria.** A maximum-likelihood phylogenetic tree was constructed based on amino acid sequences of PheR homologs. Colored blocks in the outer ring denote different bacterial genera. For taxa with similar colors, two-letter abbreviations corresponding to the first two letters of the genus names were added to improve visual discrimination. The five major clades are highlighted in *black*, *green*, *pink*, *purple*, and *blue*, respectively. Bootstrap support values are indicated at the nodes and were used to assess the reliability of the corresponding branches.

Bacteria from diverse genera within the Sphingomonadaceae and related Proteobacteria—including *Sphingobium*, *Novosphingobium*, *Croceicoccus*, *Pararhizobium*, and *Erythrobacter*—are frequently reported to degrade PAHs and other aromatic compounds. To place PheR (WP_008828097) from *Sphingobium* sp. SHPJ-2, in this ecological and evolutionary context, we performed a phylogenetic analysis of IclR-family homologs. The resulting tree resolved five major clades (Fig. 8). The black clade comprised exclusively *Novosphingobium* homologs, forming a well-supported, genus-specific lineage. A second *Novosphingobium*-only group formed the green clade, indicating independent diversification of two distinct IclR sublineages within this genus. The pink clade grouped homologs from *Novosphingobium*, *Erythrobacter*, and *Altererythrobacter*, suggesting a shared evolutionary origin among these Alphaproteobacteria and possible functional convergence in aromatic-rich environments.

The purple clade, which includes PheR, was composed primarily of *Novosphingobium* and *Sphingobium* sequences, defining the closest evolutionary neighborhood of PheR. The largest and most phylogenetically diverse clade encompassed homologs from *Hoeflea*, *Henriciella*, *Hyphomonas*, *Streptomyces*, and other taxa, and likely represents a broad environmental reservoir of IclR type regulators. Collectively, these results indicate that PheR is most closely associated with a sphingomonad-enriched subgroup, while related homologs are more broadly distributed among aromatic-compound-degrading bacteria.

## Discussion

Sphingomonads are frequently isolated from polycyclic aromatic hydrocarbon (PAH)–contaminated environments and are recognized as major degraders of representative PAHs such as phenanthrene (18, 19, 20). Despite this ecological prominence, the molecular logic by which these bacteria coordinate substrate recognition, transmembrane uptake, and initiation of catabolic pathways remains poorly defined (9). Classical studies have established that aromatic catabolic pathways are commonly organized as inducible operons governed by transcriptional regulators responsive to small aromatic acids, thereby avoiding the energetic cost of constitutive expression (21). Here, we identify PheR as a PHE-responsive IclR-family transcriptional repressor that directly controls transcriptional entry into the PHE catabolic program in *Sphingobium* sp. SHPJ-2. We found that the PHE degradation gene cluster is organized as a single, intact transcriptional unit. EMSA and DNase I footprinting assays demonstrated that PheR binds to the promoter of the gene cluster, P_phnA1_, and we further identified key amino acid residues critical for PheR binding affinity (R63, R73, and R78) as well as the DNA-binding motif (5′-GCAACG-3′). More importantly, both *in vitro* and *in vivo* experiments confirmed that phenanthrene addition disrupts PheR–P_phnA1_ binding, and that this effect is independent of intermediate metabolites generated during phenanthrene degradation. Together, our results support an effector-mediated derepression model: in the absence of phenanthrene, PheR binds to P_phnA1_ to repress transcription of the phenanthrene-degradation operon; upon phenanthrene exposure, the effector diminishes PheR–DNA binding, thereby relieving repression and enabling rapid operon activation and phenanthrene catabolism.

The IclR family was first characterized in the glyoxylate shunt of *E. coli* and is now known to play widespread roles in regulating the catabolism of benzoate, *p*-hydroxybenzoate, protocatechuate, and related aromatic acids (22, 23, 24, 25, 26). IclR proteins typically comprise a conserved N-terminal helix–turn–helix DNA-binding domain and a structurally flexible C-terminal α/β effector-binding domain, with subtle variations enabling selective recognition of diverse aromatic ligands (27). Genome-based analyses indicated that PheR is an IclR-family transcriptional regulator. However, multiple-sequence and structural comparisons with previously reported IclR-family proteins revealed no obvious sequence similarity to characterized members (Fig. S12 and Table. S5) (8). At the structural level, the closest matches were AttJ from *Agrobacterium tumefaciens* (accession no. Q8VPD8) and HmgR from *P. putida* (accession no. Q6EMJ0) (Fig. S13). AttJ is involved in the control of quorum-quenching activity linked to the stringent response and Ti plasmid conjugation, whereas HmgR functions as a repressor in the homogentisate catabolic pathway for phenylalanine and tyrosine degradation (28, 29). Notably, neither of these regulators has been implicated in regulatory mechanisms underlying polycyclic aromatic hydrocarbon degradation. Thus, PheR appears to represent a previously uncharacterized IclR-family regulator that has been recruited to control PAH catabolism, expanding the functional space of this family toward highly hydrophobic substrates such as PHE.

Different types of DNA-binding domains typically recognize distinct DNA motifs, and transcription factors within the same family often tend to target sites with comparable length, symmetry, and specificity (27). Notably, however, even proteins with very high amino-acid sequence identity (up to 60–70%) may evolve divergent preferences for different DNA motifs (30, 31). To date, binding motifs have been identified for a number of IclR-family regulators. Although it is generally thought that there is no single consensus sequence shared across the entire family, several characteristic motif types can be distinguished (24, 32, 33). One group comprises A/T-rich palindromic or palindrome-like motifs, such as those recognized by IclR (TGGAAATNATTTCCA), KdgR (RWWGAAACGNCGTTTCAKKA), AllR (KTTGGAAWAWTWTTCCAAC), and SsgR (TGAAAACTCACTCCT). Another group includes motifs featuring a GTNCG–N_5–6_–CGNAC consensus, represented by regulators such as HutR, CatR, PcaR, PcaU, PobR, HmgR, GenR, MhpR, NdgR/LtbR, NpdR, OphR, and TphR. During transcription factor–DNA recognition, conservation is not uniform across positions within a motif; instead, it is often closely associated with the number of contacts a given base makes with the protein in the complex. In general, base pairs that participate in more protein–DNA contacts are more likely to be conserved during evolution, because these amino-acid–base interactions can stabilize the protein–DNA complex, rendering substitutions at these positions functionally unfavorable. In this study, mutational analysis within the PheR-binding site established 5′-GCAACG-3′ as an essential minimal determinant for PheR binding to P_phnA1_. This result indicates that PheR recognition depends strongly on a short core sequence within the operator region. This finding is consistent with the comparative framework proposed for the IclR family. Previous analyses have suggested that IclR-family palindromic-binding sites can be broadly classified into two major consensus types: GKTYCRYW3–4RYGRAMC (group 1) and TGRAACAN1–2TGTTYCA (group 2) (8). The GCAACG element identified here can be viewed as a local sequence feature within the broader IclR binding-site repertoire, highlighting that key bases contributing to recognition can be identified experimentally even in motif-diverse families. Nevertheless, although 5′-GCAACG-3′ represents an essential core determinant for PheR recognition, the broader 44-bp DNase I-protected region observed in our footprinting assay suggests that this motif alone may not fully define the entire PheR-bound region. Together with the Hill coefficient (nH = 1.95), these data are consistent with the possibility that PheR binds P_phnA1_ cooperatively. In addition, sequences flanking the core motif may also contribute to stable complex formation.

Empirical rules of protein–DNA recognition generally reflect the chemical and physical properties of amino-acid residues and base pairs, including partial-charge interactions, hydrogen-bond donor/acceptor complementarity, and side-chain flexibility and spatial accessibility (30). We identified three key positions that markedly affect DNA binding—R63, R73, and R78—all of which are arginine residues. The guanidinium group of arginine is positively charged under physiological conditions and provides multiple hydrogen-bond donors, together with a relatively long side chain; thus, arginine frequently serves as a critical “anchoring” residue in protein–DNA interactions. It can stabilize the DNA phosphate backbone *via* salt bridges/electrostatic interactions while also helping to impose geometric constraints and positioning on the DNA. Substituting these residues with alanine simultaneously removes the positive charge, the capacity to form an extensive hydrogen-bonding network, and the side-chain length, thereby substantially weakening the formation and stability of the protein–DNA complex. This observation is consistent with comparative analyses of the IclR family. Previous studies have suggested that DNA contacts made by IclR-family transcription factors are largely concentrated in the recognition helix of the HTH DNA-binding domain (typically corresponding to α3), which is often enriched in charged residues and hydrogen-bond donors. Given the well-established physicochemical properties of arginine and its common roles at protein–DNA interfaces, R63, R73, and R78 may jointly contribute to both binding stability and the geometry of sequence recognition: arginine can form salt bridges with the DNA phosphate backbone to increase overall affinity and complex lifetime, and it may also establish specific hydrogen-bonding networks with the edge atoms of G/C base pairs in the major groove, thereby influencing base preference and positioning accuracy. In addition, comparative genomics studies have proposed that, within IclR-family motifs, base positions that engage in more direct contact with the transcription factor tend to be more conserved; correspondingly, protein residues responsible for key contacts are also more likely to be under stronger selective pressure. Accordingly, the functional importance of R63/R73/R78 likely stems from their participation in multiple contacts and cooperative anchoring at the protein–DNA interface, such that perturbations at these positions can substantially impair formation of the PheR–DNA complex. Because the residues critical for DNA binding (R63/R73/R78) do not overlap with those predicted by structural modeling to line the PHE-binding pocket (*e.g.*, L162, T225, V226, V227, and L230), we consider it unlikely that PHE regulation can be explained solely by direct competition at the DNA-binding interface. This interpretation is further supported by mutational analysis of the predicted PHE-binding pocket, in which substitution of V226 or L230 abolished the response of PheR to PHE without disrupting basal DNA binding, whereas other substitutions affected DNA binding to different extents. Together, these results support a model in which PHE binding modulates the DNA-binding activity of PheR through an allosteric mechanism rather than through direct competition at the DNA-binding interface.

Effector chemistry—especially hydrophobicity and hydrogen-bonding capacity—strongly shapes binding-pocket composition. For phenanthrene, a largely nonpolar PAH with few heteroatoms, binding is typically dominated by the hydrophobic effect and van der Waals packing, so the pocket core is enriched in aliphatic hydrophobic residues (*e.g.*, Leu/Val/Ile/Met/Ala), while polar residues tend to sit at the rim/entrance to aid positioning *via* weak contacts or conformational gating. This is consistent with TetR regulators, whose ligand-recognition domains often use hydrophobic cavities; in AlkX, structural data indicate that hydrophobic pocket residues provide the major contacts to the inducer (34, 35). In contrast, effectors with carboxylate/phenolic hydroxyl groups (*e.g.*, salicylate) are frequently stabilized in MarR repressors by defined hydrogen-bond/electrostatic networks involving polar/charged residues, reflecting higher polar participation (36). BenM further supports this principle by using distinct sites for a polar dicarboxylate *versus* a more hydrophobic aromatic ligand (benzoate), with benzoate binding in an adjacent hydrophobic region (37). In our docking model, phenanthrene occupies a hydrophobic pocket of PheR lined by L162, V226, and L230, with nearby T225 and S174 contributing additional contacts—consistent with a hydrophobic core providing affinity and sparse polar residues supporting orientation rather than strong hydrogen-bond locking (38).

Transcription factor–based whole-cell biosensors are widely used for environmental monitoring, substrate screening, and metabolic flux control. Aromatic-responsive regulators such as XylR/XylS, CatM/BenM, and LysR have been successfully repurposed as biosensors for phenols, toluene, and hydroxybenzoates, with performance further enhanced through directed evolution, domain engineering, and machine learning–guided design (39, 40, 41). Identification of PheR extends the repertoire of engineerable regulatory elements to highly hydrophobic PAHs. PheR displays several favorable properties, including phenanthrene-dependent derepression of the *phnA1* operon, a large ON/OFF dynamic range characteristic of repressors, functional responsiveness across host backgrounds, and a native *phnA1*–operator architecture amenable to modular refactoring. Building on rational design strategies established for AraC- and LysR-type regulators (42, 43, 44), targeted mutagenesis or semi-rational library screening could expand PheR’s sensing range to other PAHs such as anthracene and pyrene or further sharpen its specificity for phenanthrene (45).

Beyond local repression of *phnA1*, comparative transcriptomics reveal that PheR operates at a regulatory tier well above a single operon. When phenanthrene is the sole carbon source, deletion of *pheR* not only strongly upregulates genes involved in benzoate, fluorobenzoate, and xylene degradation but also robustly induces *tonB*, *exbB*, *exbD*, and multiple TonB-dependent receptors (TBDRs) (46, 47, 48). GO enrichment analysis further shows that differentially expressed genes are highly enriched for functions associated with the outer membrane, cell envelope, and external encapsulating structures. This expression pattern closely mirrors TonB–TBDR–mediated lignin-derived aromatic uptake systems described in *Sphingobium* sp. SYK-6 and aligns with RB-TnSeq studies identifying the TonB system as a central determinant of aromatic compound utilization (49, 50). The ∼7-fold induction of TonB/ExbBD and multiple TBDRs in the Δ*pheR* mutant suggests that, in the wild type, PheR acts as a global regulator that concurrently constrains substrate influx and downstream metabolic flux under PAH-limiting conditions. Such coordinated repression likely prevents excessive substrate entry that could compromise membrane integrity or redox balance. Upon phenanthrene recognition, this repression is synchronously relieved, enabling coordinated activation of PAH uptake, degradation, and envelope remodeling. In *Sphingobium* sp. SHPJ-2, PheR functions as the central hub of this network: In the absence of phenanthrene, it maintains a tightly repressed state by simultaneously inhibiting the *phnA1B1* catabolic operon and the TonB/TBDR uptake module; upon substrate availability, derepression is synchronously triggered, rapidly elevating PAH uptake capacity, catabolic flux, and envelope adaptation.

In summary, our findings support a regulatory model in which sphingomonads adapted to PAH-rich environments rely not only on local operon-level switches, but also on higher-order networks assembled by IclR-family transcription factors to integrate substrate sensing, outer-membrane energy transduction, and envelope homeostasis. This architecture provides a mechanistic explanation for the combination of high degradation efficiency and environmental resilience characteristic of sphingomonads in PAH-contaminated ecosystems and establishes a strong foundation for developing PheR-based PAH-responsive biosensors and programmable regulatory modules for advanced bioremediation applications.

## Experimental procedures

### Chemicals, bacteria, plasmids, and growth conditions

All chemicals used in this study were of analytical grade and of the highest commercially available purity (≥99%). Phenanthrene and standards of metabolic intermediates (*e.g.*, 1-hydroxy-2-naphthoic acid and salicylic acid) were purchased from J&K Scientific (Beijing, China). Enzymes used for molecular biology were purchased from Vazyme Biotech (Nanjing, China), and other routine reagents were obtained from Sangon Biotech (Shanghai, China). Water-insoluble substrates were dissolved in N,N-dimethylformamide (DMF) as stock solutions and added to media at the indicated final concentrations; vehicle-control cultures received the same final DMF concentration as substrate-treated cultures. Specifically, phenanthrene was prepared as a 400 mg/ml stock in DMF and added to cultures at 50 μl per 50 ml medium (final, 400 mg/L), and control cultures received the same volume of DMF (final DMF, 0.1% [v/v]). For culture-based assays, phenanthrene was used at 400 mg/L (2.24 mM) unless otherwise indicated. Mineral salts medium (MSM) (per liter) contained 3.7*g* KH_2_PO_4_, 5.2*g* K_2_HPO_4_·3H_2_O, 2.0*g* NH_4_Cl, 1.0*g* Na_2_SO_4_, 0.1*g* MgSO_4_, and 1 ml trace metal solution. Unless otherwise indicated, SHPJ-2 was cultured in MSM supplemented with the indicated carbon source(s) at 30 °C. *E. coli* strains used for recombinant DNA procedures were cultivated at 37 °C in Luria–Bertani (LB) medium (1% tryptone, 0.5% yeast extract, and 1% NaCl) with antibiotics as appropriate. Oligonucleotide primers are listed in Table S1 and bacterial strains are listed in Table S2.

### RNA extraction, reverse-transcription PCR (RT-PCR), and real-time quantitative PCR (RT-qPCR)

*Sphingobium* sp. SHPJ-2 is able to utilize phenanthrene (PHE) as the sole carbon source in mineral salts medium (MSM). To determine transcriptional responses of PHE-degradation genes, SHPJ-2 was inoculated into MSM supplemented with 10 g/L sodium acetate (YSN) (control) or 400 mg/L PHE (treatment) as the sole carbon source. Phenanthrene was prepared as a 400 mg/ml stock in DMF and added to cultures at 50 μl per 50 ml medium (final, 400 mg/L); control cultures received the same volume of DMF (final DMF, 0.1% [v/v]). Cultures were incubated at 30 °C and cells were harvested at the mid-to-late exponential phase. Total RNA was extracted using the TransZol Up Plus RNA Kit (TransGen Biotech) according to the manufacturer’s instructions. Reverse transcription was performed using a TransGen Biotech cDNA synthesis kit following the manufacturer’s protocol.

### Determination of the transcription start sites of the phenanthrene degradation gene cluster

The transcription start sites (TSSs) of the phenanthrene degradation gene cluster were determined by 5′-rapid amplification of cDNA ends (5′ RACE) using the HiScript-TS 5′/3′ RACE kit (Vazyme Biotech) according to the manufacturer’s instructions. The total RNA used was extracted as described above. The primers used in this experiment are listed in Table S1. The final PCR products were purified with a SteadyPure agarose gel DNA purification kit (Accurate Biotechnology Co, Ltd) and then ligated into pCE2 TA/Blunt-Zero vector using the Blunt-Zero Cloning kit (TransGen biotech) for sequencing. The promoter regions of the two transcriptional units were predicted by Softberry (http://softberry.com) and BDGP (https://www.fruitfly.org/seq_tools/promoter.html).

### Genetic disruption and complementation

Two DNA fragments corresponding to the flanking regions of the *phnA1* and *pheR* gene 1000 bp upstream and downstream were amplified using the primer pairs PEX18-3137L-A-F/R, PEX18-3137L-C-F/R, iclR-A-F/R and iclR-B-F/R, respectively. The resulting fragment was cloned into pEX18Tc, yielding pEX-*phnA1* and pEX-*pheR*, which were electroporated into strain SHPJ-2. Single-crossover mutants were screened on LB agar containing 8.5 μg mL^−1^ of tetracycline. After verification, a single-crossover mutant was cultured until an OD_600_ of approximately 0.6, and double-crossover mutants were selected on LB agar containing 8.5 μg mL^−1^ of tetracycline and 15% sucrose. Both single and double crossover mutants were verified by PCR and DNA sequencing. The double-crossover mutant was designated as SHPJ-2Δ*phnA1* and SHPJ-2Δ*pheR*. The *pheR* gene amplified with the primer pair pheR-F/pheR-R was inserted into pBBRMCS-5 to generate pBBRMCS-*pheR*, which was electroporated into strains SHPJ-2Δ*pheR* to obtain the *pheR* complementary strain SHPJ-2Δ*pheR*-pBBRMCS-*pheR*.

### Characterization of PHE degradation and growth of strains

All PHE degraders (SHPJ-2, SHPJ-2*ΔpheR*, SHPJ-2*ΔphnA1* and SHPJ-2Δ*pheR*-pBBRMCS-*pheR*) were grown in MSM containing 400 mg/L PHE. To study the effect of *pheR* on the degradation performance of strain SHPJ-2, 5% (v/v) seed broth was inoculated into MSM containing 400 mg/L PHE at pH 7 and incubated at 30 °C and 200 rpm. The degradation ability and growth curve of the strain were determined at 0 h, 12 h, 24 h, 36 h, 48 h, and 60 h, respectively. Cell growth was monitored by measuring OD600. For PHE quantification, the entire culture was extracted with an equal volume of ethyl acetate (1:1, v/v) at 30 °C and 200 rpm for 40 min. The organic phase was then collected and centrifuged at 12,000 rpm for 5 min. The supernatant was analyzed by HPLC using an Agilent Eclipse XDB C18 reverse-phase column (5 μm, 4.6 × 150 mm) and an Agilent G1315D DAD detector. The mobile phase consisted of methanol and 0.1% formic acid (80:20, v/v), delivered at 0.8 ml/min. The injection volume was 5 μl, the column temperature was 30 °C, the detection wavelength was 254 nm, and the run time was 15 min. The remaining PHE concentration was calculated according to peak area based on an HPLC standard curve prepared with authentic phenanthrene standards. Data were analyzed using GraphPad Prism. Unless otherwise indicated, all experiments were performed with at least three independent biological replicates.

### Reporter plasmid construction

To identify the effectors of PheR, a 300-bp DNA fragment upstream of the TSS of *phnA1* (P_phnA1_) was amplified by PCR and ligated into pBBRMCS-5, which carries the RFP reporter gene to generate the reporter plasmid pBBRMCS-P_phnA1_-RFP. In addition, the native regulatory region containing *pheR* and its promoter was inserted upstream of the P_phnA1_–RFP fusion to generate the pBBRMCS-*pheR*-P_phnA1_-RFP reporter construct. The resulting plasmids, pBBRMCS-P_phnA1_–RFP and pBBRMCS-*pheR*-P_phnA1_-RFP, were electroporated into strain SHPJ-2Δ*pheR* and *P. putida* BGR4, respectively, for fluorescence assays. For the reporter assay, recombinant strains were cultured in medium supplemented with different amounts of phenanthrene stock solution (400 mg/ml) to obtain final phenanthrene concentrations of 0, 50, 200, and 400 mg/L. Red fluorescence was measured at 4, 8, 12, 18, and 24 h using a Tecan Spark fluorometer (excitation, 583 ± 10 nm; emission, 607 ± 10 nm; gain = 120). Fluorescence data were analyzed using GraphPad Prism. The background fluorescence and OD_600_ values were determined using blank wells containing fresh LB medium and subtracted from those of the experimental groups. Unless otherwise indicated, all experiments were performed with at least three independent biological replicates.

### Heterologous expression and purification of protein

The gene *pheR* was amplified from the genomic DNA of strain SHPJ-2 using the primer pair pheR-F/pheR-R. *pheR* was then cloned into pET-28a (+) to yield pET-*pheR* using the ClonExpress II One-step Cloning Kit (Vazyme, Vazyme Biotech Co, Ltd). The resulting plasmid was subsequently transformed into *E. coli* BL21(DE3). The cells were cultured in LB medium at 37 °C to an OD600 of 0.6 and then induced with 0.05 mM isopropyl-*β*-D-thiogalactopyranoside at 16 °C for an additional 16 h. Cell lysate was obtained by sonication, and the C-terminal His6-tagged protein was purified using a 1 cm^3^ Ni^2+^-charged resin column (HiTrap Talon crude; GE Healthcare Life Sciences). Protein concentration was determined by the Bradford method. The molecular mass of the purified enzymes was estimated by SDS-PAGE. The multimeric state of the native PheR was determined by gel filtration chromatography. PheR was loaded onto a Superdex 200 Increase 10/300 Gl Column (GE Healthcare) using 20 mM Tris-HCl buffer (pH 8.5, with 300 mM NaCl) as mobile phase (0.4 ml min−1) at 25 °C.

### Electrophoretic mobility shift assay

EMSA was performed to assess binding of PheR to the *phnA1* promoter/operator region. A 150-bp DNA fragment encompassing the *phnA1* promoter (P_phnA1_) was amplified by PCR using primer pair 127-F1/R1. A 100-bp fragment within the *phnA2* coding region was amplified and using primer pair control-F/R and served as a nonspecific negative-control probe (Table S1). For each binding reaction (total volume, 10 μl), ∼40 ng DNA probe was incubated with purified PheR at 0, 17.5, 35, 70, 140, or 280 ng in binding buffer containing 100 mM Tris-HCl (pH 8.5), 50 mM KCl, 5% (v/v) glycerol, 0.25 mM EDTA, and 1 mM dithiothreitol (DTT). Reactions were incubated at 30 °C for 60 min. Where indicated, phenanthrene was added to a final concentration of 0.5 mM from a DMF stock (prepared as 400 mg/ml in DMF and diluted to a 5.0 mM working solution in DMF); vehicle controls received the same volume of DMF. The final DMF concentration was 10% (v/v) in all reactions. Samples were resolved on 5% (w/v) native polyacrylamide gels in 0.5 × Tris–glycine–EDTA buffer. Phenanthrene was prepared as a stock solution in DMF and added to EMSA reactions at the indicated final concentrations; vehicle control reactions received the same volume of DMF, and DMF alone did not affect PheR–DNA binding under our assay conditions. DNA and DNA–protein complexes were visualized by staining with SYBR Gold nucleic acid gel stain (Invitrogen, S11494) and imaged under UV illumination. Band intensities of shifted and free DNA were quantified by grayscale analysis using Adobe Photoshop CC 2019.

### DNase I footprinting assay

DNase I footprinting was performed to map the PheR-protected region within the *phnA1* promoter. A 300-bp *phnA1* promoter fragment was cloned into pMD19-T to generate plasmid R-1. For preparation of fluorescent probes, the promoter insert was PCR amplified from plasmid R-1 using 2 × TOLO HiFi DNA polymerase premix (TOLO Biotech, Shanghai, China) with primers M13F (5′ end labeled with 5(6)-carboxyfluorescein [FAM]) and M13R, yielding FAM-labeled DNA probes. PCR products were purified using the Wizard SV Gel and PCR Clean-Up System (Promega) and quantified with a NanoDrop 2000C spectrophotometer (Thermo Fisher Scientific). For each footprinting reaction, 300 ng of FAM-labeled probe was incubated with purified PheR in a total volume of 40 μl. Reactions were performed with 0 μg PheR (no-protein control) or 10 μg PheR (protection condition) and incubated at 25 °C for 30 min. DNase I digestion was initiated by adding 100 nmol freshly prepared CaCl_2_ to a 10-μl DNase I solution containing ∼0.015 U DNase I (Promega, USA), followed by incubation at 37 °C for 1 min. Reactions were stopped by addition of 140 μl DNase I stop solution (200 mM unbuffered sodium acetate, 30 mM EDTA, 0.15% SDS). Samples were extracted with phenol/chloroform and precipitated with ethanol. Pellets were dissolved in 30 μl Milli-Q water. DNA ladder preparation, electrophoresis, and data analysis were performed as previously described (51), except that the GeneScan LIZ600 size standard (Applied Biosystems) was used.

### *RNA-seq* and data analysis

To study the expression of PAH degradation-related genes in strain SHPJ-2 and SHPJ-2*ΔpheR*, bacteria were cultured with MSM containing 200 mg/L PHE or 1% sodium acetate (w/v) and collected at the logarithmic phase. *RNA-seq* was conducted by Shanghai Personalbio Technology Co Ltd, China. Total RNA was extracted using TRIzol reagent (R0016, Beyotime, China), and genomic DNA was removed with DNase I (TaKara, Japan). Ribosomal RNA (16S and 23S rRNA) was depleted from the total RNA using a Ribo-Zero Magnetic kit (Epicenter Biotechnologies, WI, USA). mRNA was subsequently fragmented (∼200 bp) using a fragmentation buffer. Complementary DNA (cDNA) synthesis was performed through reverse transcription using the SuperScript double-stranded cDNA synthesis kit (Invitrogen) with random hexamer primers (Illumina). During second-strand cDNA synthesis, deoxyuridine triphosphate (dUTP) was incorporated in place of deoxythymidine triphosphate (dTTP) to generate blunt-ended cDNA fragments. These double-stranded cDNA fragments then underwent end repair, phosphorylation, 3′ adenylation, and adapter ligation. The second-strand cDNA containing dUTP was selectively degraded using uracil-N-glycosylase (UNG) enzyme. Following degradation, cDNA fragments were separated on a 2% agarose gel, and DNA fragments of approximately 200 bp were extracted for cDNA library construction. PCR amplification of the cDNA libraries was performed using Phusion DNA polymerase (NEB) for 15 cycles. The libraries were quantified using a microfluorometer (TBS-380, TurnerBioSystems) and sequenced on an Illumina HiSeq × Ten platform using paired-end sequencing. Bioinformatics analyses were conducted using the cloud-based platform of Shanghai Personalbio Technology Co Ltd, based on sequencing data generated by the Illumina platform. Differentially expressed genes (DEGs) among the samples were identified based on quantitative gene expression analysis. The DEGs were further subjected to Gene Ontology (GO) enrichment analysis and Kyoto Encyclopedia of Genes and Genomes (KEGG) pathway enrichment analysis. In addition, volcano plots and heatmaps were generated to visualize differential gene expression patterns among samples. The raw data have been deposited at the National Center for Biotechnology Information (NCBI) under the BioProject accession number PRJNA1427403.

### Isothermal titration calorimetry

DNA fragments and P_phnA1_ were prepared in PBS buffer just before the ITC assay. All DNA fragments, including the inverted repeat regulatory sequence (5′- GTCGATTCGTGCTATACATGCAACGCGATGCACCATATGCAAAACCTACC-3′), were 50 bp in length. All samples were degassed with vacuum aspiration for 10 min before analysis with an ITC200 instrument (MicroCal). The reaction cell was filled with 64 μM PheR solution, and the titration was performed with an initial 0.4-μl injection of 100 μM a given DNA fragment, followed by 19 injections of 2 μl of the DNA fragment spaced at 2-min intervals. Titrating buffer was used as the control. The binding stoichiometry (N) value and the equilibrium dissociation constant (K_*d*_) were calculated using Origin 7.0 software.

### Biolayer interferometry (BLI)

BLI assays were performed using a ForteBio Octet RED 96 system (FortéBio). All binding studies were carried out at 30 °C. Streptavidin (SA) sensors were loaded with biotinylated DNA (100 nM), in a buffer containing 20 mM Tris-HCl, pH 8.5, 300 mM NaCl, and 0.02% Tween 20 (v/v). After reaching baseline in the same buffer, association and dissociation were carried out with purified PheR and buffer, respectively. Steady-state binding responses were determined by the overall response (nm) on each sensor, and the data were analysed using the system software of Octet RED 96. To determine the Hill coefficient, PheR (100–10000 nM) was used to interact with the immobilized fragment. As previously described, the fractional saturation, Θ = Req/Rmax, was calculated. For each concentration, Req is the signal at equilibrium. Rmax represents the maximum binding response when all the immobilized DNA was bound. Log [Θ/(1 − Θ)] as a function of log [PheR] was used to fit with the Hill equation, log [Θ/(1 − Θ)] = KH + nH log [PheR]. KH and nH represent the Hill constant and Hill coefficient respectively.

### Molecular docking analysis

To evaluate the potential binding mode between candidate compounds and the target protein, molecular docking analysis was performed. The three-dimensional structure of PheR was obtained from the RCSB Protein Data Bank and prepared using AutoDock Tools by removing water molecules, adding polar hydrogen atoms, and assigning Gasteiger charges. The processed protein structure was then saved in PDBQT format. The candidate ligand PHE was converted into a three-dimensional structure and subjected to energy minimization before docking. Docking calculations were carried out using AutoDock Vina. The docking grid box was defined based on the predicted binding pocket to ensure coverage of the potential ligand-binding region. Default parameters were used for conformational sampling, and multiple binding poses were generated. The docking results were ranked according to the Vina scoring function, with lower binding energies indicating stronger predicted binding affinity. The conformation with a favorable binding energy and a reasonable binding pose was selected for subsequent analysis and visualization.

### Structure-based phylogenetic tree analysis

A structure-based computational strategy was used to construct a structure-based phylogenetic tree, with PhnA1 used as the target reference protein. The three-dimensional structure of PhnA1 was predicted using ESMFold, and structural similarity searches were performed using Foldseek. TM-score was used as the primary metric to quantify global structural similarity. Transcriptomic log_2_FC and Benjamini-Hochberg-adjusted *p* values (*P-*adj) were incorporated as annotation tracks. Only proteins with *P-*adj < 0.1 were retained for visualization in Fig. S2. Based on pairwise TM-score comparisons, proteins showing relatively high structural similarity to PhnA1 were identified and used to construct the structure-based phylogenetic tree. The complete TM-score, log_2_FC, and *P-*adj values used for plotting are provided in Table S4.

### Statistical analysis

A Student’s *t* test was performed to identify significant differences using GraphPad Prism. Statistical significance was determined at the *p* < 0.05 level. Three independent biological replicates were conducted for each sample. Data are shown as the mean ± standard deviations of triplicate experiments.

### Data and materials availability

The raw data have been deposited at the National Center for Biotechnology Information (NCBI) under the BioProject accession number PRJNA1427403.

## Supporting information

This article contains supporting information.

## Conflict of interest

The authors declare that they have no conflicts of interest with the contents of this article.
