# Supplementary material for: The transcriptional repressor PheR regulates uptake and catabolism of phenanthrene in Sphingobium sp. SHPJ-2
Source: J Biol Chem. 2026 May 12;302(7):113140. doi: 10.1016/j.jbc.2026.113140 (PMC13273656; doi:10.1016/j.jbc.2026.113140)
Supplement: Supplementary Material [file mmc1.docx]

**Supplementary Information for**

**The transcriptional repressor PheR regulates uptake and catabolism of phenanthrene in *Sphingobium* sp. SHPJ-2**

Wanjing Wu, Wanxin Chen, Yuan Yang, Xu Qiu, Ping Xu, Hongzhi Tang*, Weiwei Wang*

State Key Laboratory of Microbial Metabolism, and School of Life Sciences & Biotechnology, Shanghai Jiao Tong University, Shanghai, 200240, P.R. China

*Corresponding author: W. W. Wang or H. Z. Tang

Email: [oudigouzai@sjtu.edu.cn](mailto:oudigouzai@sjtu.edu.cn) or tanghongzhi@sjtu.edu.cn

Tel: +86-21-34204066.

**The supplementary information file includes:**

Supplementary figure S1-S13

Supplementary table S1-S5


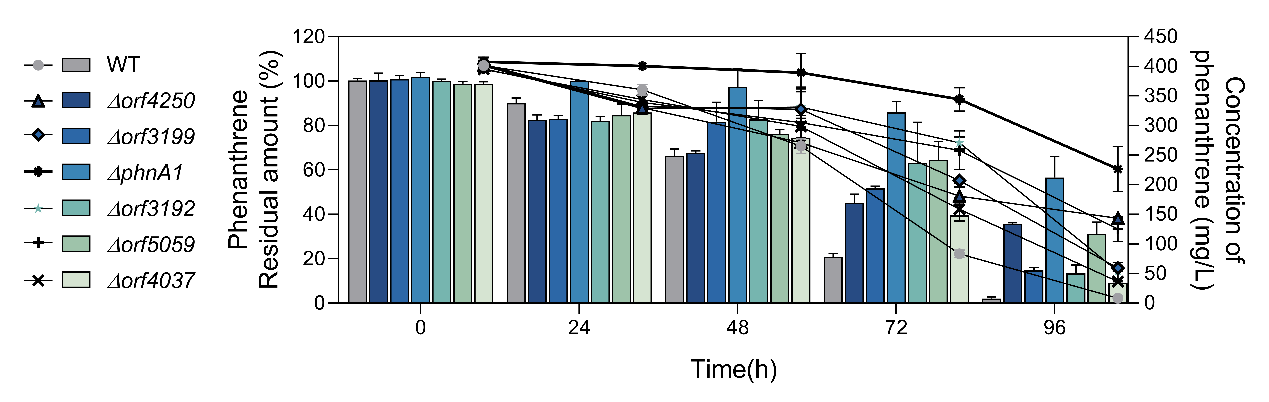


**Fig. S1. Phenanthrene degradation efficiency and residual phenanthrene concentration of wild-type *Sphingobium* sp. SHPJ-2 and single-gene deletion mutants targeting candidate PAH-oxidation enzymes.**Phenanthrene degradation efficiency is shown on the left y-axis (symbols), and the corresponding residual phenanthrene concentration is shown on the right y-axis (bars) over the indicated time course. Gray symbols/bars represent the wild-type strain, and colored symbols/bars represent the corresponding mutant strain. Mutants include Δ*phnA1* (locus tag plasmid1_127) and additional deletions of candidate oxygenases: Δ*orf4250* (predicted ring-hydroxylating dioxygenase, RHD) and Δ*orf3199,* Δ*orf3192,* Δ*orf5059,* Δ*orf4037* (predicted cytochrome P450 monooxygenases). Cultures were grown in MSM with phenanthrene as the sole carbon source (final 400 mg/L; DMF vehicle matched across conditions; see Methods).


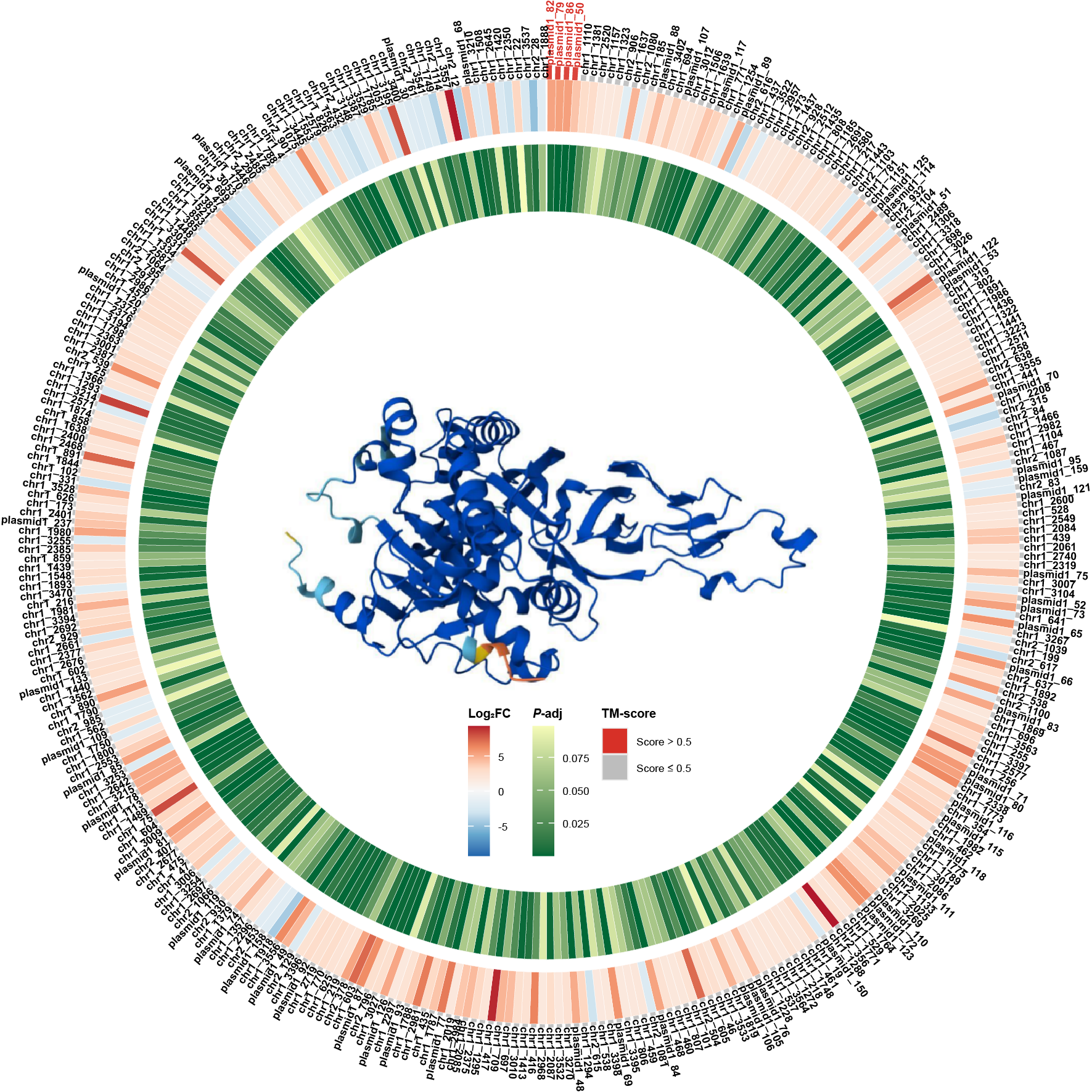


**Fig. S2. Structure-based phylogenetic tree of significantly responsive SHPJ-2 proteins using PhnA1 as the reference protein.** Proteins retained after transcriptomic filtering (*P*-adj < 0.1) were structurally compared with PhnA1 (plasmid1_127). The inner heatmap shows the log_2_FoldChange (log_2_FC) in transcript abundance (red, upregulated; blue, downregulated), the middle heatmap shows the Benjamini-Hochberg-adjusted *P* value (*P*-adj), and the outer track shows the TM-score, indicating structural similarity to PhnA1. The proteins showing the highest structural similarity to PhnA1, including plasmid1_50, plasmid1_82, plasmid1_86, and plasmid1_79, are predominantly annotated as Rieske 2Fe-2S-containing aromatic oxygenase/dioxygenase proteins. The complete TM-score, log_2_FC, and *P*-adj values for all proteins shown in this figure are provided in Table. S4.


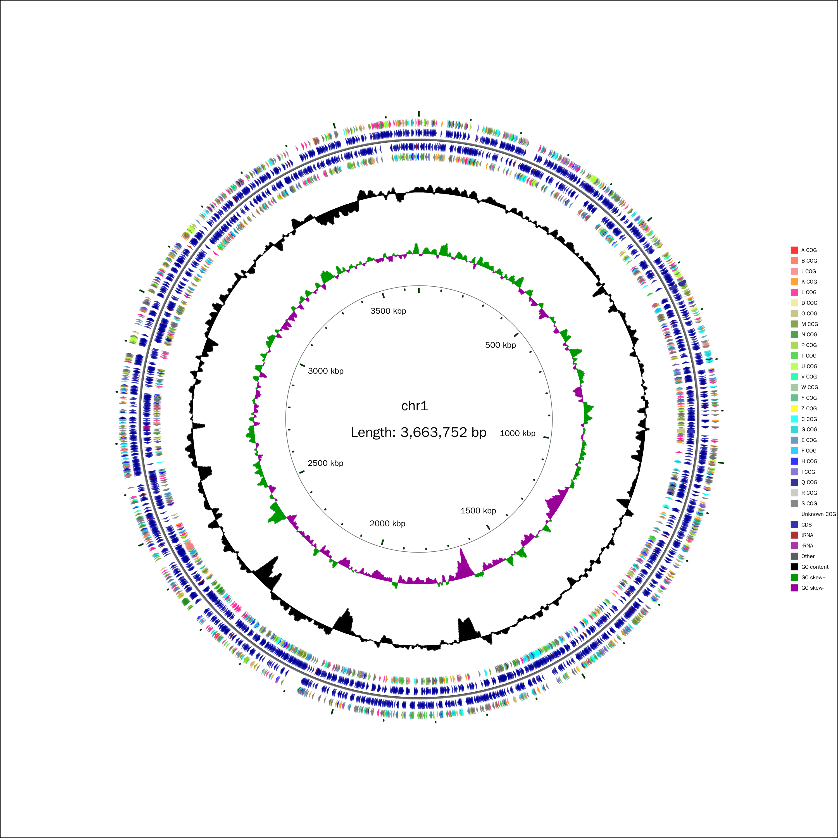

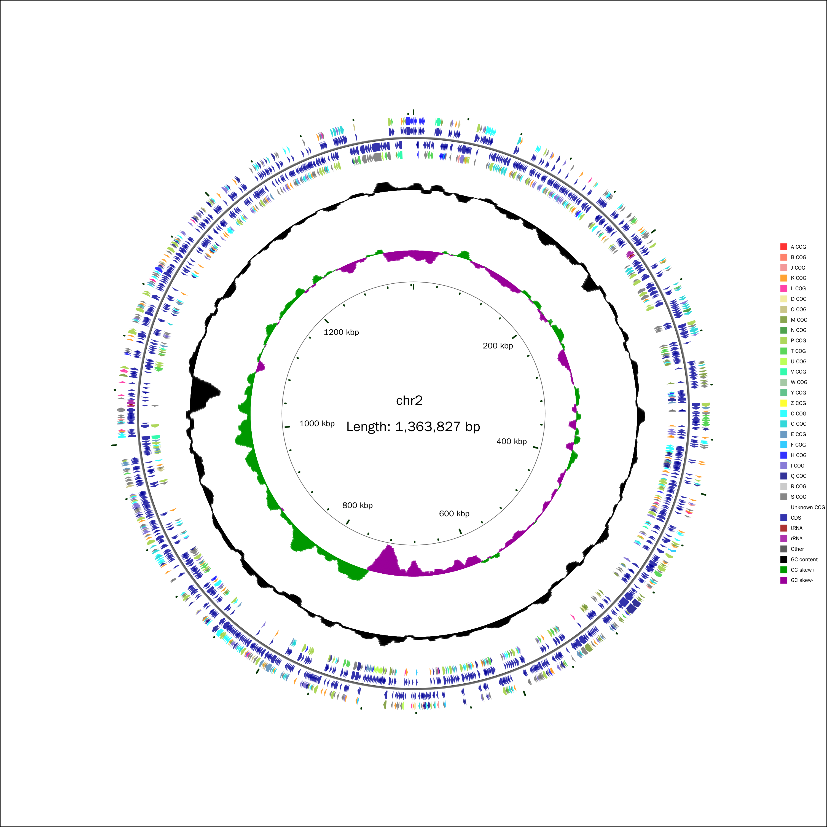

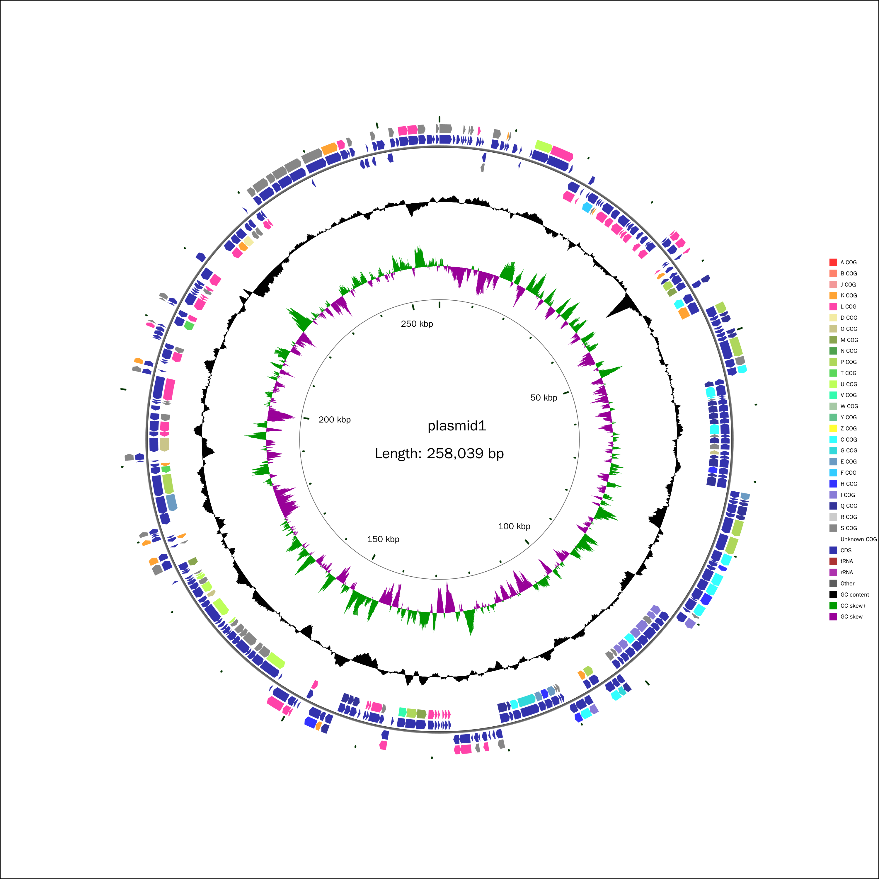

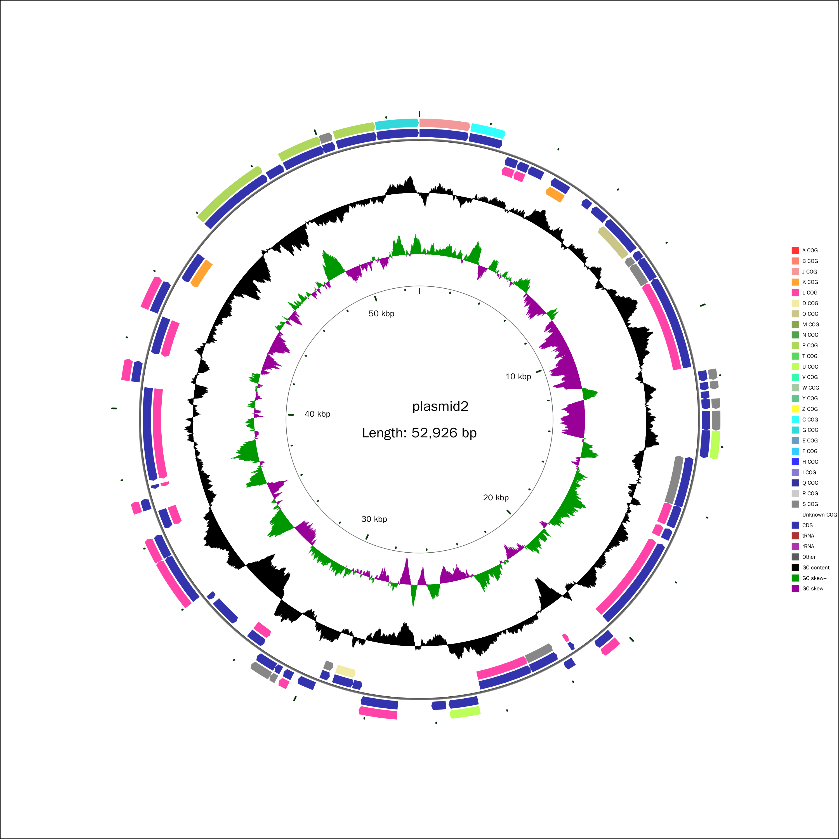


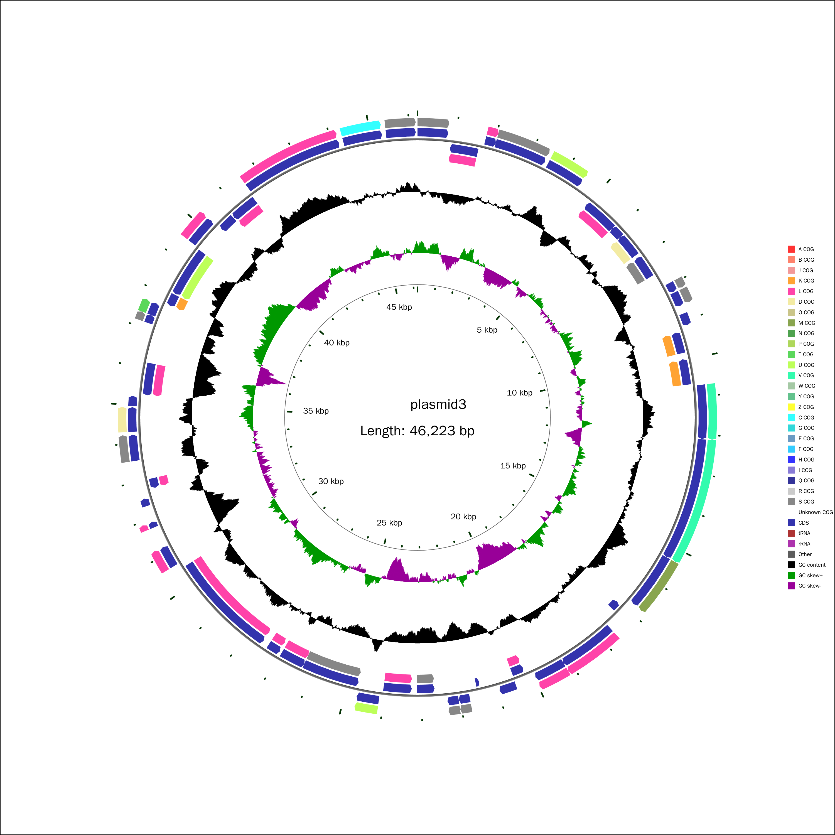

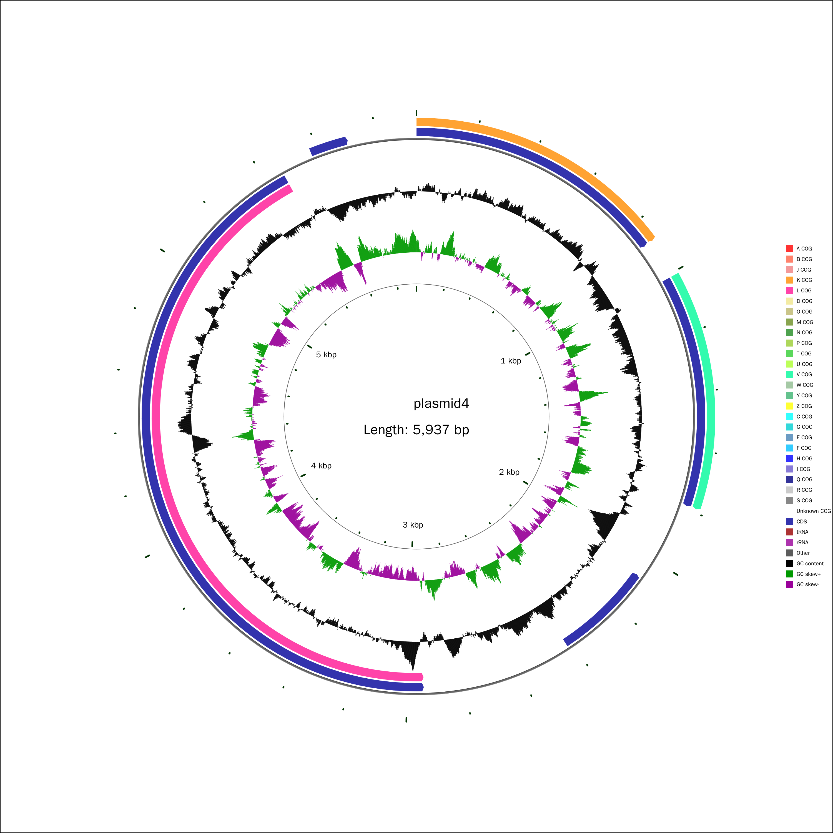


**Fig. S3. Genome architecture of *Sphingobium* sp. SHPJ-2.**
The complete genome of *Sphingobium* sp. SHPJ-2 consists of two circular chromosomes (chromosome 1, 3,663,752 bp; chromosome 2, 1,363,827 bp) and four plasmids (plasmid 1, 258,039 bp; plasmid 2, 52,926 bp; plasmid 3, 46,223 bp; and plasmid 4, 5,937 bp). The key gene clusters associated with phenanthrene degradation are predominantly located on plasmid 1.


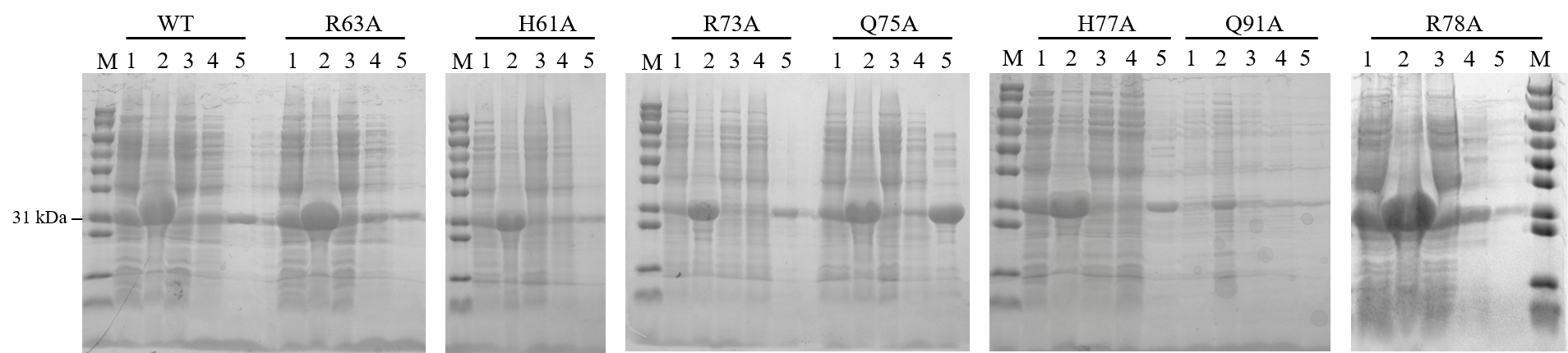


**Fig. S4. SDS–PAGE and gel filtration analyses of recombinant PheR and single-site variants.** Recombinant 6×His-tagged PheR (WT) and the indicated single-amino-acid variants (H61, R63, R73, Q75, H77, R78and Q91) were overexpressed in *Escherichia coli* BL21(DE3) from pET-28a and purified by Ni–NTA affinity chromatography. SDS–PAGE gels were stained with Coomassie Brilliant Blue R-250. Lane M, protein molecular weight marker. Each panel corresponds to one construct (WT or variant). Within each panel, lanes 1–5 represent the soluble fraction (supernatant), insoluble fraction (pellet), flow-through, 50 mM imidazole wash, and 200 mM imidazole elution, respectively. The purified proteins show a predominant band at ~30.4 kDa, consistent with the predicted molecular mass of PheR.


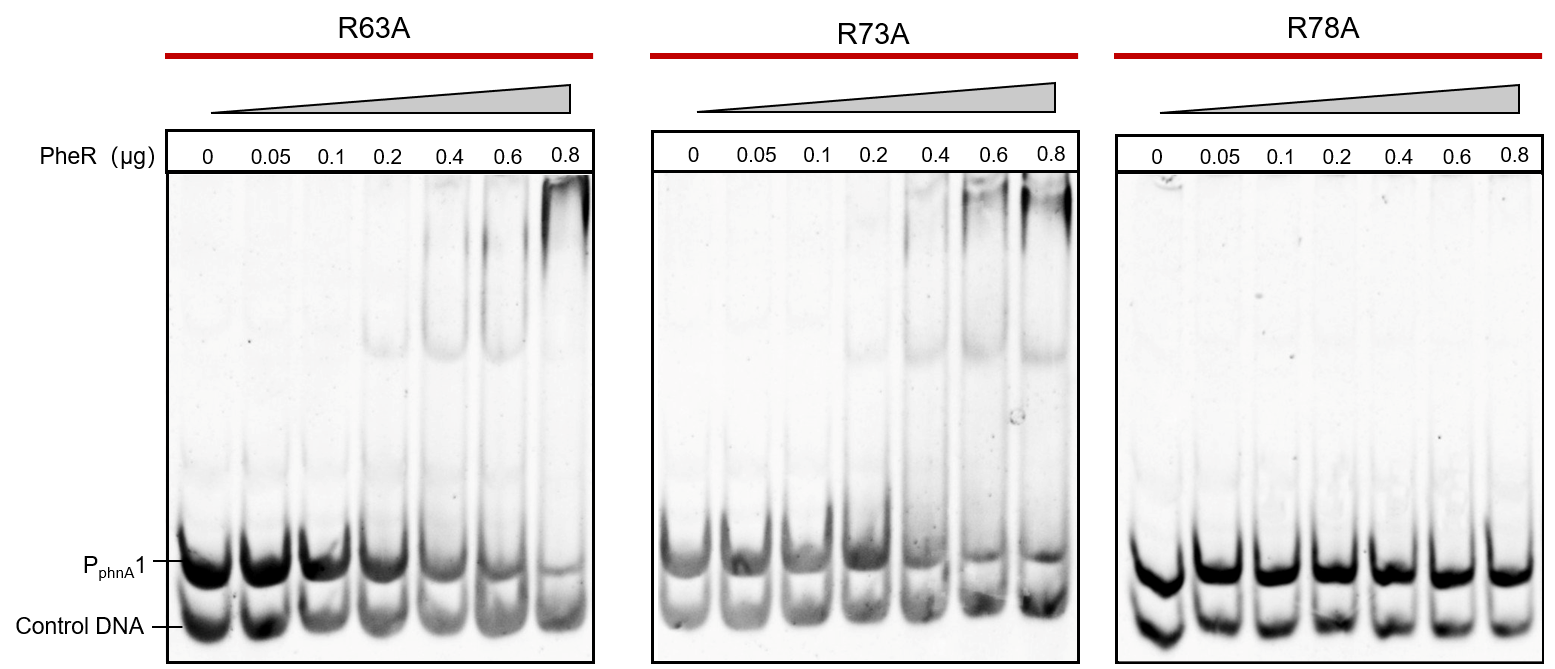


**Fig. S5. EMSA analysis of concentration-dependent binding of selected PheR variants to P_phnA1_.** Purified R63A, R73A, and R78A variants were incubated with the P_phnA1_ probe at the indicated protein concentrations.


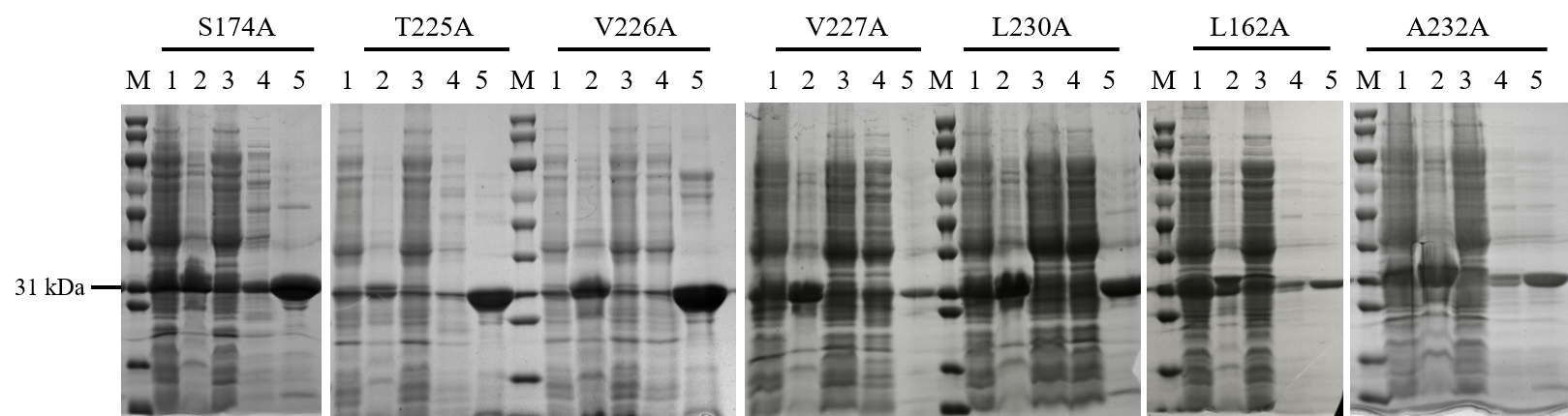


**Fig. S6. SDS–PAGE and gel filtration analyses of residue mutations of PheR and single-site variants.** Recombinant 6×His-tagged the indicated single-amino-acid variants of PheR (S174, T225A, V226A, V227A, L230A, L162A and A232A) were overexpressed in *Escherichia coli* BL21(DE3) from pET-28a and purified by Ni–NTA affinity chromatography. SDS–PAGE gels were stained with Coomassie Brilliant Blue R-250. Lane M, protein molecular weight marker. Each panel corresponds to one construct (variant). Within each panel, lanes 1–5 represent the soluble fraction (supernatant), insoluble fraction (pellet), flow-through, 50 mM imidazole wash, and 200 mM imidazole elution, respectively.


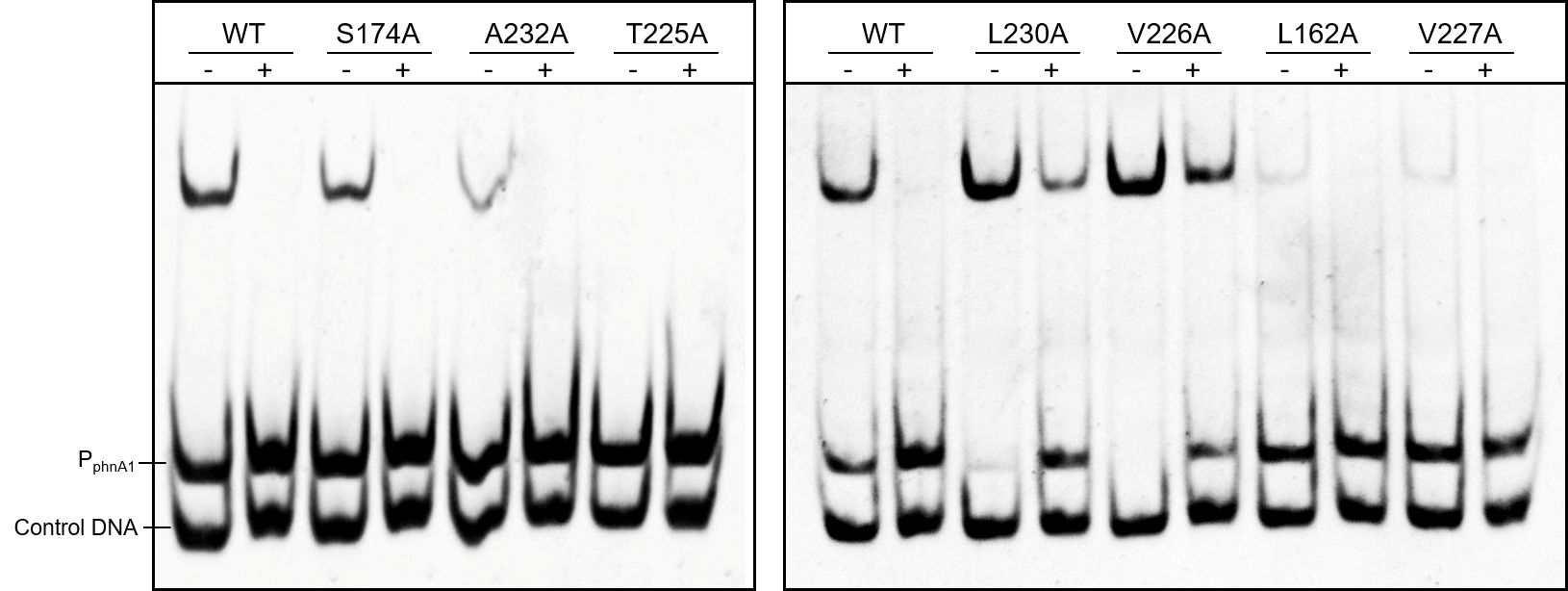


**Fig. S7.** **Effects of key residue mutations on PHE-mediated modulation of PheR-DNA binding.** Candidate residues potentially involved in PHE binding were identified by molecular docking of PheR with PHE and subjected to site-directed mutagenesis. The DNA-binding activities of wild-type and mutant PheR proteins were examined by EMSA in the absence (−) or presence (+) of 0.5 mM PHE. Each reaction contained 100 ng PheR protein and 40 ng DNA. The positions of P_phnA1_ and the control DNA are indicated.


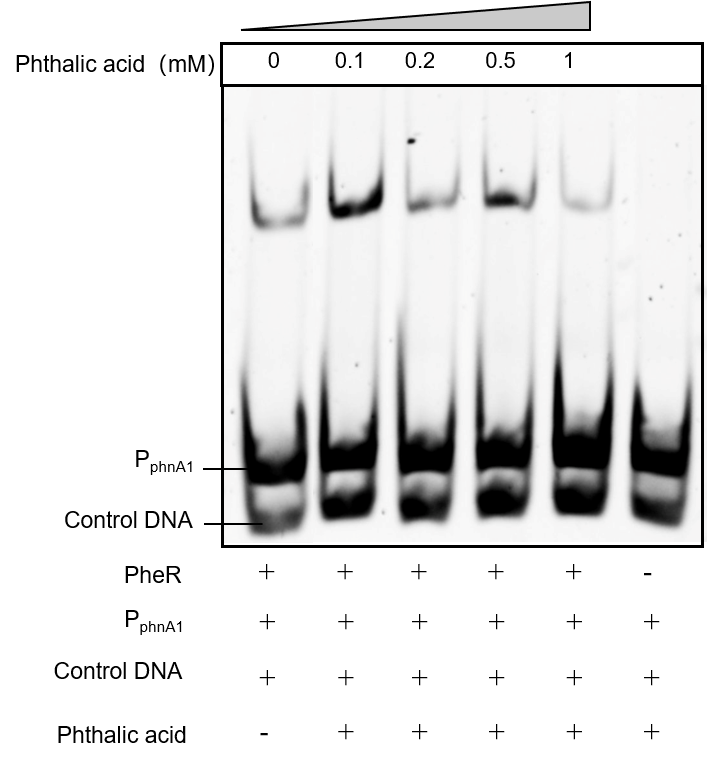


**Fig. S8. EMSA analysis of** **phthalic acid as a potential effector of PheR.**

**
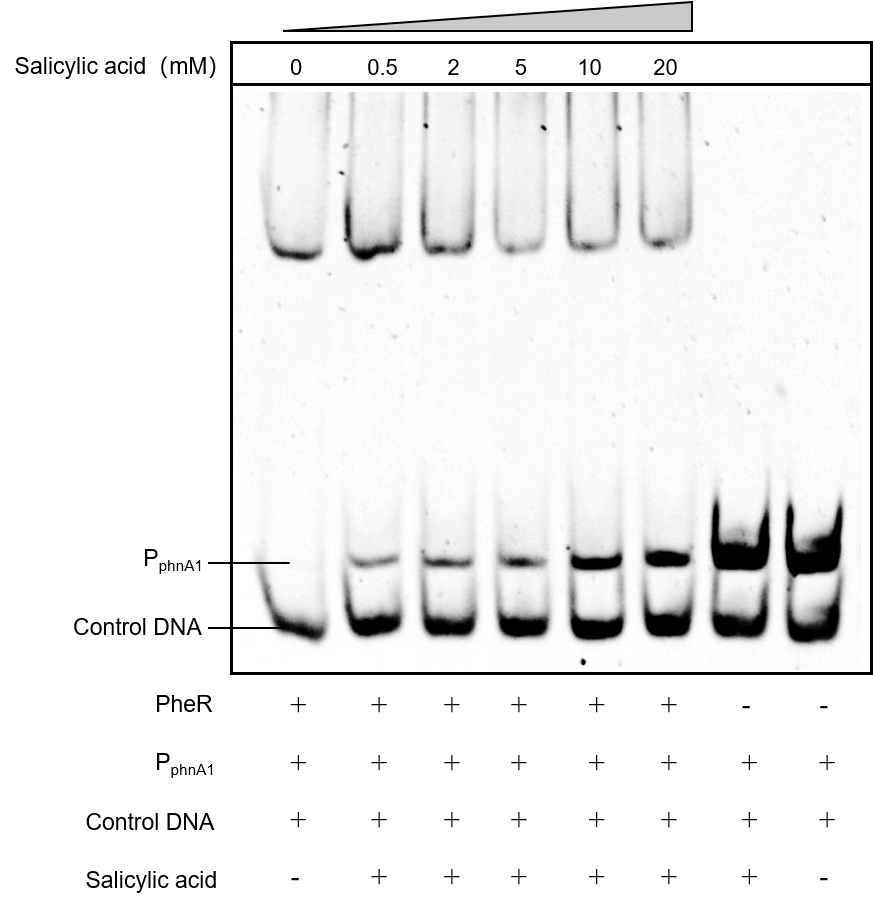
**

**Fig. S9. EMSA analysis of salicylic acid as a potential effector of PheR.**


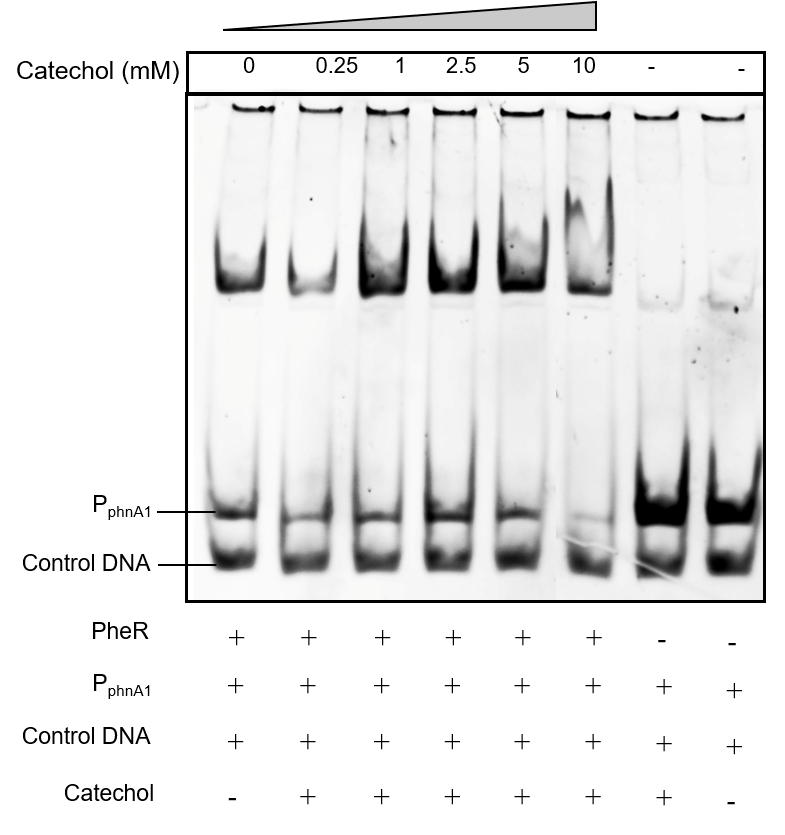


**Fig. S10. EMSA analysis of catechol as a potential effector of PheR.**


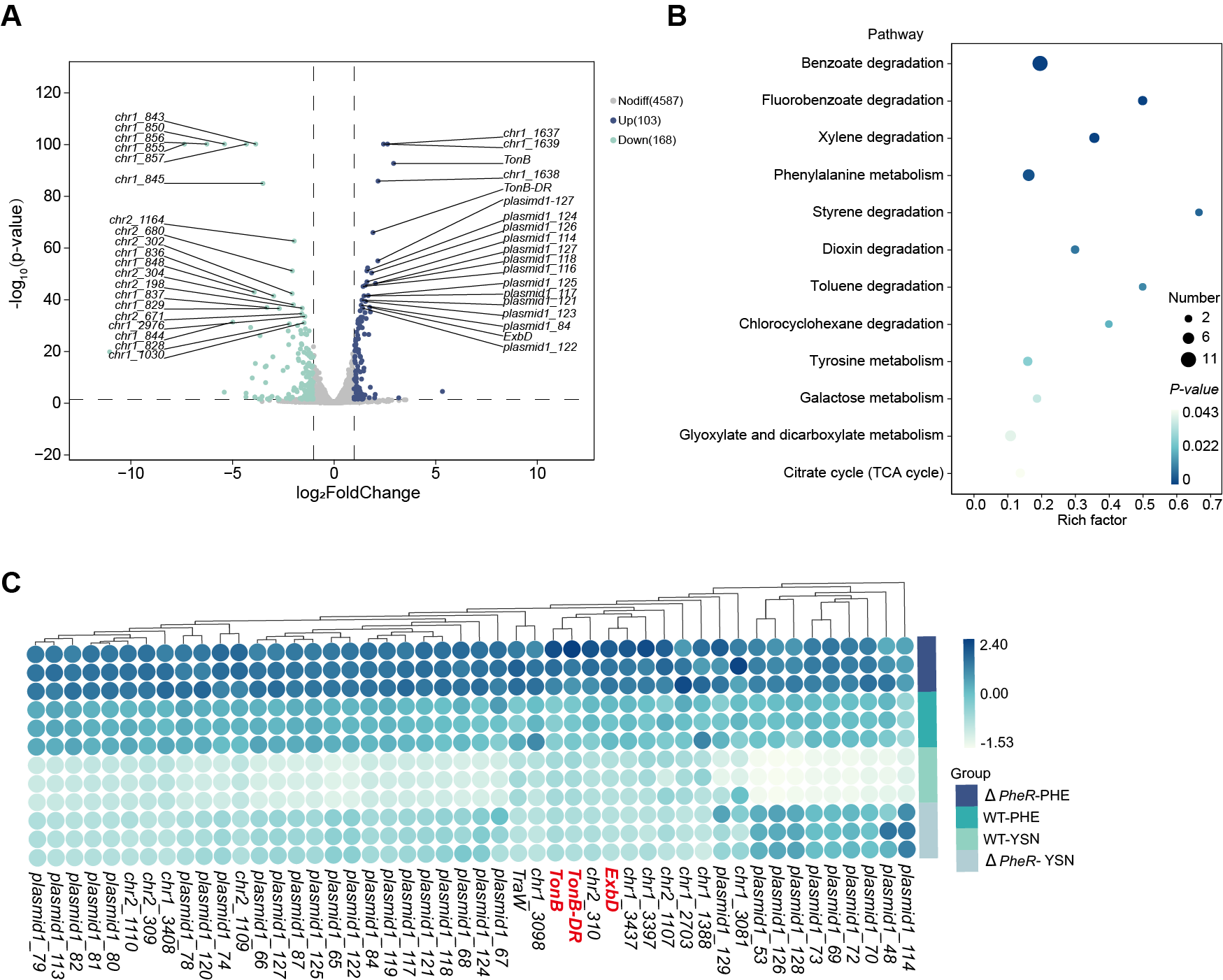


**Fig. S11. Volcano plot showing differential gene expression between wild-type SHPJ-2 and the Δ*pheR* mutant** grown in MSM with phenanthrene as the sole carbon source. The x-axis represents log₂FC and the y-axis represents –log₁₀(*p*-value). Vertical dashed lines indicate fold-change thresholds, and the horizontal dashed line marks the significance cutoff. Genes are colored according to whether they are upregulated, downregulated, or not significantly changed.


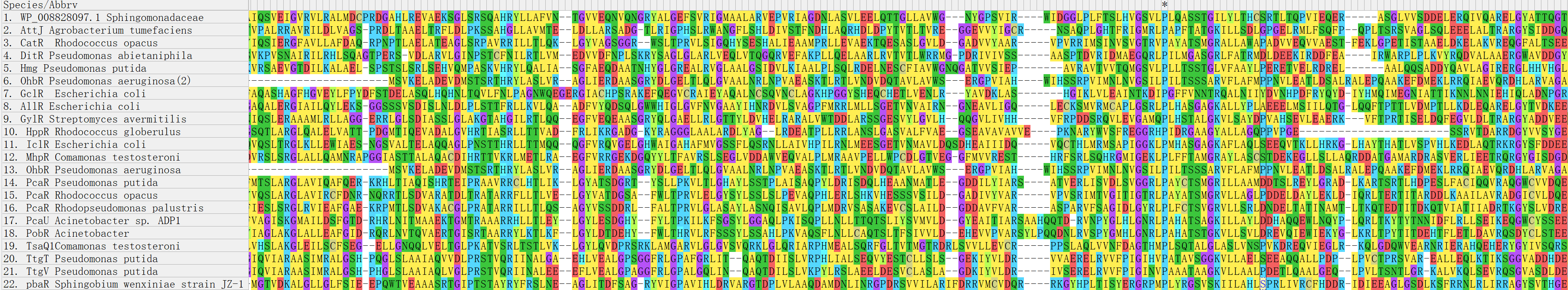


**Fig. S12. IclR family transcriptional regulators: multiple sequence alignment results.**


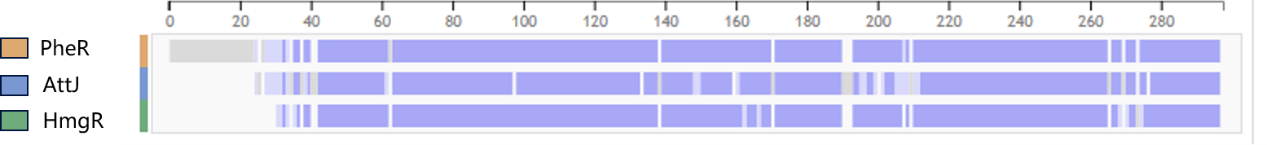

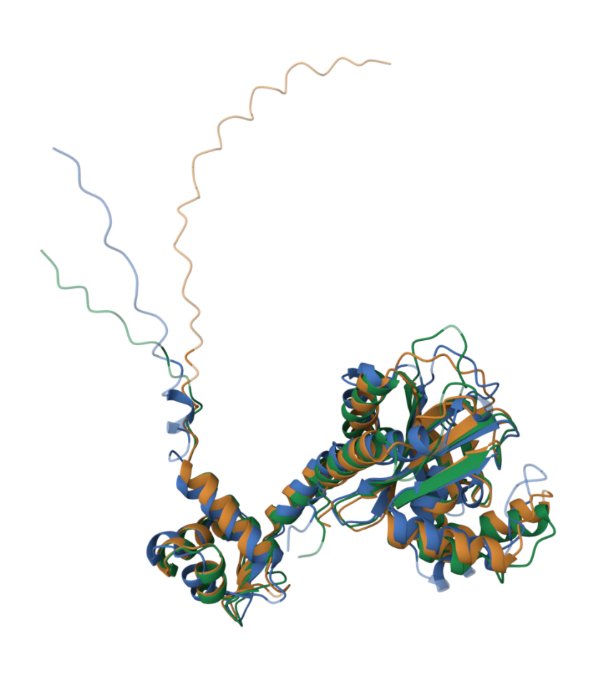


| Entry | RMSD | Tm | Identity | Align Residues | Modeled Residues | Microorganism | Accession Number |
| --- | --- | --- | --- | --- | --- | --- | --- |
| AttJ | 3.14 | 0.75 | 19% | 230 | 275 | *Agrobacterium tumefaciens* | Q8VPD8 |
| HmgR | 2.05 | 0.82 | 30% | 243 | 262 | *Pseudomonas putida* | Q6EMJ0 |

**Fig. S13. Structural alignment results of IclR family transcriptional regulators.**

**Table S1. Primers used in this study**

| Name | Primer | |  |
| --- | --- | --- | --- |
| RT-PCR | | |  |
| R9 | CGTCTTGGTTGCCAGGATGG | |  |
| R8 | CAGGGCATCGCCAATCCG | |  |
| R7 | GCTTTGAAGAGGCAGTGTACGGTG | |  |
| R6 | CTTACATGCTGTACGGCAAGAAGC | |  |
| R5 | CTGTACAGGGTAGCTGAAGTCGTG | |  |
| R4 | CTGCCTCAAGATCATCGCGG | |  |
| R3 | GCATGGAACTGCATCATGCCATC | |  |
| R2 | GCCGACACCGAAACTAAGCG | |  |
| R10 | CGAGGCGTCGTGGATCACG | |  |
| R1 | GACAGCCAGCAAACTGAGGTG | |  |
| F9 | GACGCTGGAAGGCAGCAAAG | |  |
| F8 | CTTCGCCGCTCCAAACGAC | |  |
| F7 | CGAATGATAGAGTCCATTGGACTTCG | |  |
| F6 | CTTCCAGCTCGCCGAAATAGGC | |  |
| F5 | GCACAACCGGGTTGATCACC | |  |
| F4 | CAAGGTTGAGGATCGTGTCCATCATC | |  |
| F3 | CGTTGACCTCTTGGCCGTGG | |  |
| F2 | CTTGAAGCCGCTGTCTGTTTTCC | |  |
| F10 | ATGGCCCGTAATTACCCCAGAC | |  |
| F1 | GAATCATGCCCGAGCATGAGC | |  |
| R9-2 | CATTCTGCACCGTCAGCAGC | |  |
| R8-2 | CCGGGAATCACTGGCACCATC | |  |
| R7-2 | CGGTGGAGCATCGTCGTG | |  |
| R13 | CTGATGTGATCGAGAGAGGCAAAG | |  |
| R12 | CTGCTTGATGCCTGGTTGATCG | |  |
| R11 | CCTGGGTGGCAGGCTATATGG | |  |
| F9-2 | GTCACGACGTATCGGGCTTC | |  |
| F8-2 | GCGAAGATATCCGATGTGGCG | |  |
| F7-2 | GCGAGATATGATCGGGTACCCC | |  |
| F13 | GTCTTCGTCATCCTGCCACATC | |  |
| F12 | CACTCTGACAGGCGACATGC | |  |
| F11 | CATGATCACAGCATGCGCGG | |  |
| 5’RACE | | |  |
| NGSP8 | CTTGCTTCGCCGACAGACGTTCAGGC | |  |
| NGSP7 | GCATCCCAGGTATCGTCATTGGCCCG | |  |
| NGSP6 | GTCCTTGCCCATGCTCGAATTCATCCGC | |  |
| NGSP5 | GGCACCATGGTATAGGTCCAGACCTCG | |  |
| NGSP4 | CGATCAGGCCAAACCCGTGGCCATG | |  |
| NGSP3 | CGATCATCTGGAGAGCGGCGGCATGC | |  |
| NGSP2 | GAAGCCCTTGTAAGTTTCGACCCGGATCG | |  |
| NGSP1 | GATAGCACCGCTCTTCCATCGGAACATC | |  |
| GSP8 | TTGCGGCTCGGCTTGCTTCGCCG | |  |
| GSP7 | CGGTTGGTCCACATTGCATCCCAGGTAT | |  |
| GSP6 | GGCCCTTCGCGGTCCTTGCCCAT | |  |
| GSP5 | GGTGTCGGCATTCTTCGGCACCA | |  |
| GSP4 | CAGTCGCCGCATTATCGATCAGGCCAA | |  |
| GSP3 | GCCAGCTCGCCCCCGATCATCTG | |  |
| GSP2 | CATGGCAGCCGAAGATGAAGCCCTTGTA | |  |
| GSP1 | GTCGAGATCGCTGTGATAGCACCGCTCT | |  |
| RT-QPCR | | |  |
| rpob-QPCR-F1 | | CTTGTCCAGCTTCTCCAGATT | |
| rpob-QPCR-R1 | TCCCGACCGAAGAAGTCTAT | |  |
| 16S-QPCR-F1 | CTCTCGAGTTGCAGAGAACAA | |  |
| 16S-QPCR-R1 | GTGCTACAATGGCGACTACA | |  |
| 3136-QPCR-F2 | CTCGGCATAGTCATCGTTGTAG | |  |
| 3136-QPCR-F1 | GCAAACCGCTGTTCGTAAAC | |  |
| 3136-QPCR-R2 | TCGCCAATGGTTCGATACTG | |  |
| 3136-QPCR-R1 | ATGCCATCGTCAGTCACTATTC | |  |
| 3137-QPCR-F2 | CGTATTCCAGTGACCGACATAG | |  |
| 3137-QPCR-R2 | CGACGGGTACGTCAAATATCTC | |  |
| 3137-QPCR-F1 | GAGCCCTAGCTTGGATTTGT | |  |
| 3137-QPCR-R1 | GGCGTTCGTGTGCAATTATC | |  |
| 116-QPCR-F1 | GCAAGACGAAGACCCTTACA | |  |
| 116-QPCR-R1 | TGGTGGATCAGGTGGTAATTC | |  |
| 117-QPCR-F1 | TTTACCACCATGTTCCCTTACC | |  |
| 117-QPCR-R1 | AGCGTTTCGCCTTCGTATT | |  |
| 118-QPCR-F1 | CTTGCGGGCGACCTTATT | |  |
| 118-QPCR-R1 | TCAGGAACTTGTCCCACAAC | |  |
| 121-QPCR-F1 | GGTTGAGGTTAGCAGAAGGAG | |  |
| 121-QPCR-R1 | GTTCAATGTGGTGGTTGGTTC | |  |
| 122-QPCR-F1 | CCTTGGTCAGCAGGATGTAATA | |  |
| 122-QPCR-R1 | CGGGATGGCCTGAGTATTT | |  |
| 123-QPCR-F1 | CACAGGAAGTTGTCGTGATAGA | |  |
| 123-QPCR-R1 | GCCATTTCGCCAAGGAATAC | |  |
| 124-QPCR-F1 | CTTTGCGCGATGCCATTT | |  |
| 124-QPCR-R1 | CGTTTCCTGGGTGCTTATGT | |  |
| 125-QPCR-F2 | CAACCTTGACGCAGTTGTTT | |  |
| 125-QPCR-R2 | ATGGTTCGACGCCGATTAC | |  |
| 125-QPCR-F1 | CGGCGTCGGGTAATATTGAT | |  |
| 125-QPCR-R1 | CAGGGTGGTGAATTCGTTCT | |  |
| Plasmid construction | | |  |
| pET-28a-F | atggctagcatgactggtgga | |  |
| pET-28a-R | GTGATGATGATGATGATGGCTGC | |  |
| iclR-28a-F | catcatcatcatcatcacATGAACGCACGCAAGGCTAGC | |  |
| iclR-28a-R | ccaccagtcatgctagccatCTATCCTTGCCAGCCGATCG | |  |
| 28a-iclR-YZ-F | gtagaggatcgagatctcgatcccg | |  |
| 28a-iclR-YZ-R | ctcagcttcctttcgggctttg | |  |
| PET18Tc-YZ-F1 | GCAACTGTTGGGAAGGGCG | |  |
| PET18Tc-R | TACCCGGGGATCCTCTAGAGTC | |  |
| iclR-A-F | CTCTAGAGGATCCCCGGGTAGATCTGGCCGATCGACACAAG | |  |
| PET18Tc-YZ-R1 | GTCCTTCCTCTGGCGATCGTTC | |  |
| iclR-A-R | GGGAATACCGCTCCTGCGGCTTTACATCAGAAGTG | |  |
| iclR-B-F | GCCGCAGGAGCGGTATTCCCCTTCACCAGCC | |  |
| PET18Tc-YZ-F2 | CAACGACCTGGTACCTGGCG | |  |
| iclR-B-R | GATTACGAATTCGAGCTCGGCGTACTTGAGGTAATAGGCCTTGTGC | |  |
| PET18Tc-F | CCGAGCTCGAATTCGTAATCA | |  |
| PET18Tc-YZ-R2 | GAGCGCAACGCAATTAATGTGAG | |  |
| ORI-R | GGGAGTTCATCGCCATGATGCAGTGTGACTCTAGTAGAGAGCGTTC | |  |
| ICLR-F | CATCATGGCGATGAACTCCCGT | |  |
| ICLR-R | CCATGGAAACGACAAACAAGAGAGC | |  |
| ORI-F | CTTGTTTGTCGTTTCCATGGCTTGCCAGCCCGTGGATATG | |  |
| ORI-R2 | GGGAGTTCATCGCCATGATGCAGTGTGACTCTAGTAGAGAGCGTTC | |  |
| ICLR-F2 | CATCATGGCGATGAACTCCCGT | |  |
| ICLR-R2 | CCATGGAAACGACAAACAAGAGAGC | |  |
| ORI-F2 | CTTGTTTGTCGTTTCCATGGCTTGCCAGCCCGTGGATATG | |  |
| PP127-F | GTCGACACCATCGAATGGTGCACCGCCAGAACGCCAG | |  |
| BONE-R | CACCATTCGATGGTGTCGAC | |  |
| PP127-R | ACGTCTTCGCTACTCGCCATCGCATCGCTCCCATGCTTTTTG | |  |
| BONE-F | ATGGCGAGTAGCGAAGACG | |  |
| EMSA |  | |  |
| 127-F1 | CATCGCATCGCTCCCATG | |  |
| 127-R1 | CATCTTGCAACCTAACATTCTCCCC | |  |
| control-R | GGATGCCGCAATCTACAACG | |  |
| control-F | GATTCGTGATGGCGGATCTTCC | |  |
| Genetic disruption and complementation | | |  |
| PEX18-3137L-A-F | CTCTAGAGGATCCCCGGGTATCACTGCGCTGAGGCCCA | |  |
| PEX18-3137L-A-R | ATGCGTCCCGACAGCAATCTGGACC | |  |
| PEX18-3137L-C-F | AGATTGCTGTCGGGACGCATCGCTCCCATGCTT | |  |
| PEX18-3137L-C-R | GATTACGAATTCGAGCTCGGTTATGTCTTGACCCGACGCG | |  |
| PET18Tc-YZ-F1 | GCAACTGTTGGGAAGGGCG | |  |
| PET18Tc-R | TACCCGGGGATCCTCTAGAGTC | |  |
| iclR-A-F | CTCTAGAGGATCCCCGGGTAGATCTGGCCGATCGACACAAG | |  |
| PET18Tc-YZ-R1 | GTCCTTCCTCTGGCGATCGTTC | |  |
| iclR-A-R | GGGAATACCGCTCCTGCGGCTTTACATCAGAAGTG | |  |
| iclR-B-F | GCCGCAGGAGCGGTATTCCCCTTCACCAGCC | |  |
| PET18Tc-YZ-F2 | CAACGACCTGGTACCTGGCG | |  |
| iclR-B-R | GATTACGAATTCGAGCTCGGCGTACTTGAGGTAATAGGCCTTGTGC | |  |
| PET18Tc-F | CCGAGCTCGAATTCGTAATCA | |  |
| PET18Tc-YZ-R2 | GAGCGCAACGCAATTAATGTGAG | |  |
| pheR-F | ACATTAATTGCGTTGCGCCACCGCCAGAACGCCAG | |  |
| pheR-R | GTTCTTCTCCTTTACGCATCGCATCGCTCCCATGCTTTTTG | |  |

**Table. S2. Strains used in this study.**

| Name | Strain | Source |
| --- | --- | --- |
| BL21(DE3)-pET28a-PheR | *E. coli* | This study |
| DH5α-pBBRMCS-P_phnA1_-RFP | *E. coli* | This study |
| DH5α- pBBRMCS-*pheR*-P_phnA1_-RFP | *E. coli* | This study |
| SHPJ-2 | *Sphingobium* sp. | This study |
| SHPJ-2Δ*pheR* | *Sphingobium* sp. | This study |
| SHPJ-2Δ*pheR*::*pheR* | *Sphingobium* sp. | This study |
| SHPJ-2Δ*pheR*-pBBRMCS (EV) | *Sphingobium* sp. | This study |
| SHPJ-2Δ*pheR*-pBBRMCS-P_phnA1_-RFP | *Sphingobium* sp. | This study |
| SHPJ-2Δ*pheR*-pBBRMCS-*pheR*-P_phnA1_-RFP | *Sphingobium* sp. | This study |
| BGR4 | *Pseudomonas putida* | This study |
| BGR4-pBBRMCS-P_phnA1_-RFP | *Pseudomonas* *putida* | This study |
| BGR4-pBBRMCS-*pheR*-P_phnA1_-RFP | *Pseudomonas* *putida* | This study |

**Table. S3. The gene sequence of *pheR***

| Gene | Sequence |
| --- | --- |
| *pheR* | ***ATG***AACGCACGCAAGGCTAGCCAGCTTACAGCTAAAGGTGATGAAATCGAACATCAGGCTAACGAGCGCTTGCCTCCCGAAGATTTGAGCGCGAATGATTCGCCGCGCAAGGCTATCCAGTCCGTGGAAATTGGTGTTCGCGTCCTCAGGGCCTTGATGGATTGCCCAAGGGATGGCGCACATCTGCGTGAAGTTGCCGAGAAATCGGGTCTGTCACGCAGCCAGGCACACCGTTACCTGCTGGCTTTTGTCAATACAGGCGTGGTCGAGCAGAATGTCCAGAATGGCCGCTATGCACTAGGTGAGTTTTCAGTTCGTATTGGCATGGCCGCGCTGGCGCGGGTGGAGCCGGTACGGATCGCTGGCGATAATCTGGCGAGCGTGCTGGAAGAACTGCAAACGACAGGGTTGCTGGCGGTCTGGGGTAATTACGGGCCATCGGTGATTCGCTGGATCGATGGCGGTCTGCCCCTGTTTACCAGTTTGCACGTCGGCTCTGTTTTGCCATTGCAGGCTTCGTCGACTGGCATCCTGTATTTGACCCATTGTTCACGAACTTTGACCCAGCCCGTCATCGAGCAAGAGCGCGCCAGCGGGCTGGTGGTTTCCGACGACGAACTGGAAAGGCAGATAGTCCAAGCGCGCGAGCTGGGATACGCGACCACACAGGGTACGGTTGTGCCTGGTCTCTCTGCCTTGAGTGTACCTGTGTTTGATTCCGCGAACAGGCTTGTTGCGACGATGAGCGTTCTCTCGCGAGTTCAGGACAAGCATTTTTATTCGAAGACGAAGATCGAAAAGATCCTTTCGGCGGCCATAGCAGCGAGTCGTGCGATCGGCTGGCAAGGATAG |

**Table. S4. Raw data used for construction of the structure-based phylogenetic tree in Figure S2**

| ID | baitID | TM_score | log_2_FoldChange | *p-*adj |
| --- | --- | --- | --- | --- |
| plasmid1_82 | ORF3137 | 8.41E-01 | 5.208432891 | 0.002514751 |
| plasmid1_79 | ORF3137 | 7.69E-01 | 5.309794043 | 3.88427E-12 |
| plasmid1_86 | ORF3137 | 7.66E-01 | 5.33291035 | 3.45904E-12 |
| plasmid1_50 | ORF3137 | 7.61E-01 | 3.956799401 | 3.56848E-07 |
| plasmid2_53 | ORF3137 | 7.30E-01 | -1.221422867 | 0.268582449 |
| chr2_88 | ORF3137 | 7.22E-01 | -1.149448145 | 0.26879229 |
| chr1_1110 | ORF3137 | 3.42E-01 | 2.897721883 | 0.024539733 |
| chr2_369 | ORF3137 | 3.30E-01 | 2.352471849 | 0.195942302 |
| chr1_1381 | ORF3137 | 3.24E-01 | 2.814709972 | 0.042122491 |
| chr2_917 | ORF3137 | 3.22E-01 | 2.525394411 | 0.297587046 |
| chr1_3467 | ORF3137 | 3.19E-01 | 1.306601587 | 0.231950971 |
| chr1_3272 | ORF3137 | 3.14E-01 | 2.490707158 | 0.230100063 |
| chr1_2520 | ORF3137 | 3.12E-01 | 1.657070699 | 0.074767813 |
| chr1_1157 | ORF3137 | 3.10E-01 | 1.645620516 | 0.087935145 |
| chr1_1159 | ORF3137 | 3.09E-01 | 1.212241152 | 0.28177137 |
| plasmid1_94 | ORF3137 | 3.09E-01 | 2.942503189 | 0.142149642 |
| chr1_469 | ORF3137 | 3.09E-01 | 1.343004202 | 0.207612726 |
| chr1_1323 | ORF3137 | 3.08E-01 | 1.789361586 | 0.047692449 |
| chr2_906 | ORF3137 | 3.06E-01 | -1.77019239 | 0.051495772 |
| chr1_1900 | ORF3137 | 3.06E-01 | 1.254180157 | 0.24728558 |
| chr1_1650 | ORF3137 | 3.04E-01 | -1.204539109 | 0.268582449 |
| chr1_1637 | ORF3137 | 3.02E-01 | 4.564369444 | 0.02926395 |
| chr2_1080 | ORF3137 | 3.01E-01 | -1.825267213 | 0.047692449 |
| chr1_2372 | ORF3137 | 3.01E-01 | 1.451312136 | 0.150366342 |
| plasmid1_208 | ORF3137 | 3.01E-01 | -1.499853524 | 0.131404666 |
| chr1_185 | ORF3137 | 3.00E-01 | 2.116441461 | 0.044131293 |
| chr2_1144 | ORF3137 | 2.99E-01 | -1.181620312 | 0.28011526 |
| chr1_2014 | ORF3137 | 2.98E-01 | 1.381781827 | 0.212854158 |
| plasmid1_88 | ORF3137 | 2.98E-01 | 4.175761894 | 0.001763405 |
| plasmid1_98 | ORF3137 | 2.98E-01 | 2.912702642 | 0.150361795 |
| chr1_2424 | ORF3137 | 2.97E-01 | 1.276536591 | 0.24080612 |
| chr1_2521 | ORF3137 | 2.97E-01 | 1.192339013 | 0.271205241 |
| chr1_3402 | ORF3137 | 2.96E-01 | 1.995550217 | 0.042122491 |
| chr2_219 | ORF3137 | 2.96E-01 | -1.168061641 | 0.281354716 |
| chr1_2621 | ORF3137 | 2.95E-01 | 1.468977981 | 0.152583041 |
| chr1_694 | ORF3137 | 2.95E-01 | 3.552387856 | 0.068058232 |
| chr1_1181 | ORF3137 | 2.94E-01 | 1.467680644 | 0.148715217 |
| plasmid1_107 | ORF3137 | 2.93E-01 | 2.002932211 | 0.021216181 |
| chr2_975 | ORF3137 | 2.93E-01 | -1.248862084 | 0.265105431 |
| chr1_18 | ORF3137 | 2.92E-01 | 3.556405873 | 0.104723738 |
| chr1_1772 | ORF3137 | 2.92E-01 | 1.358353502 | 0.194668034 |
| chr1_2091 | ORF3137 | 2.91E-01 | 1.217520304 | 0.269395047 |
| chr1_3012 | ORF3137 | 2.91E-01 | 2.215177554 | 0.008275177 |
| chr1_1506 | ORF3137 | 2.90E-01 | 2.38799262 | 0.004221775 |
| chr1_1442 | ORF3137 | 2.90E-01 | 1.297851748 | 0.225534354 |
| chr1_1639 | ORF3137 | 2.90E-01 | 3.910148368 | 0.000362028 |
| chr1_1068 | ORF3137 | 2.89E-01 | 1.849970605 | 0.196764159 |
| plasmid1_117 | ORF3137 | 2.89E-01 | 3.405328153 | 7.23296E-06 |
| chr1_2395 | ORF3137 | 2.89E-01 | 2.428341001 | 0.174196282 |
| chr1_771 | ORF3137 | 2.89E-01 | 2.425237873 | 0.065396712 |
| chr1_1254 | ORF3137 | 2.88E-01 | -1.879845163 | 0.039127259 |
| plasmid1_89 | ORF3137 | 2.88E-01 | 4.913812415 | 0.017835536 |
| chr1_220 | ORF3137 | 2.88E-01 | 2.511534181 | 0.114675898 |
| chr2_471 | ORF3137 | 2.88E-01 | 3.01154354 | 0.219428145 |
| chr2_616 | ORF3137 | 2.87E-01 | -3.695430865 | 9.04956E-07 |
| chr1_2066 | ORF3137 | 2.87E-01 | 1.567324379 | 0.108789204 |
| chr1_437 | ORF3137 | 2.87E-01 | 2.767188112 | 0.054944841 |
| chr1_289 | ORF3137 | 2.87E-01 | 1.905468715 | 0.291496774 |
| chr1_3522 | ORF3137 | 2.86E-01 | -1.878681297 | 0.03496794 |
| chr1_2957 | ORF3137 | 2.85E-01 | 1.864641827 | 0.040249198 |
| chr1_473 | ORF3137 | 2.85E-01 | 1.727639303 | 0.063928547 |
| chr1_1437 | ORF3137 | 2.84E-01 | 1.79204264 | 0.051988073 |
| chr1_1367 | ORF3137 | 2.83E-01 | 1.371201409 | 0.183238068 |
| chr2_118 | ORF3137 | 2.83E-01 | 1.992594799 | 0.219428145 |
| chr2_928 | ORF3137 | 2.82E-01 | 2.121526255 | 0.020608478 |
| chr1_1792 | ORF3137 | 2.81E-01 | 1.69032435 | 0.188200754 |
| chr1_2512 | ORF3137 | 2.81E-01 | 2.677080899 | 0.000933615 |
| chr1_1435 | ORF3137 | 2.81E-01 | 1.840881775 | 0.044131293 |
| chr1_1872 | ORF3137 | 2.81E-01 | 1.174634848 | 0.291496774 |
| chr1_1992 | ORF3137 | 2.81E-01 | 2.054887659 | 0.274260144 |
| chr1_526 | ORF3137 | 2.80E-01 | 1.203231121 | 0.300405059 |
| chr1_796 | ORF3137 | 2.80E-01 | 2.045809325 | 0.228394361 |
| chr1_2469 | ORF3137 | 2.78E-01 | 2.122740932 | 0.131404666 |
| chr1_1428 | ORF3137 | 2.78E-01 | 1.411966899 | 0.267181035 |
| chr2_805 | ORF3137 | 2.78E-01 | -1.281594661 | 0.235889706 |
| chr2_1071 | ORF3137 | 2.78E-01 | -1.236856054 | 0.289081173 |
| chr1_2506 | ORF3137 | 2.78E-01 | 1.173208004 | 0.295975268 |
| chr1_808 | ORF3137 | 2.78E-01 | 1.755420655 | 0.060439545 |
| chr2_1067 | ORF3137 | 2.77E-01 | -1.315650234 | 0.237775422 |
| chr1_1185 | ORF3137 | 2.76E-01 | 2.645416214 | 0.00110292 |
| chr1_2691 | ORF3137 | 2.76E-01 | 1.90615315 | 0.030388919 |
| plasmid1_100 | ORF3137 | 2.76E-01 | 1.200568018 | 0.288847328 |
| chr1_1410 | ORF3137 | 2.75E-01 | 1.398273447 | 0.214821393 |
| chr1_2272 | ORF3137 | 2.74E-01 | 1.153025741 | 0.2988682 |
| chr1_2580 | ORF3137 | 2.74E-01 | 1.993487402 | 0.023250265 |
| chr1_217 | ORF3137 | 2.73E-01 | 2.884512612 | 0.000294043 |
| chr1_732 | ORF3137 | 2.73E-01 | -1.325160906 | 0.201171402 |
| chr1_1443 | ORF3137 | 2.73E-01 | 1.932085648 | 0.030388919 |
| chr2_1103 | ORF3137 | 2.72E-01 | -2.259775672 | 0.007394775 |
| chr1_781 | ORF3137 | 2.72E-01 | 3.858970829 | 6.58782E-07 |
| chr1_3542 | ORF3137 | 2.71E-01 | 1.182991988 | 0.28105471 |
| chr1_2306 | ORF3137 | 2.71E-01 | -1.187867952 | 0.286679306 |
| chr1_1520 | ORF3137 | 2.70E-01 | -1.193066341 | 0.267181035 |
| chr1_2689 | ORF3137 | 2.70E-01 | 2.29484182 | 0.203665761 |
| chr1_1151 | ORF3137 | 2.70E-01 | 1.83144332 | 0.049236158 |
| plasmid1_125 | ORF3137 | 2.70E-01 | 5.39162421 | 1.10707E-11 |
| chr1_2640 | ORF3137 | 2.70E-01 | 1.526901291 | 0.119620336 |
| plasmid1_114 | ORF3137 | 2.69E-01 | 1.887219368 | 0.037548453 |
| chr2_932 | ORF3137 | 2.69E-01 | 2.645415245 | 0.001076663 |
| chr1_45 | ORF3137 | 2.69E-01 | 1.200217813 | 0.279696224 |
| chr2_635 | ORF3137 | 2.69E-01 | -1.326446355 | 0.188708343 |
| chr2_550 | ORF3137 | 2.69E-01 | 3.013089487 | 0.213622874 |
| chr1_1683 | ORF3137 | 2.68E-01 | -1.454090942 | 0.180273433 |
| chr2_920 | ORF3137 | 2.68E-01 | -1.327943376 | 0.196764159 |
| chr1_214 | ORF3137 | 2.68E-01 | 2.549882749 | 0.219428145 |
| chr2_1104 | ORF3137 | 2.67E-01 | -1.755553771 | 0.062572263 |
| plasmid1_51 | ORF3137 | 2.66E-01 | 3.904419949 | 2.5919E-07 |
| chr1_3279 | ORF3137 | 2.66E-01 | 1.81637033 | 0.183238068 |
| chr2_531 | ORF3137 | 2.65E-01 | 1.221725327 | 0.271205241 |
| chr1_2489 | ORF3137 | 2.65E-01 | 1.687916733 | 0.069892399 |
| chr1_3301 | ORF3137 | 2.65E-01 | 1.317192234 | 0.212703054 |
| chr1_1306 | ORF3137 | 2.65E-01 | 1.819501074 | 0.047346557 |
| chr1_2046 | ORF3137 | 2.65E-01 | 1.83333048 | 0.121978679 |
| chr1_281 | ORF3137 | 2.65E-01 | 1.940576774 | 0.269198227 |
| chr2_1093 | ORF3137 | 2.64E-01 | 2.192006168 | 0.176071385 |
| chr1_3318 | ORF3137 | 2.64E-01 | -1.685569845 | 0.068058232 |
| chr1_440 | ORF3137 | 2.64E-01 | 1.249631995 | 0.239531092 |
| chr1_698 | ORF3137 | 2.63E-01 | 1.797726204 | 0.052952553 |
| chr1_1868 | ORF3137 | 2.63E-01 | 2.554035724 | 0.144078549 |
| chr2_440 | ORF3137 | 2.63E-01 | -1.366871168 | 0.235368484 |
| chr1_3026 | ORF3137 | 2.62E-01 | 2.717883224 | 0.088378086 |
| chr1_74 | ORF3137 | 2.62E-01 | 7.137376958 | 0.013884955 |
| chr2_922 | ORF3137 | 2.61E-01 | -1.400309166 | 0.169232214 |
| chr1_400 | ORF3137 | 2.61E-01 | -1.184295522 | 0.288847328 |
| chr1_2013 | ORF3137 | 2.61E-01 | 1.731276736 | 0.196764159 |
| plasmid1_122 | ORF3137 | 2.60E-01 | 4.785598078 | 2.3458E-10 |
| chr2_1058 | ORF3137 | 2.60E-01 | -1.13400854 | 0.295975268 |
| plasmid1_53 | ORF3137 | 2.60E-01 | 3.724172359 | 9.04956E-07 |
| chr2_473 | ORF3137 | 2.60E-01 | 1.848847711 | 0.196764159 |
| chr1_1239 | ORF3137 | 2.59E-01 | 1.385418383 | 0.183238068 |
| chr1_590 | ORF3137 | 2.59E-01 | 1.331484569 | 0.281280664 |
| chr1_204 | ORF3137 | 2.59E-01 | 2.281561695 | 0.191528226 |
| plasmid1_108 | ORF3137 | 2.59E-01 | 1.934307317 | 0.269924678 |
| chr1_2147 | ORF3137 | 2.59E-01 | 1.507917349 | 0.129967853 |
| chr2_1045 | ORF3137 | 2.58E-01 | -1.175376544 | 0.27636851 |
| chr1_319 | ORF3137 | 2.58E-01 | 2.10508771 | 0.016558641 |
| chr1_802 | ORF3137 | 2.58E-01 | 1.613280209 | 0.09071389 |
| chr1_1141 | ORF3137 | 2.58E-01 | 1.393543074 | 0.179557954 |
| chr1_2129 | ORF3137 | 2.57E-01 | 1.580303805 | 0.111067991 |
| chr1_1891 | ORF3137 | 2.57E-01 | 1.706180983 | 0.070900961 |
| chr1_394 | ORF3137 | 2.57E-01 | 1.205929233 | 0.267181035 |
| chr1_1039 | ORF3137 | 2.57E-01 | -1.506199525 | 0.125963579 |
| chr1_1231 | ORF3137 | 2.57E-01 | 2.179721325 | 0.196764159 |
| chr1_1986 | ORF3137 | 2.56E-01 | 2.155000249 | 0.011706044 |
| chr1_1436 | ORF3137 | 2.56E-01 | 1.738443634 | 0.062305685 |
| chr1_1322 | ORF3137 | 2.55E-01 | 1.843329384 | 0.08683018 |
| chr1_1242 | ORF3137 | 2.55E-01 | 1.346911075 | 0.212854158 |
| chr1_1476 | ORF3137 | 2.55E-01 | 1.331744451 | 0.212994979 |
| chr1_270 | ORF3137 | 2.55E-01 | 2.172501575 | 0.115769811 |
| chr1_1441 | ORF3137 | 2.55E-01 | 2.048419792 | 0.020022857 |
| chr1_3223 | ORF3137 | 2.54E-01 | 1.777121084 | 0.05368412 |
| chr1_2511 | ORF3137 | 2.54E-01 | 1.955098302 | 0.026714553 |
| chr1_258 | ORF3137 | 2.54E-01 | 2.611753936 | 0.00123291 |
| chr2_638 | ORF3137 | 2.54E-01 | 2.739549999 | 0.00950286 |
| chr1_3555 | ORF3137 | 2.54E-01 | 5.284415467 | 0.019436761 |
| chr1_3473 | ORF3137 | 2.54E-01 | 2.056329157 | 0.265105431 |
| chr1_3081 | ORF3137 | 2.53E-01 | 1.479700158 | 0.144168181 |
| chr1_441 | ORF3137 | 2.52E-01 | 1.68082071 | 0.070900961 |
| chr1_2012 | ORF3137 | 2.52E-01 | 1.557058258 | 0.114675898 |
| chr1_2652 | ORF3137 | 2.52E-01 | 1.994795325 | 0.282636677 |
| chr1_540 | ORF3137 | 2.52E-01 | 1.394526838 | 0.163899264 |
| plasmid1_70 | ORF3137 | 2.51E-01 | 5.394327495 | 2.25855E-12 |
| chr1_1452 | ORF3137 | 2.51E-01 | -1.507803743 | 0.124463425 |
| plasmid3_40 | ORF3137 | 2.50E-01 | 1.231651877 | 0.238858058 |
| chr1_3019 | ORF3137 | 2.50E-01 | 1.488412886 | 0.237219717 |
| chr1_374 | ORF3137 | 2.50E-01 | 1.28597539 | 0.23447671 |
| chr1_1457 | ORF3137 | 2.50E-01 | 1.604120612 | 0.100187022 |
| chr1_2208 | ORF3137 | 2.50E-01 | -1.598025288 | 0.09193855 |
| chr2_315 | ORF3137 | 2.49E-01 | -3.825980636 | 0.007686707 |
| chr1_48 | ORF3137 | 2.48E-01 | 2.267484833 | 0.169232214 |
| chr1_222 | ORF3137 | 2.48E-01 | 1.351809044 | 0.217411391 |
| chr2_84 | ORF3137 | 2.48E-01 | -1.830836175 | 0.049021704 |
| chr1_1466 | ORF3137 | 2.48E-01 | 2.508493315 | 0.019436761 |
| chr1_2982 | ORF3137 | 2.48E-01 | 4.12461328 | 0.005847313 |
| chr1_2022 | ORF3137 | 2.48E-01 | 1.257942514 | 0.252945024 |
| chr1_2637 | ORF3137 | 2.47E-01 | 1.311959285 | 0.216457239 |
| chr1_2772 | ORF3137 | 2.47E-01 | 3.111976211 | 0.180592646 |
| chr1_1233 | ORF3137 | 2.47E-01 | 1.801049495 | 0.183238068 |
| chr1_2064 | ORF3137 | 2.47E-01 | -1.209448166 | 0.264472356 |
| chr1_1104 | ORF3137 | 2.46E-01 | 2.0194804 | 0.064220202 |
| chr1_467 | ORF3137 | 2.46E-01 | 2.711097069 | 0.000743199 |
| chr1_1359 | ORF3137 | 2.46E-01 | 3.012791465 | 0.21112227 |
| chr2_1087 | ORF3137 | 2.46E-01 | -1.637072171 | 0.085331417 |
| plasmid1_95 | ORF3137 | 2.46E-01 | 2.649612436 | 0.060529209 |
| chr1_1512 | ORF3137 | 2.45E-01 | 1.174606203 | 0.300280568 |
| chr1_1649 | ORF3137 | 2.45E-01 | -1.211936465 | 0.259787588 |
| chr2_377 | ORF3137 | 2.45E-01 | 2.765047359 | 0.259658485 |
| chr1_2967 | ORF3137 | 2.44E-01 | 1.316477537 | 0.217411391 |
| chr1_2987 | ORF3137 | 2.44E-01 | 1.263925634 | 0.24728558 |
| plasmid1_159 | ORF3137 | 2.44E-01 | -1.636051356 | 0.083038697 |
| chr2_83 | ORF3137 | 2.44E-01 | -1.8737128 | 0.039703177 |
| plasmid1_121 | ORF3137 | 2.44E-01 | 3.085216702 | 6.88413E-05 |
| chr2_383 | ORF3137 | 2.43E-01 | 3.268517915 | 0.144089078 |
| chr1_654 | ORF3137 | 2.43E-01 | 1.396305928 | 0.271205241 |
| chr1_1245 | ORF3137 | 2.43E-01 | 1.381009171 | 0.188708343 |
| chr2_961 | ORF3137 | 2.43E-01 | -1.335695937 | 0.196764159 |
| chr1_2018 | ORF3137 | 2.43E-01 | 1.608748519 | 0.196764159 |
| chr1_2600 | ORF3137 | 2.43E-01 | 1.754689108 | 0.061087032 |
| chr1_2501 | ORF3137 | 2.42E-01 | 1.20857804 | 0.277598588 |
| plasmid1_90 | ORF3137 | 2.42E-01 | 3.123312537 | 0.231681021 |
| chr1_528 | ORF3137 | 2.42E-01 | 1.707519111 | 0.083955107 |
| chr2_1009 | ORF3137 | 2.42E-01 | -1.304526692 | 0.238883099 |
| chr1_1235 | ORF3137 | 2.42E-01 | 1.513463578 | 0.18146864 |
| chr1_2549 | ORF3137 | 2.42E-01 | 2.127882647 | 0.013074217 |
| chr1_2084 | ORF3137 | 2.42E-01 | 3.771339026 | 1.15417E-05 |
| chr1_439 | ORF3137 | 2.42E-01 | 3.906677182 | 0.049086095 |
| chr1_1105 | ORF3137 | 2.42E-01 | 1.842758097 | 0.296580193 |
| chr1_2061 | ORF3137 | 2.41E-01 | 1.713457629 | 0.065475712 |
| chr2_1118 | ORF3137 | 2.41E-01 | -1.357246091 | 0.196764159 |
| chr2_960 | ORF3137 | 2.41E-01 | -1.167267203 | 0.283443927 |
| chr2_488 | ORF3137 | 2.41E-01 | 1.363910566 | 0.196764159 |
| chr1_2740 | ORF3137 | 2.41E-01 | 1.915447095 | 0.062305952 |
| chr1_2319 | ORF3137 | 2.40E-01 | 1.683486896 | 0.075656312 |
| chr2_1117 | ORF3137 | 2.40E-01 | -1.222722659 | 0.271205241 |
| plasmid1_75 | ORF3137 | 2.40E-01 | 4.294087103 | 1.29531E-08 |
| chr1_3007 | ORF3137 | 2.40E-01 | 2.46324301 | 0.020608478 |
| chr1_320 | ORF3137 | 2.40E-01 | 1.65242617 | 0.28106833 |
| chr1_1317 | ORF3137 | 2.40E-01 | 1.790811229 | 0.196764159 |
| chr1_3104 | ORF3137 | 2.39E-01 | -1.994059972 | 0.018447193 |
| chr1_2394 | ORF3137 | 2.39E-01 | 3.501540705 | 0.114675898 |
| plasmid1_52 | ORF3137 | 2.39E-01 | 3.591327636 | 2.61396E-06 |
| chr1_1 | ORF3137 | 2.39E-01 | 2.082783304 | 0.230100063 |
| plasmid1_73 | ORF3137 | 2.39E-01 | 5.274191252 | 4.48929E-12 |
| chr1_2771 | ORF3137 | 2.38E-01 | 2.596230715 | 0.227068263 |
| chr1_641 | ORF3137 | 2.38E-01 | -2.098203342 | 0.010907854 |
| plasmid1_65 | ORF3137 | 2.38E-01 | 5.814279968 | 1.11167E-13 |
| chr1_490 | ORF3137 | 2.38E-01 | 4.379733212 | 0.139152133 |
| chr2_574 | ORF3137 | 2.38E-01 | -1.485258668 | 0.145042839 |
| chr1_3267 | ORF3137 | 2.37E-01 | 2.974215127 | 0.000162728 |
| plasmid1_96 | ORF3137 | 2.37E-01 | 2.413328387 | 0.150366342 |
| chr2_1039 | ORF3137 | 2.37E-01 | -1.617515962 | 0.096129699 |
| chr1_2317 | ORF3137 | 2.37E-01 | 1.353847715 | 0.212703054 |
| chr1_199 | ORF3137 | 2.36E-01 | 2.206808756 | 0.009290103 |
| chr1_2145 | ORF3137 | 2.36E-01 | 1.207599545 | 0.28106833 |
| chr2_319 | ORF3137 | 2.36E-01 | 2.335861545 | 0.221878765 |
| chr2_617 | ORF3137 | 2.36E-01 | -3.269359441 | 1.65901E-05 |
| plasmid1_66 | ORF3137 | 2.36E-01 | 5.517173809 | 9.43962E-13 |
| chr2_637 | ORF3137 | 2.36E-01 | 2.856520852 | 0.002537821 |
| chr1_1892 | ORF3137 | 2.35E-01 | 1.871757289 | 0.041279952 |
| chr2_538 | ORF3137 | 2.35E-01 | 5.450772943 | 0.020022857 |
| chr1_2228 | ORF3137 | 2.35E-01 | 2.170754483 | 0.155824994 |
| chr1_1445 | ORF3137 | 2.35E-01 | 1.588697749 | 0.107161379 |
| chr2_1100 | ORF3137 | 2.35E-01 | -1.801262138 | 0.052752637 |
| chr1_3160 | ORF3137 | 2.35E-01 | -1.397802949 | 0.16902602 |
| plasmid1_83 | ORF3137 | 2.33E-01 | 5.019508762 | 0.000318594 |
| chr1_1869 | ORF3137 | 2.33E-01 | 1.765369554 | 0.057596726 |
| chr1_696 | ORF3137 | 2.33E-01 | 1.765686894 | 0.056595459 |
| chr1_10 | ORF3137 | 2.33E-01 | 1.746600499 | 0.175289513 |
| chr1_1191 | ORF3137 | 2.33E-01 | -1.280974436 | 0.212703054 |
| chr1_2131 | ORF3137 | 2.33E-01 | 1.259466375 | 0.262546754 |
| plasmid2_27 | ORF3137 | 2.32E-01 | -1.416697651 | 0.161428647 |
| chr1_2985 | ORF3137 | 2.32E-01 | 3.643834503 | 0.100769269 |
| chr2_1101 | ORF3137 | 2.32E-01 | -1.547891795 | 0.115229856 |
| chr1_1241 | ORF3137 | 2.32E-01 | 1.374938057 | 0.196764159 |
| chr1_3563 | ORF3137 | 2.32E-01 | 4.191995411 | 0.073235374 |
| plasmid1_239 | ORF3137 | 2.31E-01 | -1.484088561 | 0.147819153 |
| chr1_2565 | ORF3137 | 2.30E-01 | 1.262649057 | 0.245657954 |
| chr1_2059 | ORF3137 | 2.30E-01 | 1.946178764 | 0.169232214 |
| chr1_2130 | ORF3137 | 2.30E-01 | 1.730536048 | 0.114349314 |
| chr1_1485 | ORF3137 | 2.29E-01 | 1.341734979 | 0.207612726 |
| chr1_2415 | ORF3137 | 2.28E-01 | 1.55619922 | 0.218277413 |
| chr1_2959 | ORF3137 | 2.28E-01 | -1.454556776 | 0.1351218 |
| chr1_255 | ORF3137 | 2.28E-01 | 2.7213608 | 0.000742129 |
| chr1_3397 | ORF3137 | 2.28E-01 | 3.182429014 | 0.018447193 |
| chr1_2577 | ORF3137 | 2.27E-01 | 6.873078756 | 0.010808573 |
| chr1_2593 | ORF3137 | 2.27E-01 | 1.420797866 | 0.300405059 |
| chr2_955 | ORF3137 | 2.27E-01 | -1.32448917 | 0.203499618 |
| chr1_1175 | ORF3137 | 2.27E-01 | 1.494746533 | 0.142483925 |
| chr1_256 | ORF3137 | 2.27E-01 | 3.067914285 | 8.34966E-05 |
| chr2_1098 | ORF3137 | 2.27E-01 | -1.466790932 | 0.155824994 |
| chr2_449 | ORF3137 | 2.27E-01 | -1.351733558 | 0.231950971 |
| chr1_260 | ORF3137 | 2.27E-01 | 1.410403675 | 0.170396541 |
| plasmid1_71 | ORF3137 | 2.26E-01 | 5.548619996 | 8.11895E-13 |
| chr1_2674 | ORF3137 | 2.26E-01 | 1.242597801 | 0.260553508 |
| plasmid1_80 | ORF3137 | 2.26E-01 | 5.292391104 | 4.65531E-12 |
| chr1_1873 | ORF3137 | 2.26E-01 | 1.458498895 | 0.150361795 |
| chr2_927 | ORF3137 | 2.26E-01 | 1.989942944 | 0.171992016 |
| chr1_2355 | ORF3137 | 2.25E-01 | 1.851647578 | 0.281354716 |
| chr1_2338 | ORF3137 | 2.25E-01 | 6.152106907 | 0.008548485 |
| chr1_1773 | ORF3137 | 2.25E-01 | 1.85239286 | 0.043756264 |
| plasmid1_116 | ORF3137 | 2.24E-01 | 3.446471571 | 5.9699E-06 |
| chr2_480 | ORF3137 | 2.24E-01 | 2.206185479 | 0.153935512 |
| chr2_291 | ORF3137 | 2.23E-01 | 1.42643882 | 0.183238068 |
| chr2_954 | ORF3137 | 2.23E-01 | -1.172837699 | 0.296580193 |
| chr1_354 | ORF3137 | 2.23E-01 | 2.591269832 | 0.00149881 |
| chr1_2157 | ORF3137 | 2.23E-01 | 1.393660185 | 0.213749056 |
| plasmid1_115 | ORF3137 | 2.23E-01 | 3.392302863 | 1.0478E-05 |
| chr2_624 | ORF3137 | 2.23E-01 | -1.325206177 | 0.220659929 |
| chr1_1982 | ORF3137 | 2.23E-01 | 3.817462125 | 0.042122491 |
| chr1_462 | ORF3137 | 2.23E-01 | 1.623178302 | 0.089988267 |
| plasmid1_118 | ORF3137 | 2.22E-01 | 3.397273001 | 7.54487E-06 |
| chr1_1775 | ORF3137 | 2.22E-01 | 2.366823164 | 0.005436674 |
| chr2_614 | ORF3137 | 2.22E-01 | -1.303618066 | 0.239531092 |
| chr1_535 | ORF3137 | 2.21E-01 | 1.2413611 | 0.258992582 |
| chr1_1789 | ORF3137 | 2.21E-01 | 2.645047277 | 0.08683018 |
| chr1_3011 | ORF3137 | 2.21E-01 | 2.304265237 | 0.006022746 |
| chr1_274 | ORF3137 | 2.21E-01 | 2.850867411 | 0.286022312 |
| chr1_1851 | ORF3137 | 2.20E-01 | 2.657979662 | 0.248195731 |
| chr1_2086 | ORF3137 | 2.20E-01 | 4.110731961 | 4.5888E-08 |
| plasmid1_111 | ORF3137 | 2.20E-01 | 3.548513461 | 0.096129699 |
| chr1_1084 | ORF3137 | 2.20E-01 | 1.434220204 | 0.165528896 |
| chr1_2818 | ORF3137 | 2.19E-01 | -1.26893861 | 0.222803256 |
| chr1_1318 | ORF3137 | 2.19E-01 | 2.037932042 | 0.248195731 |
| chr1_2389 | ORF3137 | 2.19E-01 | 3.220545395 | 0.1351218 |
| chr2_1133 | ORF3137 | 2.19E-01 | 4.695104257 | 0.027560334 |
| chr1_2025 | ORF3137 | 2.19E-01 | 2.466323363 | 0.002720949 |
| chr2_921 | ORF3137 | 2.18E-01 | -1.211258851 | 0.2625768 |
| chr1_3269 | ORF3137 | 2.18E-01 | 2.779106431 | 0.000594584 |
| chr1_2503 | ORF3137 | 2.18E-01 | 2.468961758 | 0.224120934 |
| chr1_14 | ORF3137 | 2.18E-01 | 1.290345524 | 0.237219717 |
| chr2_1085 | ORF3137 | 2.17E-01 | -1.268910879 | 0.243490125 |
| chr2_1083 | ORF3137 | 2.16E-01 | -1.247688403 | 0.259787588 |
| chr1_1122 | ORF3137 | 2.16E-01 | 3.243146321 | 0.163899264 |
| chr1_1983 | ORF3137 | 2.16E-01 | 2.242208653 | 0.170395906 |
| plasmid1_110 | ORF3137 | 2.16E-01 | 4.071966484 | 0.061087032 |
| chr2_1056 | ORF3137 | 2.16E-01 | -1.336658931 | 0.196764159 |
| plasmid1_72 | ORF3137 | 2.16E-01 | 5.922343478 | 7.09884E-14 |
| plasmid1_123 | ORF3137 | 2.15E-01 | 5.807109557 | 1.11167E-13 |
| chr1_1764 | ORF3137 | 2.14E-01 | 3.059587256 | 0.071605117 |
| plasmid1_113 | ORF3137 | 2.14E-01 | 2.289246347 | 0.265236476 |
| chr1_529 | ORF3137 | 2.14E-01 | 1.717758337 | 0.062698068 |
| chr2_891 | ORF3137 | 2.14E-01 | 3.774101891 | 0.202441314 |
| chr1_636 | ORF3137 | 2.14E-01 | 2.181668722 | 0.200961184 |
| chr1_1238 | ORF3137 | 2.14E-01 | 1.313666313 | 0.227068263 |
| chr1_1771 | ORF3137 | 2.13E-01 | 2.544141196 | 0.002190228 |
| plasmid1_112 | ORF3137 | 2.13E-01 | 1.922944498 | 0.28177137 |
| chr2_356 | ORF3137 | 2.13E-01 | 9.256123301 | 0.00487524 |
| chr1_1288 | ORF3137 | 2.13E-01 | 2.27378245 | 0.007394775 |
| plasmid1_150 | ORF3137 | 2.12E-01 | -2.352364539 | 0.004809784 |
| chr1_19 | ORF3137 | 2.12E-01 | 2.951580226 | 0.05368412 |
| chr1_461 | ORF3137 | 2.12E-01 | 1.869656816 | 0.038738407 |
| chr1_1748 | ORF3137 | 2.12E-01 | 1.868055652 | 0.040620295 |
| chr1_218 | ORF3137 | 2.12E-01 | 2.236194166 | 0.00950286 |
| chr1_1272 | ORF3137 | 2.11E-01 | 1.664517547 | 0.08397354 |
| chr1_3564 | ORF3137 | 2.11E-01 | 2.090119154 | 0.016905664 |
| chr2_312 | ORF3137 | 2.11E-01 | -1.704991188 | 0.205687042 |
| chr1_537 | ORF3137 | 2.11E-01 | 1.692138886 | 0.064220202 |
| chr1_2502 | ORF3137 | 2.11E-01 | 1.520173319 | 0.121978679 |
| chr1_1228 | ORF3137 | 2.10E-01 | 1.947838521 | 0.027616898 |
| chr1_3192 | ORF3137 | 2.10E-01 | -1.24928502 | 0.259787588 |
| chr1_2248 | ORF3137 | 2.10E-01 | 1.48203737 | 0.142864037 |
| chr1_2574 | ORF3137 | 2.10E-01 | 1.430241925 | 0.182822529 |
| chr1_2639 | ORF3137 | 2.09E-01 | 1.63184119 | 0.106205274 |
| plasmid1_76 | ORF3137 | 2.09E-01 | 4.164250175 | 0.006859109 |
| chr1_1126 | ORF3137 | 2.09E-01 | 1.849474685 | 0.282636677 |
| chr1_1286 | ORF3137 | 2.09E-01 | 2.062597808 | 0.234528103 |
| chr1_2477 | ORF3137 | 2.09E-01 | 1.578955178 | 0.110321349 |
| chr2_283 | ORF3137 | 2.09E-01 | -1.223606309 | 0.265105431 |
| plasmid1_105 | ORF3137 | 2.09E-01 | 1.719871318 | 0.066494886 |
| chr1_2386 | ORF3137 | 2.08E-01 | 2.402669805 | 0.14420261 |
| chr1_3238 | ORF3137 | 2.08E-01 | 1.6545034 | 0.143835066 |
| plasmid1_106 | ORF3137 | 2.08E-01 | 2.467902507 | 0.002720949 |
| chr1_1819 | ORF3137 | 2.07E-01 | 4.629484847 | 0.093634739 |
| chr1_3533 | ORF3137 | 2.07E-01 | 1.914445635 | 0.03264265 |
| chr2_934 | ORF3137 | 2.07E-01 | -1.316024485 | 0.194777416 |
| chr1_46 | ORF3137 | 2.07E-01 | 2.478900978 | 0.012760001 |
| chr1_2711 | ORF3137 | 2.07E-01 | -1.591746141 | 0.101992691 |
| chr1_1186 | ORF3137 | 2.06E-01 | 1.556477859 | 0.114675898 |
| chr1_605 | ORF3137 | 2.06E-01 | 2.843403912 | 0.000332051 |
| chr1_40 | ORF3137 | 2.06E-01 | 1.214778841 | 0.272910517 |
| chr1_2688 | ORF3137 | 2.06E-01 | 1.21921628 | 0.291496774 |
| chr1_2895 | ORF3137 | 2.05E-01 | 2.668853339 | 0.148485823 |
| chr2_504 | ORF3137 | 2.05E-01 | 7.149704628 | 0.026714553 |
| chr2_252 | ORF3137 | 2.05E-01 | -1.396171012 | 0.223474929 |
| chr1_101 | ORF3137 | 2.05E-01 | 2.211727467 | 0.009963934 |
| chr1_1107 | ORF3137 | 2.04E-01 | 1.265427207 | 0.252945024 |
| chr2_776 | ORF3137 | 2.04E-01 | -1.375122509 | 0.180592646 |
| chr1_653 | ORF3137 | 2.04E-01 | 1.913893661 | 0.161354443 |
| chr1_1106 | ORF3137 | 2.04E-01 | 1.636510959 | 0.21112227 |
| chr1_807 | ORF3137 | 2.03E-01 | 1.788050055 | 0.056208264 |
| chr1_172 | ORF3137 | 2.03E-01 | 2.177319625 | 0.11109673 |
| chr1_460 | ORF3137 | 2.03E-01 | 1.774294741 | 0.063977106 |
| chr2_472 | ORF3137 | 2.03E-01 | 1.314558438 | 0.212854158 |
| chr1_3399 | ORF3137 | 2.02E-01 | 1.42422042 | 0.158054707 |
| chr1_2975 | ORF3137 | 2.02E-01 | 1.818766966 | 0.114675898 |
| chr1_1275 | ORF3137 | 2.01E-01 | 2.271558729 | 0.196764159 |
| chr1_468 | ORF3137 | 2.01E-01 | 2.314585867 | 0.064220202 |
| chr2_1161 | ORF3137 | 2.01E-01 | -1.269552707 | 0.235847471 |
| plasmid1_84 | ORF3137 | 2.00E-01 | 5.197479634 | 9.67536E-07 |
| chr1_3472 | ORF3137 | 2.00E-01 | 1.386459179 | 0.196764159 |
| chr1_2760 | ORF3137 | 2.00E-01 | -1.345941444 | 0.237775422 |
| chr1_2462 | ORF3137 | 2.00E-01 | -1.3315547 | 0.196764159 |
| chr2_1081 | ORF3137 | 2.00E-01 | -2.164145093 | 0.013793675 |
| chr1_1121 | ORF3137 | 1.99E-01 | 3.26375416 | 0.219591444 |
| chr2_681 | ORF3137 | 1.99E-01 | -1.292077297 | 0.196814598 |
| chr1_1311 | ORF3137 | 1.98E-01 | 1.31781271 | 0.214821393 |
| chr1_2699 | ORF3137 | 1.98E-01 | 1.53385749 | 0.132137795 |
| chr1_2226 | ORF3137 | 1.97E-01 | 2.370514164 | 0.281600812 |
| chr1_459 | ORF3137 | 1.97E-01 | 1.854517271 | 0.042122491 |
| chr1_2246 | ORF3137 | 1.97E-01 | 1.616963564 | 0.103519566 |
| chr2_1075 | ORF3137 | 1.97E-01 | -1.566132232 | 0.114349314 |
| chr1_527 | ORF3137 | 1.96E-01 | 1.181560611 | 0.275252448 |
| chr1_386 | ORF3137 | 1.96E-01 | 1.333729611 | 0.22582035 |
| chr1_1562 | ORF3137 | 1.96E-01 | -1.166751619 | 0.277892413 |
| chr2_775 | ORF3137 | 1.96E-01 | -1.473541567 | 0.129967853 |
| chr1_806 | ORF3137 | 1.96E-01 | 1.714374183 | 0.070387807 |
| chr1_3395 | ORF3137 | 1.96E-01 | 1.680443601 | 0.082728216 |
| chr1_3416 | ORF3137 | 1.95E-01 | -1.15736329 | 0.269924678 |
| plasmid1_69 | ORF3137 | 1.95E-01 | 5.798274797 | 1.11167E-13 |
| chr1_1799 | ORF3137 | 1.94E-01 | 1.924658641 | 0.122897539 |
| chr1_2116 | ORF3137 | 1.94E-01 | 1.598890407 | 0.300280568 |
| chr1_3398 | ORF3137 | 1.94E-01 | 1.789608572 | 0.055052679 |
| chr1_538 | ORF3137 | 1.94E-01 | 1.622772495 | 0.083955107 |
| chr2_615 | ORF3137 | 1.94E-01 | -3.134013239 | 7.11157E-05 |
| chr1_2391 | ORF3137 | 1.94E-01 | 3.448472213 | 0.268582449 |
| chr1_1294 | ORF3137 | 1.93E-01 | 3.203143429 | 0.062242933 |
| chr1_2515 | ORF3137 | 1.93E-01 | 1.238922421 | 0.265527113 |
| chr1_56 | ORF3137 | 1.93E-01 | 2.519269963 | 0.24728558 |
| plasmid1_48 | ORF3137 | 1.92E-01 | 4.906033932 | 1.28401E-10 |
| chr2_18 | ORF3137 | 1.92E-01 | 1.630584464 | 0.237219717 |
| plasmid1_149 | ORF3137 | 1.92E-01 | -1.415170356 | 0.180450111 |
| chr1_1909 | ORF3137 | 1.92E-01 | 3.957216046 | 0.130361343 |
| chr1_1380 | ORF3137 | 1.92E-01 | 1.492544608 | 0.196764159 |
| chr1_3023 | ORF3137 | 1.92E-01 | 1.329865603 | 0.218375053 |
| chr2_544 | ORF3137 | 1.92E-01 | 3.920922571 | 0.104818827 |
| chr1_3270 | ORF3137 | 1.92E-01 | 2.395442138 | 0.045582027 |
| chr1_1358 | ORF3137 | 1.90E-01 | 1.650096241 | 0.163899264 |
| chr1_3532 | ORF3137 | 1.90E-01 | 1.720800697 | 0.062305952 |
| chr1_190 | ORF3137 | 1.89E-01 | 1.485551676 | 0.280982772 |
| chr1_2087 | ORF3137 | 1.88E-01 | 2.80191734 | 0.001845005 |
| chr1_3240 | ORF3137 | 1.88E-01 | 1.810859473 | 0.107161379 |
| chr1_2099 | ORF3137 | 1.87E-01 | 1.789365399 | 0.196764159 |
| chr1_2423 | ORF3137 | 1.86E-01 | 1.431983117 | 0.174130718 |
| chr1_2968 | ORF3137 | 1.86E-01 | 1.664959224 | 0.087935145 |
| chr1_416 | ORF3137 | 1.85E-01 | 6.347976361 | 0.031034584 |
| chr1_3231 | ORF3137 | 1.85E-01 | 1.432060003 | 0.180592646 |
| chr2_739 | ORF3137 | 1.85E-01 | 3.954042875 | 0.211672565 |
| chr1_1413 | ORF3137 | 1.85E-01 | 2.303131026 | 0.007041248 |
| chr1_2962 | ORF3137 | 1.84E-01 | 1.881647483 | 0.291496774 |
| chr1_3010 | ORF3137 | 1.84E-01 | 2.571169693 | 0.00149881 |
| chr1_465 | ORF3137 | 1.84E-01 | 1.314669669 | 0.279028294 |
| chr1_2380 | ORF3137 | 1.84E-01 | 3.213242905 | 0.235410847 |
| chr1_697 | ORF3137 | 1.84E-01 | 4.291434305 | 0.02473178 |
| chr1_1774 | ORF3137 | 1.84E-01 | 1.558919617 | 0.124449656 |
| chr1_709 | ORF3137 | 1.84E-01 | 4.59019851 | 0.055919671 |
| chr1_2514 | ORF3137 | 1.84E-01 | 1.79481278 | 0.265216149 |
| chr1_417 | ORF3137 | 1.83E-01 | 8.971357854 | 0.001286818 |
| chr1_1882 | ORF3137 | 1.83E-01 | 2.022008255 | 0.218375053 |
| chr1_1295 | ORF3137 | 1.83E-01 | 1.968112929 | 0.028316054 |
| chr2_629 | ORF3137 | 1.83E-01 | -1.418054768 | 0.146766302 |
| chr1_2375 | ORF3137 | 1.83E-01 | 3.15874926 | 0.073675513 |
| chr1_2085 | ORF3137 | 1.83E-01 | 4.329285292 | 7.23296E-06 |
| chr1_1446 | ORF3137 | 1.83E-01 | 1.303869321 | 0.232679958 |
| chr1_2984 | ORF3137 | 1.82E-01 | 3.867413438 | 0.067404078 |
| chr1_2019 | ORF3137 | 1.82E-01 | 1.755028968 | 0.065396712 |
| plasmid1_77 | ORF3137 | 1.82E-01 | 6.641737844 | 4.54668E-16 |
| chr1_1787 | ORF3137 | 1.82E-01 | 3.96472932 | 0.009963934 |
| chr2_700 | ORF3137 | 1.81E-01 | -1.496943285 | 0.150199974 |
| chr1_854 | ORF3137 | 1.81E-01 | 1.392145636 | 0.200440457 |
| chr1_1744 | ORF3137 | 1.81E-01 | -1.36544745 | 0.196764159 |
| chr1_435 | ORF3137 | 1.81E-01 | 3.096908242 | 0.046787003 |
| chr1_1310 | ORF3137 | 1.81E-01 | 1.260892837 | 0.241934321 |
| chr1_1076 | ORF3137 | 1.80E-01 | 1.232253471 | 0.271252695 |
| chr1_3119 | ORF3137 | 1.80E-01 | 2.442002597 | 0.271205241 |
| chr1_3444 | ORF3137 | 1.80E-01 | 3.166356084 | 0.155824994 |
| chr1_103 | ORF3137 | 1.80E-01 | 1.990190361 | 0.231950971 |
| chr1_1928 | ORF3137 | 1.80E-01 | -1.231243798 | 0.294246691 |
| chr1_2981 | ORF3137 | 1.80E-01 | 6.591275864 | 2.1023E-12 |
| chr1_380 | ORF3137 | 1.79E-01 | 1.750425975 | 0.267181035 |
| chr1_221 | ORF3137 | 1.79E-01 | 2.577356905 | 0.170183978 |
| chr1_2716 | ORF3137 | 1.78E-01 | 4.769362655 | 0.196764159 |
| chr1_329 | ORF3137 | 1.78E-01 | 2.155653692 | 0.237087679 |
| chr1_850 | ORF3137 | 1.78E-01 | 1.556789847 | 0.196764159 |
| chr2_986 | ORF3137 | 1.78E-01 | -1.439446458 | 0.1536535 |
| chr1_1788 | ORF3137 | 1.78E-01 | 3.72291526 | 0.004145917 |
| chr2_1092 | ORF3137 | 1.78E-01 | 1.891707062 | 0.276220023 |
| plasmid1_93 | ORF3137 | 1.77E-01 | 2.224305414 | 0.025956293 |
| chr1_1335 | ORF3137 | 1.77E-01 | 1.285836162 | 0.243898505 |
| chr1_359 | ORF3137 | 1.77E-01 | 2.171998566 | 0.214821393 |
| chr1_2297 | ORF3137 | 1.77E-01 | 1.640388554 | 0.087935145 |
| chr1_242 | ORF3137 | 1.77E-01 | -1.444640873 | 0.163899264 |
| plasmid1_126 | ORF3137 | 1.77E-01 | 5.053127994 | 6.59163E-05 |
| chr1_3027 | ORF3137 | 1.77E-01 | 2.584151316 | 0.001547283 |
| chr2_796 | ORF3137 | 1.77E-01 | 2.572613806 | 0.001583445 |
| plasmid1_243 | ORF3137 | 1.77E-01 | 1.210706694 | 0.272910517 |
| plasmid1_87 | ORF3137 | 1.77E-01 | 6.040117913 | 5.03792E-14 |
| chr1_603 | ORF3137 | 1.76E-01 | 7.215730064 | 0.010174644 |
| chr1_2702 | ORF3137 | 1.76E-01 | 2.037274978 | 0.1757674 |
| chr2_378 | ORF3137 | 1.76E-01 | 5.190634874 | 0.064064233 |
| chr1_1285 | ORF3137 | 1.76E-01 | 1.70747577 | 0.109532181 |
| chr1_219 | ORF3137 | 1.75E-01 | 2.081201683 | 0.033561072 |
| chr1_3089 | ORF3137 | 1.75E-01 | 1.448284738 | 0.163899264 |
| chr1_625 | ORF3137 | 1.75E-01 | 2.160297056 | 0.01269233 |
| chr1_1461 | ORF3137 | 1.75E-01 | 1.790521864 | 0.295975268 |
| chr1_770 | ORF3137 | 1.74E-01 | 2.753810474 | 0.000707148 |
| chr1_2719 | ORF3137 | 1.74E-01 | 2.443558487 | 0.003694929 |
| plasmid1_92 | ORF3137 | 1.74E-01 | 2.819681437 | 0.000438445 |
| chr1_3396 | ORF3137 | 1.73E-01 | 2.342711841 | 0.007781545 |
| chr2_129 | ORF3137 | 1.73E-01 | -2.116403928 | 0.012286335 |
| plasmid1_49 | ORF3137 | 1.73E-01 | 4.544201756 | 1.84452E-09 |
| chr1_2115 | ORF3137 | 1.73E-01 | 1.385191134 | 0.277892413 |
| chr1_2342 | ORF3137 | 1.73E-01 | -1.428133438 | 0.153408779 |
| chr1_3556 | ORF3137 | 1.72E-01 | 6.182477703 | 0.026714553 |
| chr1_1918 | ORF3137 | 1.72E-01 | -4.389919403 | 3.06109E-09 |
| plasmid1_158 | ORF3137 | 1.71E-01 | -1.885465289 | 0.029861748 |
| chr1_227 | ORF3137 | 1.71E-01 | 2.536660989 | 0.256956753 |
| chr2_453 | ORF3137 | 1.70E-01 | -1.796752507 | 0.050199766 |
| chr1_2296 | ORF3137 | 1.70E-01 | 1.736391871 | 0.06690536 |
| chr1_1357 | ORF3137 | 1.70E-01 | 2.455889595 | 0.058231811 |
| plasmid1_74 | ORF3137 | 1.69E-01 | 4.521731321 | 2.40701E-09 |
| chr1_1379 | ORF3137 | 1.69E-01 | 1.733499375 | 0.087935145 |
| chr2_930 | ORF3137 | 1.69E-01 | 2.849164008 | 0.00149881 |
| chr1_1531 | ORF3137 | 1.68E-01 | 2.378517368 | 0.234860921 |
| chr2_318 | ORF3137 | 1.68E-01 | 4.201423153 | 0.143890809 |
| chr1_1344 | ORF3137 | 1.68E-01 | 1.308607254 | 0.238324058 |
| plasmid1_119 | ORF3137 | 1.68E-01 | 2.281482368 | 0.00955364 |
| chr1_1651 | ORF3137 | 1.67E-01 | -1.216309775 | 0.295109017 |
| plasmid1_146 | ORF3137 | 1.67E-01 | 1.260651717 | 0.24080612 |
| chr1_2550 | ORF3137 | 1.67E-01 | 3.409913586 | 0.136232905 |
| chr1_2990 | ORF3137 | 1.66E-01 | 1.544379158 | 0.291496774 |
| chr2_1066 | ORF3137 | 1.66E-01 | -1.861544836 | 0.056304368 |
| chr1_2697 | ORF3137 | 1.66E-01 | 2.206098909 | 0.009290103 |
| chr1_1176 | ORF3137 | 1.66E-01 | 3.440432765 | 0.114675898 |
| chr1_2681 | ORF3137 | 1.65E-01 | 1.180624377 | 0.300280568 |
| chr1_3254 | ORF3137 | 1.65E-01 | -2.127335807 | 0.011182224 |
| chr1_55 | ORF3137 | 1.65E-01 | 3.200097432 | 0.114675898 |
| chr1_1319 | ORF3137 | 1.65E-01 | 3.183454033 | 0.163899264 |
| chr1_2739 | ORF3137 | 1.65E-01 | -1.270564533 | 0.227068263 |
| chr1_1797 | ORF3137 | 1.65E-01 | 1.220985394 | 0.259787588 |
| chr1_253 | ORF3137 | 1.64E-01 | 1.263373038 | 0.291496774 |
| plasmid1_218 | ORF3137 | 1.64E-01 | -1.190325354 | 0.268582449 |
| chr1_3006 | ORF3137 | 1.64E-01 | 3.667126745 | 0.000743199 |
| chr1_47 | ORF3137 | 1.64E-01 | 2.788864087 | 0.012286335 |
| chr1_475 | ORF3137 | 1.63E-01 | 3.352408097 | 0.014425288 |
| chr1_2677 | ORF3137 | 1.63E-01 | 2.034610383 | 0.02473178 |
| chr1_2988 | ORF3137 | 1.63E-01 | 1.369692918 | 0.196292526 |
| chr2_1099 | ORF3137 | 1.63E-01 | -1.595964654 | 0.144078549 |
| chr2_407 | ORF3137 | 1.63E-01 | 4.639524254 | 8.80777E-10 |
| plasmid1_81 | ORF3137 | 1.62E-01 | 5.373688082 | 3.42549E-12 |
| plasmid1_236 | ORF3137 | 1.62E-01 | 2.236772892 | 0.268582449 |
| chr1_839 | ORF3137 | 1.62E-01 | 3.011944993 | 0.25933684 |
| chr1_3009 | ORF3137 | 1.62E-01 | 2.654657213 | 0.006452988 |
| chr1_1178 | ORF3137 | 1.62E-01 | 1.455029694 | 0.173942465 |
| chr1_2451 | ORF3137 | 1.62E-01 | 2.146631981 | 0.165528896 |
| chr1_3159 | ORF3137 | 1.61E-01 | -1.253735286 | 0.223474929 |
| chr2_830 | ORF3137 | 1.61E-01 | -1.569007909 | 0.179557954 |
| chr1_604 | ORF3137 | 1.61E-01 | 3.290549269 | 0.060865878 |
| chr1_75 | ORF3137 | 1.61E-01 | 8.041797602 | 0.006723252 |
| chr1_1489 | ORF3137 | 1.61E-01 | 3.430466312 | 0.001055487 |
| chr1_1113 | ORF3137 | 1.60E-01 | 2.62159505 | 0.001498128 |
| plasmid1_78 | ORF3137 | 1.59E-01 | 5.068779528 | 4.71386E-11 |
| chr2_942 | ORF3137 | 1.59E-01 | -1.319863674 | 0.251746403 |
| chr2_188 | ORF3137 | 1.59E-01 | 1.197915819 | 0.291496774 |
| chr1_1519 | ORF3137 | 1.59E-01 | 3.409554512 | 0.23999962 |
| chr1_3215 | ORF3137 | 1.59E-01 | 4.826573893 | 3.1786E-05 |
| chr1_24 | ORF3137 | 1.59E-01 | 1.397717665 | 0.177244868 |
| chr1_2642 | ORF3137 | 1.59E-01 | 5.096233052 | 0.073235374 |
| chr1_3253 | ORF3137 | 1.59E-01 | -2.002836626 | 0.020022857 |
| chr1_3302 | ORF3137 | 1.58E-01 | 3.16817989 | 0.21112227 |
| plasmid1_85 | ORF3137 | 1.58E-01 | 5.185877078 | 0.006485821 |
| chr1_2050 | ORF3137 | 1.58E-01 | -1.511511951 | 0.113505809 |
| chr1_1236 | ORF3137 | 1.58E-01 | 1.646757861 | 0.170407785 |
| chr1_2553 | ORF3137 | 1.58E-01 | 3.510446812 | 0.000243006 |
| chr1_767 | ORF3137 | 1.58E-01 | 1.690142709 | 0.271205241 |
| chr1_1800 | ORF3137 | 1.58E-01 | 2.057749836 | 0.023463999 |
| chr1_3218 | ORF3137 | 1.58E-01 | 1.515813653 | 0.291496774 |
| chr1_700 | ORF3137 | 1.58E-01 | 1.313912519 | 0.265216149 |
| chr1_1750 | ORF3137 | 1.57E-01 | -2.031011061 | 0.016551431 |
| plasmid1_109 | ORF3137 | 1.57E-01 | 2.865945036 | 0.027641727 |
| chr1_562 | ORF3137 | 1.57E-01 | -1.600905622 | 0.092327074 |
| chr1_2094 | ORF3137 | 1.57E-01 | 1.570846643 | 0.142305172 |
| chr1_1627 | ORF3137 | 1.56E-01 | 2.997919989 | 0.227068263 |
| chr1_2044 | ORF3137 | 1.56E-01 | 1.924546714 | 0.119823707 |
| chr2_985 | ORF3137 | 1.56E-01 | -1.765244084 | 0.048635351 |
| chr2_311 | ORF3137 | 1.56E-01 | 1.563188691 | 0.1105904 |
| chr1_1790 | ORF3137 | 1.56E-01 | 2.981954597 | 0.002504275 |
| chr1_890 | ORF3137 | 1.55E-01 | 5.329020258 | 0.062305952 |
| chr1_3562 | ORF3137 | 1.55E-01 | 2.298982211 | 0.095199076 |
| chr1_1440 | ORF3137 | 1.55E-01 | 1.837163422 | 0.058231811 |
| chr1_3013 | ORF3137 | 1.54E-01 | 2.051903065 | 0.291496774 |
| plasmid1_133 | ORF3137 | 1.54E-01 | 2.797692491 | 0.079217703 |
| chr1_602 | ORF3137 | 1.54E-01 | 2.366944656 | 0.005847313 |
| chr1_455 | ORF3137 | 1.54E-01 | 3.055825673 | 0.122897539 |
| chr1_2676 | ORF3137 | 1.54E-01 | 1.798651747 | 0.052660493 |
| chr2_904 | ORF3137 | 1.54E-01 | -1.535229666 | 0.112163741 |
| chr1_2377 | ORF3137 | 1.53E-01 | 3.340099388 | 0.01730413 |
| chr1_2661 | ORF3137 | 1.53E-01 | -2.513506157 | 0.001583445 |
| chr2_650 | ORF3137 | 1.53E-01 | -1.681085982 | 0.200961184 |
| chr1_3008 | ORF3137 | 1.53E-01 | 2.426498793 | 0.107724963 |
| chr1_2643 | ORF3137 | 1.52E-01 | -1.295290293 | 0.232894645 |
| chr2_929 | ORF3137 | 1.52E-01 | 3.662918537 | 1.27119E-06 |
| chr1_866 | ORF3137 | 1.52E-01 | -1.304503249 | 0.276992095 |
| chr1_2692 | ORF3137 | 1.52E-01 | 3.132767096 | 0.027984483 |
| chr1_453 | ORF3137 | 1.52E-01 | 2.083096613 | 0.185361066 |
| chr1_1716 | ORF3137 | 1.52E-01 | -1.626369115 | 0.175289513 |
| chr1_383 | ORF3137 | 1.51E-01 | 2.339166168 | 0.196764159 |
| chr1_2098 | ORF3137 | 1.51E-01 | 1.665294557 | 0.197390967 |
| chr1_3394 | ORF3137 | 1.51E-01 | 2.607686087 | 0.003209891 |
| chr1_1981 | ORF3137 | 1.51E-01 | 4.649267449 | 0.068058232 |
| chr1_216 | ORF3137 | 1.51E-01 | 2.633699071 | 0.001583445 |
| chr1_3470 | ORF3137 | 1.50E-01 | -1.781435157 | 0.05206044 |
| chr2_461 | ORF3137 | 1.50E-01 | 1.46985303 | 0.147819153 |
| chr1_1893 | ORF3137 | 1.50E-01 | 2.571500731 | 0.001797618 |
| chr1_1548 | ORF3137 | 1.50E-01 | 2.850277129 | 0.003235563 |
| chr1_1439 | ORF3137 | 1.50E-01 | 1.836513971 | 0.052752637 |
| chr1_859 | ORF3137 | 1.49E-01 | 1.809403361 | 0.052952553 |
| chr1_2385 | ORF3137 | 1.49E-01 | 3.568842101 | 0.025415247 |
| chr1_3255 | ORF3137 | 1.48E-01 | -2.081291665 | 0.016902937 |
| chr1_1296 | ORF3137 | 1.47E-01 | 1.479693344 | 0.177623011 |
| chr1_630 | ORF3137 | 1.47E-01 | 2.024224416 | 0.103561706 |
| chr1_2718 | ORF3137 | 1.46E-01 | 1.71576389 | 0.100769269 |
| chr1_3209 | ORF3137 | 1.46E-01 | -1.427064575 | 0.158054707 |
| chr1_1980 | ORF3137 | 1.46E-01 | 5.114203095 | 0.028809972 |
| chr1_94 | ORF3137 | 1.46E-01 | 1.835712671 | 0.15302248 |
| chr2_839 | ORF3137 | 1.45E-01 | 3.664373072 | 0.239531092 |
| chr2_1022 | ORF3137 | 1.45E-01 | -1.408639065 | 0.193691945 |
| chr1_1793 | ORF3137 | 1.45E-01 | 1.473684156 | 0.232860622 |
| plasmid1_237 | ORF3137 | 1.44E-01 | 3.965299063 | 0.049236158 |
| chr1_171 | ORF3137 | 1.44E-01 | 1.497120481 | 0.281280664 |
| chr1_1221 | ORF3137 | 1.44E-01 | 1.319465995 | 0.216971141 |
| chr1_2401 | ORF3137 | 1.44E-01 | 3.543243861 | 0.005862937 |
| chr1_173 | ORF3137 | 1.44E-01 | 1.73876612 | 0.067873731 |
| chr1_2382 | ORF3137 | 1.44E-01 | 3.162132465 | 0.263316935 |
| chr1_2644 | ORF3137 | 1.43E-01 | -1.505318259 | 0.13782073 |
| chr1_626 | ORF3137 | 1.43E-01 | 2.660605718 | 0.006452988 |
| chr1_1356 | ORF3137 | 1.43E-01 | 1.643247625 | 0.216640809 |
| chr1_3528 | ORF3137 | 1.43E-01 | 4.179039946 | 7.97296E-08 |
| chr1_1477 | ORF3137 | 1.42E-01 | 3.019153384 | 0.150361795 |
| chr1_331 | ORF3137 | 1.42E-01 | -3.076834611 | 5.44582E-05 |
| chr1_17 | ORF3137 | 1.42E-01 | 1.614705546 | 0.107161379 |
| chr1_1438 | ORF3137 | 1.41E-01 | 1.509989727 | 0.269924678 |
| chr1_102 | ORF3137 | 1.41E-01 | 2.746266535 | 0.025415247 |
| chr1_1844 | ORF3137 | 1.41E-01 | 2.022377947 | 0.031034584 |
| chr1_891 | ORF3137 | 1.40E-01 | 7.239753682 | 0.014764887 |
| chr1_2378 | ORF3137 | 1.40E-01 | 2.61647229 | 0.127017286 |
| chr1_1498 | ORF3137 | 1.40E-01 | 2.756009006 | 0.289447139 |
| chr1_2468 | ORF3137 | 1.40E-01 | 2.009259898 | 0.031617376 |
| chr1_673 | ORF3137 | 1.40E-01 | -1.341763583 | 0.214728523 |
| chr1_1447 | ORF3137 | 1.40E-01 | 1.28420435 | 0.291496774 |
| chr1_2400 | ORF3137 | 1.39E-01 | 2.192141564 | 0.014346926 |
| chr1_1638 | ORF3137 | 1.39E-01 | 4.153776812 | 0.00021954 |
| chr2_747 | ORF3137 | 1.39E-01 | -1.250171526 | 0.239531092 |
| chr1_858 | ORF3137 | 1.39E-01 | 1.682001957 | 0.087935145 |
| chr1_1874 | ORF3137 | 1.39E-01 | 1.951908707 | 0.027579232 |
| chr1_2571 | ORF3137 | 1.39E-01 | -2.243773876 | 0.007781545 |
| chr1_3214 | ORF3137 | 1.38E-01 | 8.318458687 | 0.006452988 |
| chr1_1293 | ORF3137 | 1.38E-01 | -2.216110571 | 0.007193408 |
| chr1_1368 | ORF3137 | 1.38E-01 | 1.644770667 | 0.180273433 |
| chr1_3268 | ORF3137 | 1.38E-01 | 2.094997901 | 0.131404666 |
| chr1_710 | ORF3137 | 1.37E-01 | 2.8097623 | 0.252466674 |
| chr1_1366 | ORF3137 | 1.37E-01 | 2.335113359 | 0.007944064 |
| chr1_25 | ORF3137 | 1.37E-01 | 2.146381483 | 0.083824916 |
| chr2_539 | ORF3137 | 1.36E-01 | 5.789187003 | 0.0227505 |
| chr1_892 | ORF3137 | 1.36E-01 | 5.529959217 | 0.163899264 |
| chr1_2387 | ORF3137 | 1.36E-01 | 2.704408469 | 0.010837983 |
| plasmid1_154 | ORF3137 | 1.36E-01 | 1.573226419 | 0.114339092 |
| chr2_199 | ORF3137 | 1.35E-01 | -1.328872881 | 0.210964671 |
| chr1_3001 | ORF3137 | 1.35E-01 | 3.119929366 | 0.000101211 |
| chr1_2017 | ORF3137 | 1.35E-01 | 1.621561584 | 0.180592646 |
| chr1_2363 | ORF3137 | 1.35E-01 | 1.744706791 | 0.062698068 |
| chr1_1798 | ORF3137 | 1.34E-01 | 2.299415691 | 0.016902937 |
| plasmid2_13 | ORF3137 | 1.34E-01 | -1.382462554 | 0.16902602 |
| chr1_677 | ORF3137 | 1.34E-01 | 1.226984598 | 0.265236476 |
| chr1_3194 | ORF3137 | 1.34E-01 | 1.959623166 | 0.083038697 |
| plasmid3_12 | ORF3137 | 1.34E-01 | -1.131757285 | 0.295317832 |
| chr1_970 | ORF3137 | 1.33E-01 | -1.594251311 | 0.112357726 |
| chr1_2714 | ORF3137 | 1.33E-01 | 1.458401318 | 0.153408779 |
| chr1_1668 | ORF3137 | 1.33E-01 | -1.608418647 | 0.131404666 |
| chr1_2376 | ORF3137 | 1.33E-01 | 2.240008197 | 0.025126833 |
| chr1_1182 | ORF3137 | 1.33E-01 | 1.235950002 | 0.297420763 |
| chr1_2373 | ORF3137 | 1.32E-01 | 2.874893235 | 0.072271814 |
| chr1_1398 | ORF3137 | 1.32E-01 | -1.171715703 | 0.275416768 |
| chr1_80 | ORF3137 | 1.32E-01 | 2.731189945 | 0.115054811 |
| plasmid1_120 | ORF3137 | 1.32E-01 | 2.435265425 | 0.00673766 |
| chr1_457 | ORF3137 | 1.32E-01 | 2.642884607 | 0.054944841 |
| chr1_2986 | ORF3137 | 1.32E-01 | 2.490100889 | 0.003235563 |
| chr1_2635 | ORF3137 | 1.31E-01 | 2.397217953 | 0.226585517 |
| chr2_957 | ORF3137 | 1.31E-01 | -1.510057143 | 0.1536535 |
| chr1_2662 | ORF3137 | 1.31E-01 | 1.871363337 | 0.177244868 |
| chr1_1836 | ORF3137 | 1.31E-01 | 1.609640719 | 0.174196282 |
| chr1_2282 | ORF3137 | 1.31E-01 | -1.458658104 | 0.276844897 |
| chr1_521 | ORF3137 | 1.29E-01 | -1.424182777 | 0.235847471 |
| chr1_2519 | ORF3137 | 1.29E-01 | 1.614089072 | 0.163899264 |
| chr1_556 | ORF3137 | 1.29E-01 | 1.319733866 | 0.239531092 |
| chr1_2097 | ORF3137 | 1.28E-01 | 1.495133492 | 0.144168181 |
| chr1_2971 | ORF3137 | 1.28E-01 | 2.212087711 | 0.010567106 |
| chr1_1207 | ORF3137 | 1.28E-01 | -1.180383518 | 0.291496774 |
| chr2_795 | ORF3137 | 1.27E-01 | 2.013844894 | 0.026979316 |
| chr1_1653 | ORF3137 | 1.26E-01 | -1.678738544 | 0.146469826 |
| chr2_1064 | ORF3137 | 1.26E-01 | -2.16700568 | 0.016157075 |
| chr1_3277 | ORF3137 | 1.26E-01 | 1.447049854 | 0.239531092 |
| chr2_540 | ORF3137 | 1.26E-01 | 3.36401742 | 0.196764159 |
| chr2_1035 | ORF3137 | 1.26E-01 | -2.053239728 | 0.114675898 |
| chr2_652 | ORF3137 | 1.25E-01 | -1.873181755 | 0.291496774 |
| chr1_2587 | ORF3137 | 1.25E-01 | -1.67390597 | 0.064179977 |
| chr1_1334 | ORF3137 | 1.25E-01 | 1.723811778 | 0.067404078 |
| chr1_631 | ORF3137 | 1.25E-01 | 7.51578366 | 0.027458169 |
| chr1_3303 | ORF3137 | 1.24E-01 | 2.748293338 | 0.020022857 |
| chr1_1448 | ORF3137 | 1.23E-01 | 2.299932771 | 0.012405176 |
| chr1_3207 | ORF3137 | 1.23E-01 | 1.473319998 | 0.196764159 |
| chr1_1763 | ORF3137 | 1.23E-01 | 3.60706465 | 0.291295204 |
| chr1_3392 | ORF3137 | 1.22E-01 | -1.678005444 | 0.157646266 |
| chr1_385 | ORF3137 | 1.22E-01 | -1.896163676 | 0.028957505 |
| chr1_853 | ORF3137 | 1.22E-01 | 2.156821778 | 0.083079024 |
| chr1_1521 | ORF3137 | 1.22E-01 | 1.98104718 | 0.050630957 |
| plasmid1_99 | ORF3137 | 1.21E-01 | 1.932690459 | 0.237219717 |
| chr2_907 | ORF3137 | 1.21E-01 | -1.178903567 | 0.295317832 |
| chr1_3123 | ORF3137 | 1.21E-01 | 1.433185567 | 0.212703054 |
| chr1_1383 | ORF3137 | 1.20E-01 | 4.438041677 | 0.02473178 |
| chr1_2562 | ORF3137 | 1.20E-01 | 2.978088171 | 0.264462063 |
| chr1_2146 | ORF3137 | 1.20E-01 | 1.339707401 | 0.2988682 |
| plasmid1_47 | ORF3137 | 1.20E-01 | -3.562882612 | 1.9385E-06 |
| chr2_699 | ORF3137 | 1.20E-01 | -2.816822642 | 0.000392195 |
| chr1_848 | ORF3137 | 1.19E-01 | 2.798206255 | 0.196764159 |
| chr1_3053 | ORF3137 | 1.19E-01 | -2.36206132 | 0.008499461 |
| plasmid1_170 | ORF3137 | 1.19E-01 | -2.247094796 | 0.038053968 |
| chr1_2417 | ORF3137 | 1.19E-01 | 3.581179549 | 0.295109017 |
| chr1_3446 | ORF3137 | 1.18E-01 | 3.627357069 | 0.040620295 |
| chr1_2383 | ORF3137 | 1.18E-01 | 3.170996688 | 0.155824994 |
| chr1_2698 | ORF3137 | 1.18E-01 | 1.924775591 | 0.155271449 |
| chr1_1850 | ORF3137 | 1.17E-01 | 2.359788725 | 0.185361066 |
| chr1_2106 | ORF3137 | 1.17E-01 | 2.306197058 | 0.244595215 |
| chr1_3527 | ORF3137 | 1.16E-01 | 2.015050678 | 0.196764159 |
| chr2_290 | ORF3137 | 1.16E-01 | 1.694640674 | 0.095553214 |
| chr1_2379 | ORF3137 | 1.16E-01 | 2.584771054 | 0.267181035 |
| chr1_2485 | ORF3137 | 1.16E-01 | 1.920006665 | 0.084631577 |
| chr1_472 | ORF3137 | 1.16E-01 | 3.004614787 | 0.080477608 |
| chr1_788 | ORF3137 | 1.15E-01 | -1.725846068 | 0.05368412 |
| chr1_2870 | ORF3137 | 1.15E-01 | -1.462720048 | 0.202441314 |
| chr1_2289 | ORF3137 | 1.14E-01 | -1.875569372 | 0.300261754 |
| chr1_3401 | ORF3137 | 1.14E-01 | 1.664868366 | 0.121978679 |
| chr1_563 | ORF3137 | 1.13E-01 | 2.614711239 | 0.108279902 |
| chr1_4 | ORF3137 | 1.13E-01 | -1.955972699 | 0.0336778 |
| chr1_1847 | ORF3137 | 1.13E-01 | -1.517632087 | 0.148715217 |
| chr2_901 | ORF3137 | 1.12E-01 | -1.952037684 | 0.026714553 |
| chr1_1075 | ORF3137 | 1.12E-01 | 5.92589138 | 0.050630957 |
| chr1_436 | ORF3137 | 1.12E-01 | 2.963377239 | 0.169745733 |
| chr1_2026 | ORF3137 | 1.11E-01 | 1.582660629 | 0.107161379 |
| chr1_3445 | ORF3137 | 1.11E-01 | 3.11299513 | 0.032906602 |
| plasmid1_15 | ORF3137 | 1.10E-01 | -1.345899406 | 0.191592592 |
| chr1_1553 | ORF3137 | 1.10E-01 | -2.453757264 | 0.004359939 |
| chr1_2579 | ORF3137 | 1.10E-01 | 3.233117786 | 0.000769232 |
| chr1_826 | ORF3137 | 1.09E-01 | -2.029947242 | 0.02099268 |
| chr1_1563 | ORF3137 | 1.08E-01 | -2.621434724 | 0.000970731 |
| chr2_591 | ORF3137 | 1.08E-01 | 2.802346317 | 0.258992582 |
| chr1_1939 | ORF3137 | 1.08E-01 | 2.40809182 | 0.175289513 |
| chr2_432 | ORF3137 | 1.07E-01 | -3.403224645 | 1.21988E-05 |
| chr1_392 | ORF3137 | 1.07E-01 | 2.98416366 | 0.140880539 |
| chr1_3148 | ORF3137 | 1.07E-01 | -1.879551982 | 0.060089327 |
| chr1_1066 | ORF3137 | 1.06E-01 | 3.209084525 | 0.211431711 |
| chr1_466 | ORF3137 | 1.06E-01 | 3.775264532 | 0.174196282 |
| chr1_3387 | ORF3137 | 1.06E-01 | -1.788780585 | 0.054944841 |
| chr1_3529 | ORF3137 | 1.06E-01 | 4.289025024 | 2.57328E-07 |
| chr1_438 | ORF3137 | 1.06E-01 | 3.30312159 | 0.155824994 |
| chr1_1373 | ORF3137 | 1.04E-01 | 2.321208286 | 0.21472434 |
| chr1_2613 | ORF3137 | 1.03E-01 | 3.632074938 | 0.281354716 |
| chr1_909 | ORF3137 | 1.03E-01 | -1.528581194 | 0.15206757 |
| plasmid1_162 | ORF3137 | 1.03E-01 | -1.770151263 | 0.108231543 |
| chr1_544 | ORF3137 | 1.03E-01 | -1.979610795 | 0.192995894 |
| chr2_600 | ORF3137 | 1.03E-01 | 3.345659496 | 0.291496774 |
| chr1_1785 | ORF3137 | 1.01E-01 | 2.564641836 | 0.006496148 |
| chr1_2482 | ORF3137 | 9.93E-02 | 2.767549223 | 0.271205241 |
| chr1_713 | ORF3137 | 9.70E-02 | 1.967732495 | 0.196292526 |
| chr1_2388 | ORF3137 | 9.67E-02 | 2.594890336 | 0.20802795 |
| chr1_1736 | ORF3137 | 9.65E-02 | -2.87852128 | 0.220659929 |
| chr1_2551 | ORF3137 | 9.55E-02 | 1.311806483 | 0.267904964 |
| chr1_2051 | ORF3137 | 9.42E-02 | -3.096083567 | 0.06690536 |
| plasmid2_31 | ORF3137 | 9.39E-02 | -1.61872704 | 0.160311315 |
| chr1_1230 | ORF3137 | 9.34E-02 | -1.551308664 | 0.100709005 |
| chr1_1975 | ORF3137 | 9.26E-02 | 1.399372045 | 0.265105431 |
| chr1_3195 | ORF3137 | 9.25E-02 | 7.889892355 | 0.014764887 |
| chr1_3400 | ORF3137 | 9.24E-02 | -1.606057192 | 0.091255548 |
| plasmid1_30 | ORF3137 | 9.18E-02 | -2.157926196 | 0.008499461 |
| chr2_761 | ORF3137 | 9.12E-02 | -2.35496154 | 0.082728216 |
| chr1_3552 | ORF3137 | 8.98E-02 | 2.528964194 | 0.158054707 |
| chr1_1678 | ORF3137 | 8.98E-02 | -2.891657711 | 0.219218882 |
| chr1_2983 | ORF3137 | 8.95E-02 | 3.644738863 | 0.108231543 |
| chr1_2735 | ORF3137 | 8.93E-02 | 1.696467186 | 0.268582449 |
| chr1_1048 | ORF3137 | 8.93E-02 | 2.074415276 | 0.177244868 |
| chr1_3541 | ORF3137 | 8.92E-02 | -2.021838676 | 0.024329776 |
| chr1_1749 | ORF3137 | 8.70E-02 | -3.996589738 | 0.002701816 |
| chr2_1114 | ORF3137 | 8.68E-02 | 2.244231345 | 0.0269055 |
| chr1_3557 | ORF3137 | 8.63E-02 | 9.230334555 | 0.00110292 |
| chr1_477 | ORF3137 | 8.62E-02 | -1.647279258 | 0.15318918 |
| chr1_1467 | ORF3137 | 8.61E-02 | 1.610764401 | 0.265105431 |
| chr2_12 | ORF3137 | 8.52E-02 | -4.158547722 | 0.068058232 |
| plasmid1_68 | ORF3137 | 8.50E-02 | 4.108546207 | 3.25237E-06 |
| chr1_3210 | ORF3137 | 8.48E-02 | -2.383283999 | 0.003235563 |
| chr1_1508 | ORF3137 | 8.23E-02 | -2.39512837 | 0.009070388 |
| chr1_561 | ORF3137 | 8.08E-02 | -1.643595983 | 0.158054707 |
| plasmid1_7 | ORF3137 | 7.99E-02 | -1.608900118 | 0.150199974 |
| chr1_2645 | ORF3137 | 7.94E-02 | 4.555474996 | 0.088378086 |
| chr1_1420 | ORF3137 | 7.87E-02 | -2.40228363 | 0.003694929 |
| chr1_373 | ORF3137 | 7.38E-02 | 1.712665038 | 0.169232214 |
| chr1_2350 | ORF3137 | 7.02E-02 | -3.243501401 | 0.007152089 |
| chr1_22 | ORF3137 | 7.02E-02 | 3.221309336 | 0.098639591 |
| chr1_3537 | ORF3137 | 6.94E-02 | -2.518264715 | 0.016909592 |
| chr1_3226 | ORF3137 | 6.86E-02 | -2.108532105 | 0.187826877 |
| chr2_28 | ORF3137 | 6.49E-02 | -4.939953227 | 0.005986569 |
| chr1_1888 | ORF3137 | 5.77E-02 | -1.844948856 | 0.061312789 |
| chr1_1119 | ORF3137 | 5.57E-02 | -2.431085405 | 0.196764159 |

**Table S5. Amino acid sequences of IclR-family proteins included in this study**

| No. | Protein | Source | Sequence |
| --- | --- | --- | --- |
| 1 | PheR | *Sphingomonadaceae* | MNARKASQLTAKGDEIEHQANERLPPEDLSANDSPRKAIQSVEIGVRVLRALMDCPRDGAHLREVAEKSGLSRSQAHRYLLAFVNTGVVEQNVQNGRYALGEFSVRIGMAALARVEPVRIAGDNLASVLEELQTTGLLAVWGNYGPSVIRWIDGGLPLFTSLHVGSVLPLQASSTGILYLTHCSRTLTQPVIEQERASGLVVSDDELERQIVQARELGYATTQGTVVPGLSALSVPVFDSANRLVATMSVLSRVQDKHFYSKTKIEKILSAAIAASRAIGWQG |
| 2 | AttJ | *Agrobacterium tumefaciens* | MGQRGQVCQGKCMAEDQQSSQISDTVPALRRAVRILDLVAGSPRDLTAAELTRFLDLPKSSAHGLLAVMTELDLLARSADGTLRIGPHSLRWANGFLSHLDIVSTFNDHLAQRHDLDPYTVTLTVREGGEVVYIGCRNSAQPLGHTFRIGMRLPAPFTATGKILLSDLGPGELRMLFSQFPQPLTSRSVAGLSQLEEELALTRARGYSIDDGQIREGMLCIGAAIRDYSGAASAGIAISLIRSEASDEKIAYLGEELRTTANALSEKLGYRSQKD |
| 3 | CatR | *Rhodococcus opacus* | MTESDRDYIQSIERGFAVLLAFDAQRPNPTLAELATEAGLSRPAVRRILLTLQKLGYVAGSGGRWSLTPRVLSIGQHYSESHALIEAAMPRLLEVAEKTQESASLGVLDGADVVYAARVPVRRIMSINVSVGTRVPAYATSMGRALLAWAPADVVEQVVAESTFEKLGPETISTAAELDKELAKVREQGFALTSEELEKGLISLPAPVRDAGGTVVGVVACSTSSARNTPAQFREQAVPCVLAAAAALSTDMGFAG |
| 4 | DitR | *Pseudomonas abietaniphila* | MPRTQTDSNAPQRPDTAETTTDVQKGAVSVKPVSNAIRILRHLSQAGTPERSVDLARVLGINPSTCFNILRTLVMEDVVDFNPLSKRYSAGLGLARLVEQLVTQGQRVEFAKPLLQELAARLRVTVTLWRRMGPDRIVIVSSAASPTDVRIDMAEGQRLPILMGASGRLFATRMDLDEEKIRDDFEAIRWARPLPLKVYRQDVALAAERGWAVDDGYFSIGILAIAAPVFSPSGNIDFTVSAVLFRGQRDEEGIEEIGRTLVTFCAELSKVLF |
| 5 | Hmg | *Pseudomonas putida* | MSMTKASSSADNGKQKVRSAEVGTDILKALAELSPSTSLSRLSEHVQMPASKVHRYLQALIASGFAEQDAATNHYGLGREALRVGLAALGSIDVLKIAALPLSQLRDELNESCFIAVWGNQGATVVSIEPAVRAVTVVTQMGSVLPLLTSSTGLVFAAYLPERETVELRDRELAALQQSADDYQAVLAGIRERGLHHVHGLLMPGVDALSAPVFNAMGQVAAVMTVVGPTSIFHADEHGPAAQRLLAAARETSWRMGYSPAA |
| 6 | OhbR | *Pseudomonas aeruginosa* | MSVKELADEVDMSTSRTHRYLASLVRAGLIERDAASGRYDLGELTLQLGVAALNRLNPVAEASKTLRTLVNDVDQTAVLAVWSERGPVIAHWIHSSRPVIMNLNVGSILPILTSSSARVFLAFMPPNVLEATLDSALRALEPQAAKEFDMEKLRRQIAEVQRDHLARVAGAVAPGLLTASAPVLDAQGEPVAAMSLVATHTTDMERFEAAIPVLCRGAGQVSRRLGYRPRQPDEPSAS |
| 7 | GclR | *Escherichia coli* | MAKFAANLSMLFTEHPFIERFAQASHAGFHGVEYLFPYDFSTDELASQLHQHNLTQVLFNLPAGNWQEGERGIACHPSRAKEFQEGVCRAIEYAQALNCSQVNCLAGKHPGGYSHEQCHETLVENLRYAVDKLASHGIKLVLEAINTKDIPGFFVNNTRQALNIIYDVNHPDFRYQYDIYHMQIMEGNIATTIKNNLNNIEHIQLADNPGRHEPGTGEINYPWLLNYIDQIGYQGWIGCEYVPSTTTTESLRWLKNETQF |
| 8 | AllR | *Escherichia coli* | MTEVRRRGRPGQAEPVAQKGAQALERGIAILQYLEKSGGSSSVSDISLNLDLPLSTTFRLLKVLQAADFVYQDSQLGWWHIGLGVFNVGAAYIHNRDVLSVAGPFMRRLMLLSGETVNVAIRNGNEAVLIGQLECKSMVRMCAPLGSRLPLHASGAGKALLYPLAEEELMSIILQTGLQQFTPTTLVDMPTLLKDLEQARELGYTVDKEEHVVGLNCIASAIYDDVGSVVAAISISGPSSRLTEDRFVSQGELVRDTARDISTALGLKAH |
| 9 | GylR | *Streptomyces avermitilis* | MARNIQSLERAAAMLRLLAGGERRLGLSDIASSLGLAKGTAHGILRTLQQEGFVEQEAASGRYQLGAELLRLGTTYLDVHELRARALVWTDDLARSSGESVYLGVLHQQGVLIVHHVFRPDDSRQVLEVGAMQPLHSTALGKVLSAYDPVAHSEVLEAERKVFTPRTISELDQFEGVLDLTRARGYADDVEETWTGVASVSAPIHNRRRMPVGAVGITGAVERVCKDGQLRPDLIAAVRDCARAVSRDLGAGRF |
| 10 | HppR | *Rhodococcus globerulus* | MCRSGVKPRENRVKGVASESNGSSGKQPGSQTLARGLQALELVATTPDGMTIQEVADALGVHRTIASRLLTTVADFRLIKRGADGKYRAGGGLAALARDLYAGLRDEATPLLRRLANSLGASVALFVAEGSEAVAVAVVEPKNARYWVSFREGGRHPIDRGAAGYALLAGQPPVPGESSRVTDARRDGYVVSYGEVEPGYWGLGYR |
| 11 | IclR | *Escherichia coli* | MVAPIPAKRGRKPAVATAPATGQVQSLTRGLKLLEWIAESNGSVALTELAQQAGLPNSTTHRLLTTMQQQGFVRQVGELGHWAIGAHAFMVGSSFLQSRNLLAIVHPILRNLMEESGETVNMAVLDQSDHEAIIIDQVQCTHLMRMSAPIGGKLPMHASGAGKAFLAQLSEEQVTKLLHRKGLHAYTHATLVSPVHLKEDLAQTRKRGYSFDDEEHALGLRCLAACIFDEHREPFAAISISGPISRITDDRVTEFGAMVIKAAKEVTLAYGGM |
| 12 | MhpR | *Comamonas testosteroni* | MNDYKDVRSLSRGLALLQAMNRAPGGIASTTALAQACDIHRTTVKRLMETLRAEGFVRRGEKDGQYYLTFAVRSLSEGLVDDAWVEQVALPLMRAAVPELLWPCDLGTVEGGFMVVRESTHRFSRLSQHRGMIGEKLPLFFTAMGRAYLASCSTDEKEGLLSLLAQRDDATGAMARDRASVERLIEETRQRGYGISDGDWHQQAPFGAVAVPLKCGRRLIGGLNLVFPKSAVAREELLARYLPRLKKLAVRMGKDVAPWLD |
| 13 | OhbR | *Pseudomonas aeruginosa* | MSVKELADEVDMSTSRTHRYLASLVRAGLIERDAASGRYDLGELTLQLGVAALNRLNPVAEASKTLRTLVNDVDQTAVLAVWSERGPVIAHWIHSSRPVIMNLNVGSILPILTSSSARVFLAFMPPNVLEATLDSALRALEPQAAKEFDMEKLRRQIAEVQRDHLARVAGAVAPGLLTASAPVLDAQGEPVAAMSLVATHTTDMERFEAA  IPVLCRGAGQVSRRLGYRPRQPDEPSAS |
| 14 | PcaR | *Pseudomonas putida* | MSDETLVNDPVNPEPARPASAAMAPPIVASPAKRIQAFTGDPDFMTSLARGLAVIQAFQERKRHLTIAQISHRTEIPRAAVRRCLHTLIKLGYATSDGRTYSLLPKVLTLGHAYLSSTPLAISAQPYLDRISDQLHEAANMATLEGDDILYIARSATVERLISVDLSVGGRLPAYCTSMGRILLAAMDDTSLREYLGRADLKARTSRTLHDPESLFACIQQVRAQGWCVVDQELEQGLRSIAVPIYDASGQVLAALNVSTHVGRVTRSELEQRFLPILLAASRDLCHQLFG |
| 15 | PcaR | *Rhodococcus opacus* | MTATEPTEKILPSPDYVQSLARGLAVIRCFDNRNQRRTLSDVARATDLTRATARRFLLTLVELGYVATDGSAFWLTPRVLELGYSYLSSLSLPEVAQPHLERLSHKVHESSSVSILDGADIVYVARVPVSRIMTVGITIGTRLPAYATSMGRVLLAGLPDDELDAYLEKLDIQRLTERTITARDDLKAAILAVRADGICVLDQELEAGLRSMAAPIRNASGLTVAAVNISTPAARYSLEDLHSDLIPSLRVTATDIEQDLATVNR |
| 16 | PcaR | *Rhodopseudomonas palustris* | MPKLKRTADDGPSESAEFIESLSRGLRVIEAFGAEKRPMTLSDVAKACGLPRATARRILLTLQSAGYVSSDDRLFALTPRVLGLASAYLASNQISAVLQPLMDRVSASAKEVCSLAILDGDDAVFVARASPARVFSAGIDLGYRLPLFCTSVGRVLLSRLDNDELTATINAMTLTKQTEDTITDKQTVIATIIADRTKGYSLVDREAEPGFRSVAVPIRRYDGTIVAAANIGAHVDRITTGEMIDRFLPLLQTMAEEARPLMM |
| 17 | PcaU | *Acinetobacter sp. ADP1* | MWSNMDDKKVKEEKILHNSTNKKIIRHEDFVAGISKGMAILDSFGTDRHRLNITMAAEKTGMTRAAARRHLLTLEYLGYLESDGHYFYLTPKILKFSGSYLGGAQLPKISQPLLNLLTTQTSLIYSVMVLDGYEAITIARSAAHQQTDRVNPYGLHLGNRLPAHATSAGKILLAYLDDHAQQEWLNQYPLQRLTKYTYTNNIDFLRLLSEIKEQGWCYSSEEHELGVHALAVPIYGQQSRVVAALNIVSPTMRTTKEYLIQHILPLLQETARELRNIL |
| 18 | PobR | *Acinetobacter* | MEQHHQYLAHPHSSEEIRTEDYIAGLAKGLALLEAFGIDRQRLNVTQVAERTGISRTAARRYLKTLKFLGYLDTDEHYFWLTHRVLRFSSSYLSSAHLPKVAQSFLNLLCAQTSLTFSIVVLDEHEVVPVARSYLPQQDNLRVSPYGMHLGNRLPAHATSTGKVLLSVLDREVQIEWIEKYGLKRLTPYTITDEHTFLETLDAVRQSDYCLSTEEHELGLIAIAVPVLNAQGLTIAALNCMSQTNRVQPQYLIDQVLPLLRNTANELRNL |
| 19 | TsaQ1 | *Comamonas testosteroni* | MATHVKTPDQLQESGTGLVHSLAKGLEILSCFSEGELLGNQQLVELTGLPKATVSRLTSTLVKLGYLQVDPRSRKLAMGARVLGLGVSVQRKLGLQRIARPHMEALSQRFGLTVTMGTRDRLSVVLLEVCRPPSLAQLVVNFDAGTHMPLSQTALGLASLVNSPVKDREQVIEGLRKQLGDQWVEARNRIERAHQEHERYGYIVSQRSLGRDVSGVAVGMVPMGSNTPYVFHMAGPSNQMPLSLMRSDMGPALKQMVQDIQAEMRAARPPKLVVPKEF |
| 20 | TtgT | *Pseudomonas putida* | MSDSEESSARHGGIQVIARAASIMRALGSHPQGLSLAAIAQVVDLPRSTVQRIINALGAEHLVEALGPSGGFRLGPAFGRLITQAQTDIISLVRPHLIALSEQVYESTCLLSLSGEKIYVLDRVVAERELRVVFPIGIHVPATAVSGGKVLLAELSEEAQQALLPDPLPVCTPRSVAREALLEQLKTIKSGGVADDHDEYIEGLCSYSVLLDTYLGHYSVSIVAPNSRATTRVAEFQQALQACKQNIEVTIGRAPREFAG |
| 21 | TtgV | *Pseudomonas putida* | MNQSDENIGKAGGIQVIARAASIMRALGSHPHGLSLAAIAQLVGLPRSTVQRIINALEEEFLVEALGPAGGFRLGPALGQLINQAQTDILSLVKPYLRSLAEELDESVCLASLAGDKIYVLDRIVSERELRVVFPIGINVPAAATAAGKVLLAALPDETLQAALGEQLPVLTSNTLGRKALVKQLSEVRQSGVASDLDEHIDGVCSFATLLDTYLGYYSLAIVMPSSRASKQSDLIKKALLQSKLNIERAIGRASKKA |
| 22 | PbaR | *Sphingobium wenxiniae strain* JZ-1 | MGTVDKALGLLGLFSIEEPQWTVEAAASRTGIPTSTAYRYFRSLNEAGLITDFSAGRYVIGPAVIHLDRVARGTDPLVLAAQDAMDNLINRGPDRSVVILARIFDRRVMCVDQRRKGYHPLTISYERGRPMPLYRGSVSKIILAHLSPRLIVRCFHDDRIDIEEAGLGSDLKSFRRNLRLIRRAGYSVTHGEVDKGVIGIAAPILSPNGDVFACLSLVVAEETTPEGSIEKLVALVRKEALAVTASLGLMSDN |
